# miR‐193b‐3p and miR‐346 Exert Antihypertensive Effects in the Rostral Ventrolateral Medulla

**DOI:** 10.1161/JAHA.124.034965

**Published:** 2024-06-27

**Authors:** Shuai Zhang, Xueping Wang, Tengteng Dai, Lei Tong, Gaojun Chen, Linping Wang, Zhangyan Ren, Haisheng Liu, Dongshu Du

**Affiliations:** ^1^ International Cooperation Laboratory of Molecular Medicine, Academy of Chinese Medical Sciences Zhejiang Chinese Medical University Hangzhou Zhejiang China; ^2^ College of Life Sciences Shanghai University Shanghai China; ^3^ College of Agriculture and Bioengineering Heze University Heze Shandong China; ^4^ Shaoxing Institute of Shanghai University Shaoxing Zhejiang China

**Keywords:** apoptosis, *Arhgef9*, hypertension, miR‐193b‐3p, miR‐346, rostral ventrolateral medulla, Hypertension

## Abstract

**Background:**

Rostral ventrolateral medulla (RVLM) neuron hyperactivity raises sympathetic outflow, causing hypertension. MicroRNAs (miRNAs) contribute to diverse biological processes, but their influence on RVLM neuronal excitability and blood pressure (BP) remains widely unexplored.

**Methods and Results:**

The RVLM miRNA profiles in spontaneously hypertensive rats were unveiled using RNA sequencing. Potential effects of these miRNAs in reducing neuronal excitability and BP and underlying mechanisms were investigated through various experiments. Six hundred thirty‐seven miRNAs were identified, and reduced levels of miR‐193b‐3p and miR‐346 were observed in the RVLM of spontaneously hypertensive rats. Increased miR‐193b‐3p and miR‐346 expression in RVLM lowered neuronal excitability, sympathetic outflow, and BP in spontaneously hypertensive rats. In contrast, suppressing miR‐193b‐3p and miR‐346 expression in RVLM increased neuronal excitability, sympathetic outflow, and BP in Wistar Kyoto and Sprague‐Dawley rats. Cdc42 guanine nucleotide exchange factor (*Arhgef9)* was recognized as a target of miR‐193b‐3p. Overexpressing miR‐193b‐3p caused an evident decrease in *Arhgef9* expression, resulting in the inhibition of neuronal apoptosis. By contrast, its downregulation produced the opposite effects. Importantly, the decrease in neuronal excitability, sympathetic outflow, and BP observed in spontaneously hypertensive rats due to miR‐193b‐3p overexpression was greatly counteracted by *Arhgef9* upregulation.

**Conclusions:**

miR‐193b‐3p and miR‐346 are newly identified factors in RVLM that hinder hypertension progression, and the miR‐193b‐3p/*Arhgef9*/apoptosis pathway presents a potential mechanism, highlighting the potential of targeting miRNAs for hypertension prevention.

**Nonstandard Abbreviations and Acronyms**
GO: Gene Ontology
HR: heart rate
KEGG: Kyoto Encyclopedia of Genes and Genomes
miRNA: microRNA
NC: negative control
RSNA: renal sympathetic nerve activity
RT: room temperature
RVLM: rostral ventrolateral medulla
SD: Sprague‐Dawley
SHR: spontaneously hypertensive rat
shRNA: short hairpin RNA
TH: tyrosine hydroxylase
UTR: untranslated region
WKY: Wistar Kyoto

**Research Perspective**

**What Is New?**

- This study demonstrated a transcriptome‐wide overview of abnormally expressed rostral ventrolateral medulla microRNAs in spontaneously hypertensive rats. In particular, the expression levels of miR‐193b‐3p and miR‐346 were significantly downregulated.
- Therapeutic overexpression of miR‐193b‐3p and miR‐346 in rostral ventrolateral medulla inhibits the progression of hypertension by decreasing neuronal excitability, sympathetic outflow, and blood pressure.
- miR‐193b‐3p mediates rostral ventrolateral medulla neuronal apoptosis and excitability through inhibitory targeting of Cdc42 guanine nucleotide exchange factor (*Arhgef9)*.

**What Question Should Be Addressed Next?**

- More studies are necessary to explore the roles of miR‐193b‐3p and miR‐346 in regulating the progression of hypertension in rostral ventrolateral medulla by interacting with other target genes and signaling cascades.

Hypertension is common chronic medical disorder characterized by consistent elevation of blood pressure (BP) ≥140/90 mm Hg.^1^ The complications of hypertension, such as heart failure and stroke, pose a serious threat to human health.^2^, ^3^ In 2010, approximately 1.39 billion people worldwide were affected by hypertension.^1^ By 2025, the anticipated figure for adults with hypertension is expected to increase to 1.56 billion.^4^ Considering the seriousness of the matter, the efficient prevention and treatment of hypertension have become a major focal point in the medical field. The autonomic nervous system, along with its sympathetic component, plays a vital role in the regulation of BP. Excessive activity of sympathetic nerves can have a substantial effect on the development of hypertension and related cardiovascular conditions,^5^ highlighting the importance of understanding autonomic function for effective hypertension control.

Cardiovascular centers, located within the medulla oblongata and hypothalamus, modulate sympathetic outflow and BP, encompassing the nucleus tractus solitarius, rostral ventrolateral medulla (RVLM), caudal ventrolateral medulla, and paraventricular nucleus.^6^, ^7^ Functioning as the ultimate conduit for central sympathetic outflow activity, the RVLM integrates afferent signals and operates as a primary efferent pathway through which sympathetic fibers extend to the spinal cord, consequently exerting an influence on BP.^8^ Ren et al concluded that Sirtuin 1 activation in the RVLM can reduce oxidative stress, alleviate sympathetic overdrive, and slow hypertension progression.^9^ Evidence revealed that knockdown of Interleukin enhancer binding factor 3 in RVLM significantly reduced sympathetic activation and BP through the Phosphatidylinositol‐3‐kinase/protein kinase B (PI3K‐Akt) pathway.^10^ The dysregulation of PDZ domain containing 8 in the RVLM disrupted the endoplasmic reticulum–mitochondrion signaling in neurons, which contributed to sympathetic overactivity and thereby caused stress‐induced hypertension.^11^ Aberrant expression of numerous genes in the RVLM is linked to the intricate control of sympathetic tone and BP. Hence, gaining a comprehensive understanding of the molecular mechanisms that govern gene expression in the RVLM is a critical step toward enhancing insights into hypertension.

Serving as epigenetic regulators, noncoding RNAs have been affirmed to orchestrate gene expression across transcriptional, posttranscriptional, and translational stages.^12^, ^13^ Their essential functions in normal physiological processes and pathological conditions have been evidenced. Among the most extensively researched noncoding RNAs are microRNAs (miRNAs), which are typically 19 to 25 nucleotides long. They modulate gene expression posttranscriptionally and contribute to diverse diseases. Dysregulation of peripheral miRNAs, such as miR‐214, miR‐140‐5p, miR‐122‐5p, and miR‐212‐5p, was identified in hypertension.^14^, ^15^, ^16^, ^17^ However, alterations in miRNA expression levels within RVLM and their roles in regulating sympathetic outflow and BP remain relatively unexplored. Previously, the authors reported significantly increased expression levels of miR‐335 and miR‐674‐3p in RVLM of a specific hypertensive rat model induced through the administration of electric foot‐shocks and noises.^18^ This outcome offers a rationale for examining other RVLM miRNAs, which could likely be associated with the reduction in sympathetic activity and BP.

In this research, RVLM tissues extracted from spontaneously hypertensive rats (SHRs) were subjected to deep RNA sequencing to analyze variations in the miRNA transcriptome upon high BP. SHR is an inbred genetic model that shares BP alterations observed in humans, proposed as a plausible model for exploring essential hypertension.^19^ The effects of miR‐193b‐3p and miR‐346 on RVLM neuronal excitability, sympathetic outflow, and BP in SHRs and normotensive rats (Wistar Kyoto [WKY] rats and Sprague‐Dawley [SD] rats) were determined. Whether miR‐193b‐3p targets Cdc42 guanine nucleotide exchange factor (*Arhgef9)* was investigated. Furthermore, the effects caused by miR‐193b‐3p and *Arhgef9* on neuronal apoptosis and hypertension progression were disclosed. This study was structured to systematically uncover RVLM miRNAs that may be linked to high BP, providing a valuable resource for exploring their functional roles in the treatment of hypertension.

## Methods

The data that support the findings of this study are available from the corresponding author on reasonable request. The miRNA sequencing raw data reported in this article have been deposited in the National Center for Biotechnology Information Sequence Read Archive under the accession number PRJNA1076371.

### Animals

A total of 260 SHRs, 200 WKY rats, and 60 SD rats were obtained from Beijing Vital River Laboratory Animal Technology (pathogen and virus free, male, 10 weeks old). The rats were housed under standard conditions by maintaining a 12:12‐hour light/dark cycle, a room temperature (RT) of 23±1 °C, 50% to 60% humidity, and given ad libitum access to chow food and water. They were arbitrarily allocated to different experimental groups. No inclusion and exclusion criteria were predetermined. In addition, 50 WKY rats within 24 hours of birth were procured from Beijing Vital River Laboratory Animal Technology for primary neuronal culture. The samples sizes used were similar to those used in previous studies^20^, ^21^ and were calculated using the formula n=2 standard deviation squared×power index/Δ^2^.^22^ With years of experience as the basis, a standard deviation of 0.15 was expected and a Δ of 0.2 was defined as goal of the study. The power index value (α=0.05, 2‐sided; β=0.2; power index =0.8) was taken from the book *Intuitive Biostatistics* (Oxford University Press, 1995). The Animal Care Ethics Committee of Shanghai University granted ethical approval for all animal experiments conducted in this study (approval number: ECSHU 2023‐067). The experiments were performed in accordance with the National Institutes of Health guidelines for the care and use of laboratory animals.^23^ For renal sympathetic nerve activity (RSNA) recording experiment, the rats were euthanized with an overdose of pentobarbital sodium (200 mg/kg, intravenous injection). For other experiments, the rats were euthanized by CO_2_ inhalation at 30% volume displacement rate.

### Isolation of Microglia, Astrocytes, and Neurons From the RVLM Tissues of Adult SHRs and WKY Rats

RVLM tissues from 13‐week‐old SHRs and WKY rats were extracted via punch‐out technique, following the guidelines provided in the standard rat atlas.^24^ Dissociation of RVLM tissues was performed using the Adult Brain Dissociation kit (mouse and rat; Miltenyi Biotec, Germany) according to the manufacturer's instructions. Then, the cell suspension was incubated with CD11b/c (Microglia) MicroBeads kit (rat, Miltenyi Biotec, Germany) to capture microglia. For astrocytes isolation, the astrocytes were purified according to the Zarei‐Kheirabadi et al methods.^25^ Following the Brewer and Torricelli's protocols, the neurons were extracted.^26^ The isolated microglia, astrocytes, and neurons were used for reverse transcription quantitative polymerase chain reaction (qPCR) and Western blot assays.

### Isolation of Primary Neurons From the RVLM Tissues of WKY Rats Born Within 24 Hours

RVLM tissues from WKY rats born within 24 hours were extracted via punch‐out technique, following the guidelines provided in the standard rat atlas.^24^ The tissues were dispersed in 0.125% trypsin (100 U DNase I added) for 20 minutes at RT. The cells were centrifuged at 1000 rpm for 10 minutes, and the supernatant was aspirated. The cell pellet was resuspended in DMEM/F12 medium supplemented with 10% FBS, 1% penicillin–streptomycin, and 1% l‐glutamine, and cultured in a 37 °C, 5% CO_2_ cell culture incubator for 6 hours. Then, the cells were cultured in neurobasal medium supplemented with 1% penicillin–streptomycin, 1% l‐glutamine, and 2% B27 in a 37 °C, 5% CO_2_ cell culture incubator for 7 to 10 days.

### B104 Cell Culture

The rat neuroblastoma B104 cell line, obtained from Shanghai Xuanya Biotechnology, was used for miRNA subcellular localization and dual‐luciferase reporter assay experiments. The authenticity of the cells was confirmed by the supplier. In a controlled environment of 37 °C with a 5% CO_2_ atmosphere, the cells were cultured in high‐glucose DMEM medium with the addition of 10% FBS, 1% penicillin–streptomycin, and 1% l‐glutamine.

### Measurement of Mean Arterial Pressure and Heart Rate

The Kaha Sciences radiotelemetry system (AD Instruments, New Zealand) was used to measure the mean arterial pressure (MAP) and heart rate (HR) of conscious rats. The procedure involved inserting a telemeter catheter into the abdominal aorta of the rats by using a tissue adhesive and a mesh patch while they were under inhalational anesthesia with isoflurane. The anesthesia process consisted of setting the oxygen flowmeter to 0.8 to 1.5 L/min and the isoflurane vaporizer to 3% to 5%. The telemeter body was fixed onto the peritoneum. After a recovery period of 1 week following the surgery, the recording process was initiated. The signals of MAP and HR were received by the Kaha Sciences SmartPad system (AD Instruments, New Zealand). Data collection was performed for a fixed period of 4 hours (8–12 am) each day by using the PowerLab system (AD Instruments).

### Recording of RSNA

Through incision surgery under inhalational anesthesia with isoflurane as mentioned above (oxygen flowmeter turned to 0.8–1.5 L/min; isoflurane vaporizer turned to 3%–5%), the left renal sympathetic nerve was surgically exposed and isolated. The nerve was then carefully positioned onto a pair of platinum–iridium electrodes. Kwik‐Sil gel (World Precision Instruments, United States) was applied to cover the nerve‐electrode complex. Amplification (1000×) and filtration (bandpass 100–3000 Hz) of the nerve activity were performed using a grass P55C preamplifier. The PowerLab system (AD Instruments) was used to acquire and integrate the nerve signal for a duration of 30 minutes. The maximum nerve activity was observed 1 to 2 minutes after an overdose of 200 mg/kg intravenous injection with pentobarbital sodium to the rats. Following euthanasia, the background noise level of the nerve activity was calculated approximately 20 to 30 minutes later. Baseline RSNA was determined as a percentage of the maximum activity after subtracting the background noise.

### Plasma Norepinephrine Determination

Cardiac puncture was performed on rats under inhalational anesthesia with isoflurane as mentioned above (oxygen flowmeter turned to 0.8–1.5 L/min; isoflurane vaporizer turned to 3%–5%) to obtain blood samples, which were then transferred into collection tubes containing ethylenediaminetetraacetic acid. The collected samples were promptly centrifuged at 1000*g* for 15 minutes at 4 °C. The resulting supernatant was used for analysis. The level of plasma norepinephrine was determined using an ELISA kit (FineTest, China) following the guidelines provided by the manufacturer.

### Immunofluorescence

Rats were anesthetized with an intraperitoneal injection of pentobarbital sodium (50 mg/kg) and subjected to transcardial perfusion with 200 mL heparinized saline, followed by 200 mL of freshly prepared 4% paraformaldehyde through the ascending aorta. The brains were extracted and fixed in 4% paraformaldehyde at RT for 12 hours. Subsequently, they were subjected to overnight dehydration in 20% sucrose at 4 °C, followed by an additional overnight dehydration in 30% sucrose at 4 °C. Frozen coronal sections of RVLM, measuring 30 μm in thickness, were obtained using a cryostat (Thermo Scientific, United States). The sections were subjected to permeabilization with 0.3% Triton X‐100 for 15 minutes, followed by blocking with QuickBlock buffer (Beyotime, China) for 30 minutes. Following the blocking step, the sections were subjected to an overnight incubation at 4 °C with the following primary antibodies: rabbit monoclonal c‐Fos (9F6, 1:500; Cell Signaling Technology, United States), mouse monoclonal Caspase 3 antibody (1:50; Santa Cruz, United States), mouse monoclonal tyrosine hydroxylase (TH) (F‐11, 1:100; Santa Cruz), and rabbit monoclonal NeuN antibody (1:600; Abcam, United States). On the following day, the sections were subjected to a 2‐hour incubation at RT with the following secondary antibodies: Alexa Fluor 594‐conjugated AffiniPure Goat Anti‐Rabbit immunoglobulin G (H+L; 1:400; Jackson ImmunoResearch, United States) and fluorescein isothiocyanate‐conjugated AffiniPure Goat Anti‐Mouse immunoglobulin G (H+L; 1:200; Jackson ImmunoResearch). Fluorescent signals were observed using a confocal laser scanning microscope (Zeiss, Germany).

### miRNA Sequencing

TRIzol reagent (Thermo Scientific) was used to isolate the total RNA from RVLM tissues. A total of 8 sequencing libraries were prepared, comprising 4 libraries for WKY rats and 4 libraries for SHRs. The libraries were generated using the TruSeq Small RNA Sample Prep Kit (Illumina, United States) following the instructions provided by the manufacturer. After sequencing, the raw data were processed using the ACGT101‐miR program (LC Sciences, China) to eliminate adapter dimers, low‐quality reads, common RNA families (such as messenger RNA (mRNA), ribosomal RNA, transfer RNA, small nuclear RNA, and small nucleolar RNA), and repeats to obtain clean reads. Subsequently, the clean reads with lengths ranging from 18 nucleotides to 26 nucleotides (valid reads) were compared against the pre‐miRNAs of rats and other species available in miRBase 22.0, as well as the rat genome to identify miRNAs. Length variation (≤3 nucleotide) at the 3′ end was allowed in the alignment. On the basis of the basic local alignment search tool findings, the miRNAs were categorized into 2 groups: gp1 and gp2. In gp1, the reads were aligned with mature rat miRNAs situated in the hairpin arms of pre‐miRNAs specific to rats present in miRBase 22.0 or with the opposite hairpin arms of rat pre‐miRNAs from miRBase 22.0 that does not harbor the annotated mature miRNAs. In gp2, the remaining reads were aligned with hairpin arms of pre‐miRNAs from other species in miRBase 22.0, and then these matched pre‐miRNAs can be aligned with the rat genome. miRNA expression levels (norm values) were estimated by ACGT101‐miR program (LC Sciences) as described by Li et al.^27^ Differentially expressed analysis was conducted using the edgeR package between the 2 groups.

### Reverse Transcription‐qPCR

Total RNA was extracted from cells and tissues by using TRIzol reagent (Thermo Scientific). RNA (1 μg) was reverse‐transcribed into cDNA by using Hifair V Reverse Transcriptase (Yeasen, China). Reverse transcription‐qPCR was performed using Hieff SYBR Green Master Mix (Yeasen) on the LightCycler 480 system (Roche, United States). The primer pairs are listed in Table S1. The data were normalized to U6 small nuclear 1 (U6) and Glyceraldehyde‐3‐phosphate dehydrogenase (GAPDH) by using the 2^−∆∆Ct^ method.

### Subcellular Localization of miR‐193b‐3p and miR‐346

The cytoplasmic and nuclear RNAs from B104 cells were isolated and purified using the Cytoplasmic & Nuclear RNA Purification Kit (Norgen Biotek, United States) following the instructions provided by the manufacturer. Reverse transcription‐qPCR was used to determine the expression levels of miR‐193b‐3p and miR‐346 in the nuclear and cytoplasmic fractions. GAPDH and U6 functioned as normalizing cytoplasmic and nuclear controls, respectively.

### Intra‐RVLM Microinjection

The rats, 11 to 13 weeks old, weighing 350 to 400 g, were subjected to inhalational anesthesia with isoflurane as previously described (oxygen flowmeter turned to 0.8–1.5 L/min; isoflurane vaporizer turned to 3%–5%) and positioned in a prone position with their heads secured in a stereotaxic apparatus (RWD Life Science, China). An incision along the midline was performed to expose the skull. The alignment of the bregma and λ points was achieved to ensure they were in the same horizontal plane. pLV‐EF1a‐EGFP > *Arhgef9* plasmid (pLV‐*Arhgef9*, lentiviral vector‐mediated *Arhgef9* overexpression, >10^9^ TU/mL) was synthesized by GenePharma (China). The agomir and antagomir of the miRNAs (miR‐193b‐3p, miR‐346, and miR‐322‐3p) and the negative controls were synthesized by GenePharma (Table S2), and they were diluted to a concentration of 10 nmol/μL in accordance with the guidelines provided by the manufacturer. Following the guidance of the rat brain atlas,^24^ they were injected into both sides of the RVLM at a volume of 0.05 μL per side by using a glass micropipette. The injection site was located 3.7 to 4.0 mm caudal to the lambdoid suture, 2 mm lateral to the midline, and 8.0 mm ventral to the surface of the dura. The authors' previous study included a diagram illustrating the process of verifying the accurate placement of RVLM, along with a detailed description.^20^ Next, 0.05 μL of 2% pontamine sky blue was injected into RVLM to identify the injection site (Figure S1). After the injection, the surgical incision was closed using sutures.

### Gene Ontology and *Kyoto Encyclopedia of Genes and Genomes* Survey

The TargetScan and miRanda tools were used to search for potential target genes.^28^, ^29^ For TargetScan, a pairing score threshold of ≥50 was applied. For miRanda, the parameter used was a free energy (ΔG) cutoff of ≤−10 kcal/mol. 1.13. The gene ontology (GO) survey was conducted using the GOseq R package^30^ to annotate the target genes of miR‐193b‐3p and miR‐346. KEGG orthology based annotation system software^31^ was used to perform *Kyoto Encyclopedia of Genes and Genomes* (KEGG) enrichment analysis on the target genes of miR‐193b‐3p and miR‐346.

### Western Blot

The extraction and isolation of total proteins were performed using RIPA buffer containing 1% PMSF. Proteins were separated on 10% SDS‐PAGE, transferred to polyvinylidene fluoride membranes (Bio‐Rad, United States), and then blotted with primary antibodies, namely, rabbit polyclonal ARHGEF9‐ (1:500; Zen‐Bio, China), rabbit polyclonal BCL2 (1:1000; ABclonal, China), rabbit monoclonal Bax (1:1000; Abways, China), rabbit monoclonal caspase 3 (1:500, Zen‐Bio), and mouse monoclonal HRP‐conjugated GAPDH (1:5000; Proteintech, United States), followed by secondary antibodies, including goat anti‐rabbit immunoglobulin G‐HRP (1:3000; Cell Signaling Technology) and horse anti‐mouse immunoglobulin G‐HRP (1:3000; Cell Signaling Technology).The protein bands were detected by super electrogenerated chemiluminescence detection reagent (Yeasen). The internal control used in the study was GAPDH.

### Cell Transfection

As shown in Table S2, the miR‐193b‐3p agomir (GenePharma), agomir negative control (NC) (GenePharma), miR‐193b‐3p antagomir (GenePharma), antagomir NC (GenePharma), and pLV‐*Arhgef9*‐short hairpin RNA (pLV‐*Arhgef9*‐short hairpin RNA [shRNA], a lentivirus containing shRNA targeting *Arhgef9*; GenePharma) were transfected into the RVLM primary neurons by using the Lipofectamine 8000 transfection reagent (Beyotime) following the guidelines provided by the manufacturer. Following a 48‐hour transfection period, the cells were collected for further experimentation.

### Dual‐Luciferase Reporter Assay

The pmiRGLO dual‐luciferase miRNA target expression vector was used to construct *Arhgef9*‐wild‐type‐3′ untranslated region (UTR)/mutant‐3′ UTR by synthesizing and incorporating the potential wild‐type and mutant binding sites of miR‐193b‐3p in the *Arhgef9* 3′ UTR. Cotransfection of the recombinant reporter plasmids with miR‐193b‐3p agomir (Table S2; GenePharma) or agomir NC (GenePharma) was performed in B104 cells. After 48 hours, the relative luciferase activity was assessed using the dual‐luciferase reporter assay kit (Yeasen) in accordance with the manufacturer's instructions.

### TdT‐Mediated dUTP‐Biotin Nick End‐Labeling Assay

For the detection of cell apoptosis, the YF 488 1.17. TdT‐mediated dUTP‐biotin nick end‐labeling (TUNEL) apoptosis detection kit (UElandy, China) was used, following the instructions provided by the manufacturer. RVLM sections that were frozen underwent fixation with 4% paraformaldehyde in PBS for a duration of 30 minutes at RT, followed by 2 washes with PBS. Subsequently, the sections were treated with 20 μg/mL proteinase K in PBS for 20 minutes at RT. Afterward, the sections underwent a 5‐minute incubation period at RT in the presence of TUNEL equilibration buffer, followed by 2 hours of incubation at 37 °C in the dark with TUNEL reaction buffer (including TdT enzyme). The signals were captured using a confocal laser scanning microscope (Zeiss, Germany).

### Flow Cytometry

The apoptosis of RVLM primary neurons was evaluated by using the Annexin V‐Alexa Fluor 647/PI apoptosis detection kit (Yeasen) in accordance with the guidelines provided by the manufacturer. The RVLM primary neurons were transfected with either miR‐193b‐3p antagomir, antagomir NC, or miR‐193b‐3p antagomir + pLV‐*Arhgef9*‐shRNA. Following a 48‐hour incubation period, the cells were harvested, washed 2 times with cold PBS, and resuspended in 1× binding buffer. Subsequently, the cells were incubated in the dark at RT with Alexa Fluor 647‐Annexin V and PI for a duration of 15 minutes. Upon adding 1× binding buffer to each well, the apoptotic cells were quantified using a flow cytometer (Beckman, United States).

### Cell Counting Kit‐8 Assay

Cell viability was assessed using the Cell Counting Kit‐8 assay kit (Bimake, China). The RVLM primary neurons were transfected with miR‐193b‐3p antagomir or antagomir NC or cotransfected with miR‐193b‐3p antagomir + pLV‐*Arhgef9*‐shRNA for 24, 48, and 72 hours. Subsequently, the Cell Counting Kit‐8 working solution was introduced into each well and coincubated for 1 hour. Lastly, the absorbance at a wavelength of 450 nm was measured using a LabServ K3 microplate reader (Thermo Scientific).

### Statistical Analysis

During the process of data collection, the investigators were blinded to ensure impartiality. Statistical analyses were performed using GraphPad Prism software (version 9.1). Data are presented as mean±SEM. When n<6, nonparametric tests were used to compare groups. When n≥6, the Shapiro‐Wilk test was applied to verify normality distribution. If the data followed a normal distribution, parametric tests were used for group comparison; otherwise, nonparametric tests were applied. Statistically significant differences between 2 groups were evaluated by unpaired 2‐tailed Student *t* test (parametric data) or Mann‐Whitney *U* test (nonparametric data). For comparisons involving >2 groups, 1‐way ANOVA with post hoc Bonferroni test was used for parametric data, whereas the Kruskal‐Wallis test with post hoc Dunn test was used for nonparammetric data. The important pairwise post hoc *P* values are shown in Table S3. A significance level of *P*<0.05 was used to determine statistical significance.

## Results

### miR‐193b‐3p, miR‐346, and miR‐322‐3p Expression Levels Were Significantly Downregulated in the RVLM of SHRs

**Figure 1:**
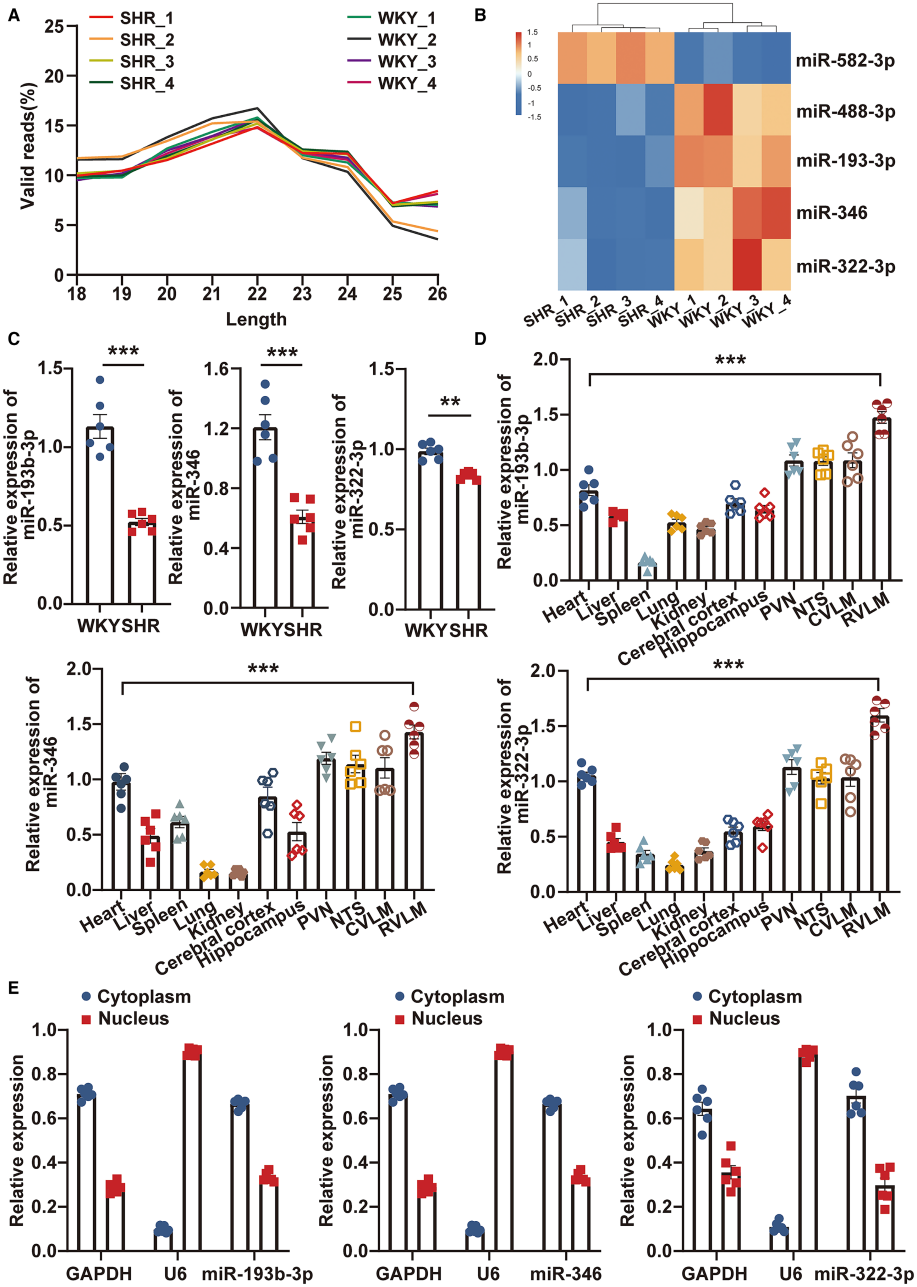
miR‐193b‐3p, miR‐346, and miR‐322‐3p were down‐expressed in RVLM of SHRs. **A**. Distribution of sequence lengths for valid reads was analyzed in SHR and WKY groups. **B**, Cluster analysis was conducted on the top 5 differentially expressed miRNAs determined by miRNA sequencing in SHRs and WKY rats. **C**, Relative expression levels of the 3 specific miRNAs, mentioned in (**B**), were assessed using RT‐qPCR in the 2 groups. **D**, Expression levels of miR‐193b‐3p, miR‐346, and miR‐322‐3p in 11 tissues of WKY rats were detected using RT‐qPCR. **E**, Subcellular localization of miR‐193b‐3p, miR‐346, and miR‐322‐3p was examined in B104 cells by using RT‐qPCR. N=6 rats per group (**C** and **D**). N=6 of independent cell culture preparations (**E**). **P<0.01. ***P<0.001. CVLM indicates caudal ventrolateral medulla; GAPDH, Glyceraldehyde‐3‐phosphate dehydrogenase; miRNA, microRNA; NTS, nucleus tractus solitarius; PVN, paraventricular nucleus; RT‐qPCR, reverse transcription‐quantitative polymerase chain reaction; RVLM, rostral ventrolateral medulla; SHR, spontaneously hypertensive rat; U6, RNA, U6 small nuclear 1; and WKY, Wistar Kyoto.

SHRs and WKY rats at 13 weeks of age were assessed for their MAP, HR, sympathetic outflow, and RVLM neuronal excitability levels. A considerable elevation was observed in the levels of MAP, HR, RSNA, and plasma norepinephrine and the population of c‐Fos‐positive TH+ neurons in SHRs when compared with WKY rats (Figure S2A through S2D). High‐throughput RNA sequencing was used to examine the miRNA expression profiles in RVLM between the 2 groups. After sequencing and quality control were conducted, a total of 49 233 758 valid reads were acquired. Among them, 22 870 849 reads were from the SHRs, and 26 362 909 reads were from the WKY rats. For both groups, the majority of the valid reads had lengths of 20 to 24 nucleotides (Figure 1A). The assembly process yielded a total of 637 miRNAs, which were then subjected to subsequent analysis. One hundred thirty miRNAs were identified as differentially expressed, with statistical significance achieved at *P*<0.05. A heatmap was generated to visually represent the top 5 differentially expressed miRNAs (Figure 1B). The results obtained through reverse transcription‐qPCR analysis exhibited a consistent expression pattern for 3 miRNAs (miR‐193b‐3p, miR‐346, and miR‐322‐3p) when compared with the RNA sequencing findings depicted in Figure 1B (Figure 1C). However, the analysis revealed a lack of differential expression for miR‐582‐3p and miR‐488‐3p, contradicting the RNA sequencing data (Figure S3). This discrepancy may be attributed to biological variations among the samples. Reverse transcription‐qPCR was performed to investigate the expression of miR‐193b‐3p, miR‐346, and miR‐322‐3p in neurons, microglia, and astrocytes isolated from the RVLM tissues of 13‐week‐old SHRs and WKY rats. The expression levels of miR‐193b‐3p and miR‐322‐3p were significantly lower in neurons of the SHRs than that in the WKY rats (Figure S4A). No significant differences were observed in the expression levels of miR‐193b‐3p and miR‐322‐3p between the 2 groups in RVLM microglia and astrocytes (Figure S4B and S4C). The expression level of miR‐346 was significantly lower in neurons and astrocytes of SHRs than that in WKY rats (Figure S4A and S4C). No significant difference was observed in the expression level of miR‐346 between the 2 groups in RVLM microglia (Figure S4B). Reverse transcription‐qPCR was performed to determine the tissue‐specific expression of miR‐193b‐3p, miR‐346, and miR‐322‐3p. The results showed that these miRNAs were expressed at high levels in the RVLM tissue of both SHRs and WKY rats (Figure S5 and Figure 1D). No significant differences were observed in the expression levels of miR‐193b‐3p, miR‐346, and miR‐322‐3p between the 2 groups in other cardiovascular centers (nucleus tractus solitarius, caudal ventrolateral medulla, and paraventricular nucleus (Figure S6). The subcellular localization analysis using reverse transcription‐qPCR demonstrated that miR‐193b‐3p, miR‐346, and miR‐322‐3p were primarily localized in the cytoplasmic fraction (Figure 1E). These findings indicated that the changes of miR‐193b‐3p, miR‐346, and miR‐322‐3p in RVLM may potentially participate in the neurogenic cause of hypertension and exert protective effects against high BP.

### Upregulation of miR‐193b‐3p and miR‐346 in RVLM Had Antihypertensive Effects in SHRs

**Figure 2:**
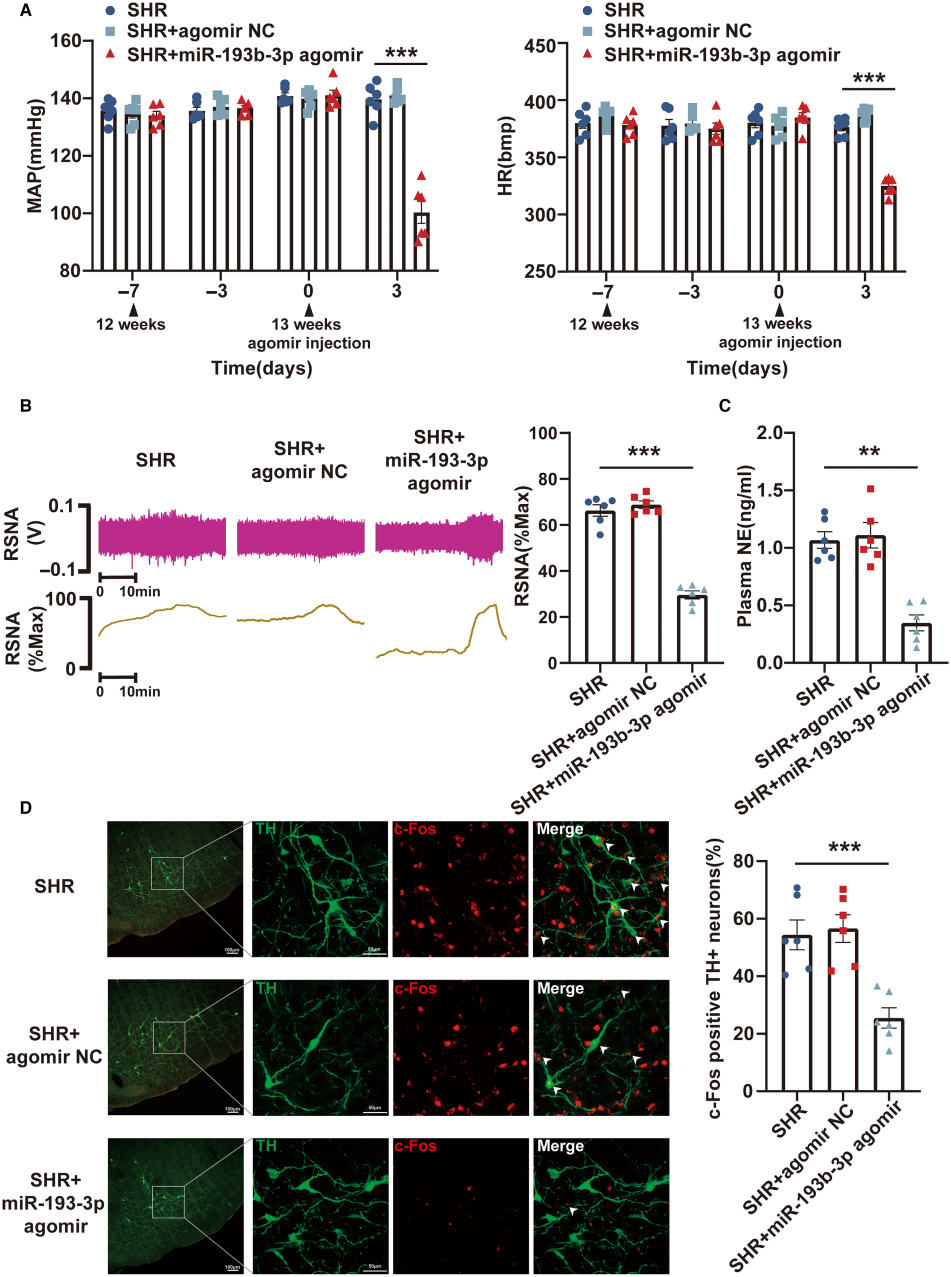
Overexpression of miR‐193b‐3p in RVLM improved hypertension in SHRs. **A**, MAP and HR in SHRs were measured by radiotelemetry following overexpression of miR‐193b‐3p. **B** and **C**, Sympathetic outflow in SHRs after miR‐193b‐3p overexpression was assessed by RSNA recording and ELISA. **D**, RVLM neuronal excitability in SHRs was evaluated by immunostaining after overexpression of miR‐193b‐3p. N=6 rats per group (**A** through **D**). **P<0.01. ***P<0.001. HR indicates heart rate; MAP, mean arterial pressure; NC, negative control; NE, norepinephrine; RSNA, renal sympathetic nerve activity; RVLM, rostral ventrolateral medulla; SHR, spontaneously hypertensive rat; and TH, tyrosine hydroxylase.

**Figure 3:**
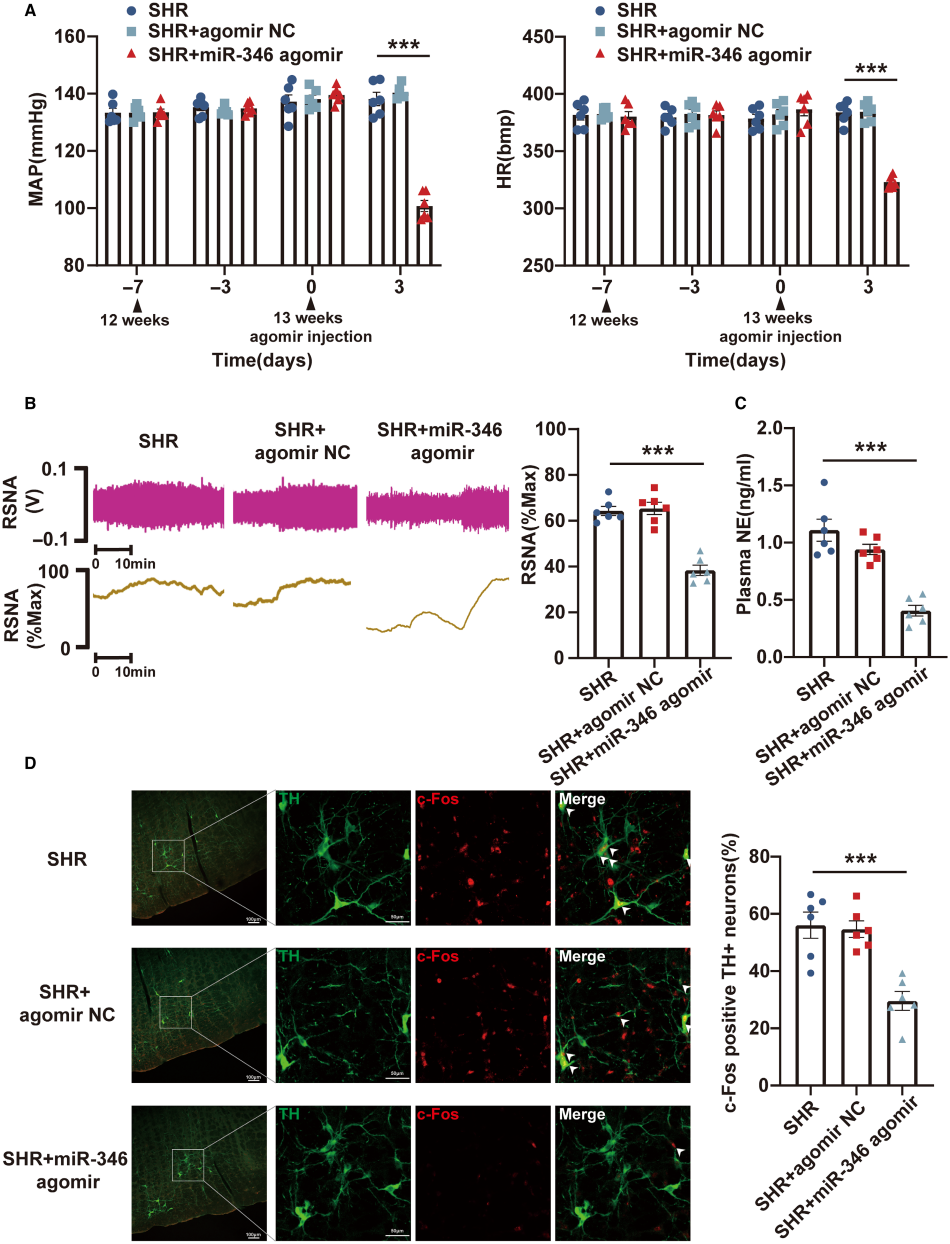
Upregulation of miR‐346 in the RVLM‐attenuated hypertension in SHRs. **A**, Radiotelemetry was used to assess the changes in MAP and HR in SHRs following upregulation of miR‐346. **B** and **C**, RSNA recording and ELISA were used to uncover the influence of miR‐346 upregulation on sympathetic outflow in SHRs. **D**, Immunostaining was used to evaluate the excitability of RVLM neurons in SHRs after upregulation of miR‐346. N=6 rats per group (**A** through **D**). ***P<0.001. HR indicates heart rate; MAP, mean arterial pressure; NC, negative control; NE, norepinephrine; RSNA, renal sympathetic nerve activity; RVLM, rostral ventrolateral medulla; SHR, spontaneously hypertensive rat; and TH, tyrosine hydroxylase.

A gain‐of‐function system was established by introducing miR‐193b‐3p agomir, miR‐346 agomir, and miR‐322‐3p agomir to examine their potential antihypertensive functions in SHRs. The RVLM of the 13‐week‐old SHRs was subjected to bilateral microinjection with the 3 agomirs. After 72 hours of microinjection, the overexpression efficiency was evaluated through reverse transcription‐qPCR (Figure S7A). The results from radiotelemetry showed a significant reduction in MAP and HR levels in SHRs with overexpression of miR‐193b‐3p (Figure 2A) and miR‐346 (Figure 3A). The RSNA recording and ELISA revealed a marked decrease in the RSNA and plasma norepinephrine levels of SHRs following the upregulation of miR‐193b‐3p (Figure 2B and 2C) and miR‐346 (Figure 3B and 3C). Immunofluorescence assay provided further insights into the effects of miR‐193b‐3p and miR‐346 on neuronal excitability, revealing a significant decrease in the proportion of c‐Fos‐positive TH+ neurons in the RVLM of SHRs upon miR‐193b‐3p (Figure 2D) and miR‐346 overexpression (Figure 3D). The MAP and HR of SHRs were not affected by miR‐322‐3p upregulation (Figure S8). Taken together, the results highlighted the potential of miR‐193b‐3p and miR‐346 to alleviate RVLM neuronal excitability, attenuate sympathetic tone, and participate in the neurogenic regulatory processes associated with hypertension.

### Knockdown of miR‐193b‐3p and miR‐346 in RVLM Resulted in the Development of Hypertension in WKY Rats

**Figure 4:**
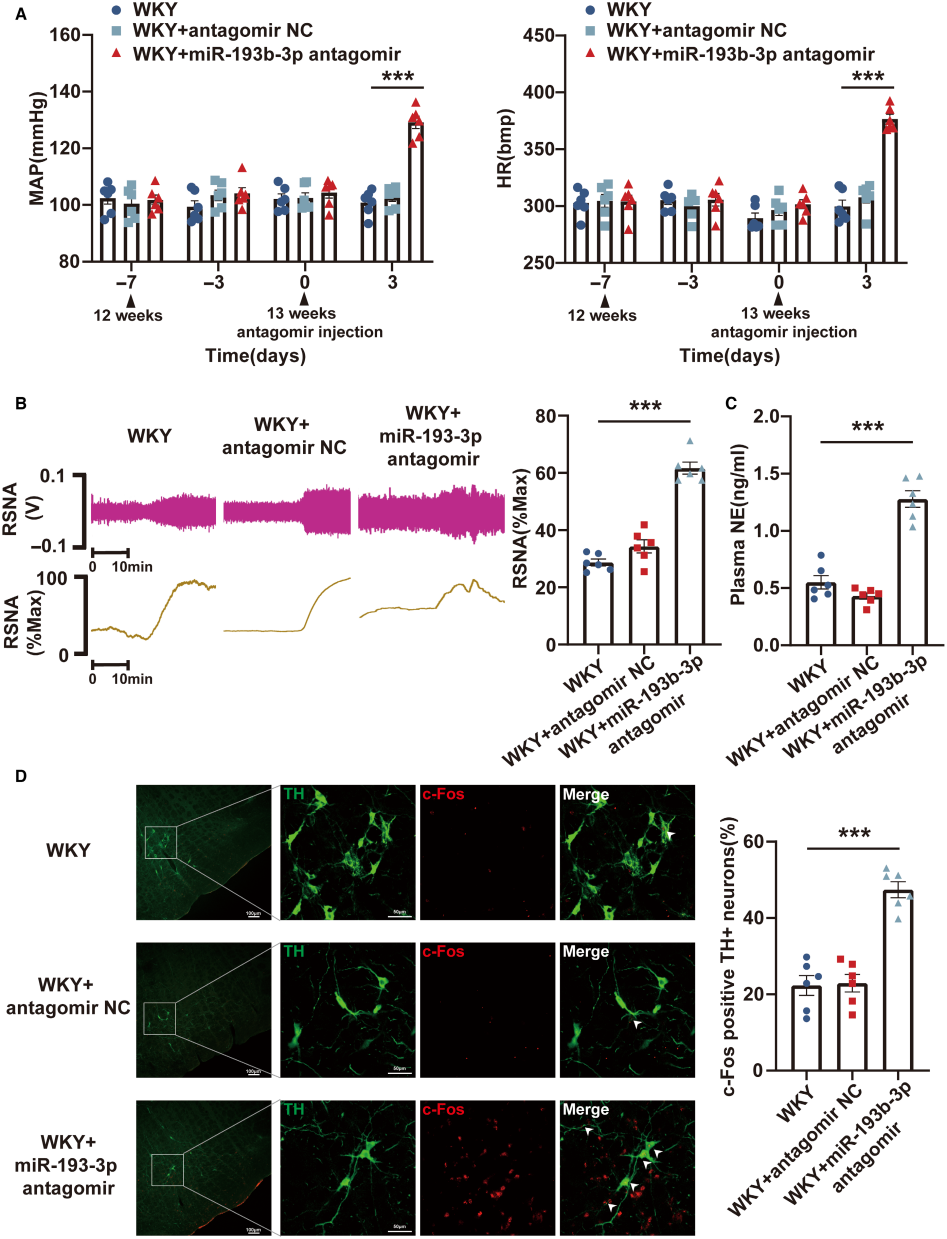
miR‐193b‐3p knockdown in RVLM promoted the progression of hypertension in WKY rats. **A**, MAP and HR in WKY rats after the knockdown of miR‐193b‐3p was measured using radiotelemetry. **B** and **C**, Sympathetic outflow in WKY rats after miR‐193b‐3p knockdown was assessed through RSNA recording and ELISA. **D**, RVLM neuronal excitability in WKY rats was monitored by immunostaining after knockdown of miR‐193b‐3p. N=6 rats per group (**A** through **D**). ***P<0.001. HR indicates heart rate; MAP, mean arterial pressure; NC, negative control; NE, norepinephrine; RSNA, renal sympathetic nerve activity; RVLM, rostral ventrolateral medulla; TH, tyrosine hydroxylase; and WKY, Wistar Kyoto.

**Figure 5:**
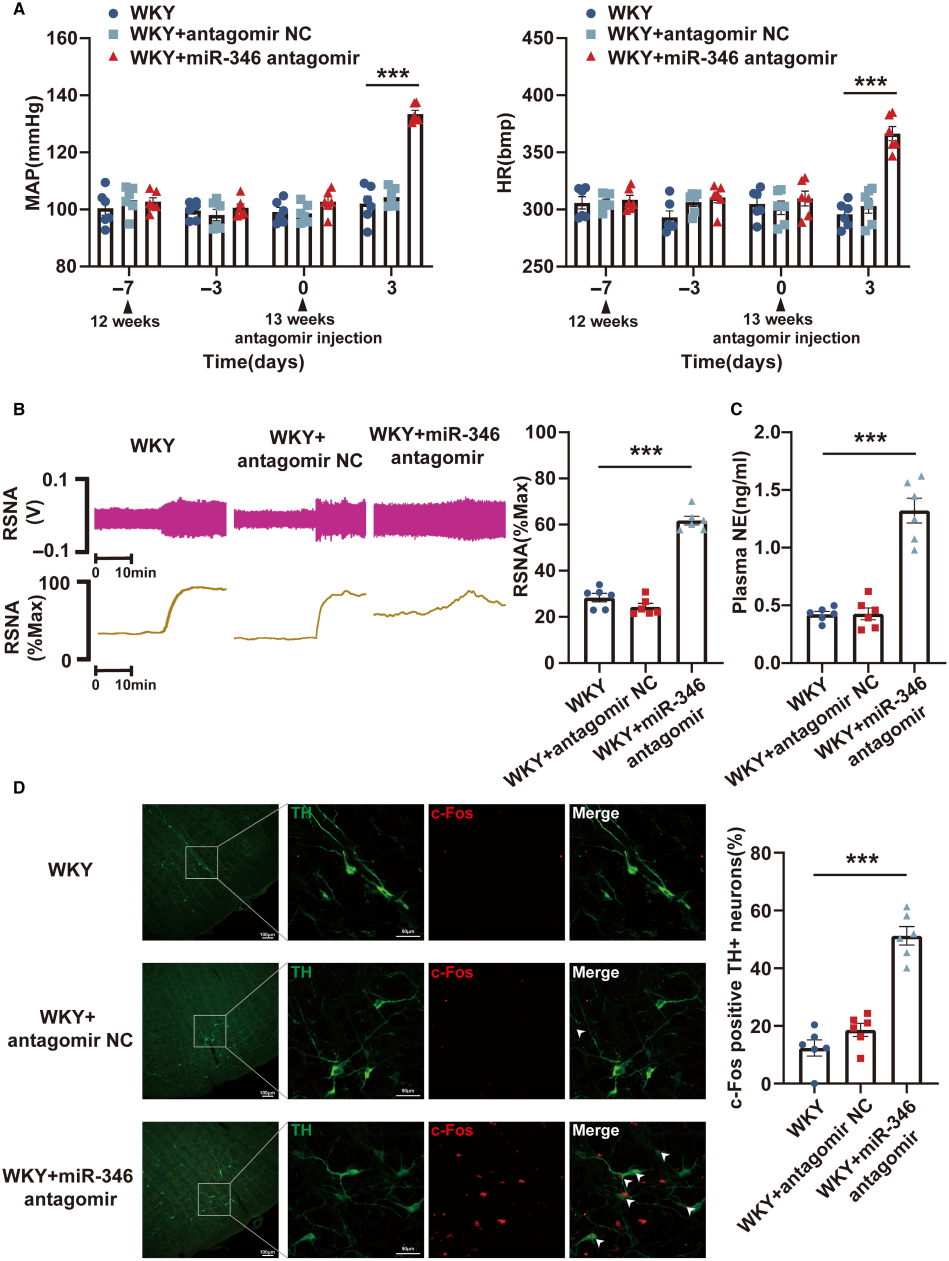
miR‐346 downregulation in RVLM led to hypertension in WKY rats. **A**, Radiotelemetry was used to evaluate the changes in MAP and HR in WKY rats following downregulation of miR‐346. **B** and **C**, RSNA recording and ELISA were used to reveal the effect of miR‐346 downregulation on sympathetic outflow in WKY rats. **D**, Immunostaining was used to assess the excitability of RVLM neurons in WKY rats after downregulation of miR‐346. N=6 rats per group (**A** through **D**). ***P<0.001. HR indicates heart rate; MAP, mean arterial pressure; NC, negative control; NE, norepinephrine; RSNA, renal sympathetic nerve activity; RVLM, rostral ventrolateral medulla; TH, tyrosine hydroxylase; and WKY, Wistar Kyoto.

Loss‐of‐function experiments were performed in WKY rats by administering miR‐193b‐3p antagomir, miR‐346 antagomir, and miR‐322‐3p antagomir to evaluate their effects on hypertension. Bilateral microinjections of the 3 antagomirs were performed into the RVLM of 13‐week‐old WKY rats. Following 72 hours of microinjection, the knockdown efficiency was assessed using reverse transcription‐qPCR (Figure S7B). Through the radiotelemetry, RSNA recording, ELISA, and immunofluorescence experiments, the levels of MAP, HR, RSNA, and plasma norepinephrine and the proportion of c‐Fos‐positive TH+RVLM neurons in WKY rats were found to be observably increased by miR‐193b‐3p (Figure 4A through 4D) and miR‐346 (Figure 5A through 5D) silencing. miR‐322‐3p knockdown did not alter the MAP and HR of WKY rats (Figure S9). In conclusion, these results suggested that inhibiting the expression of miR‐193b‐3p and miR‐346 in the RVLM of WKY rats could lead to neuronal excitation and sympathetic nerve activation, resulting in increased BP.

### Silencing the Expression of miR‐193b‐3p and miR‐346 in RVLM Increased MAP and HR in SD Rats

**Figure 6:**
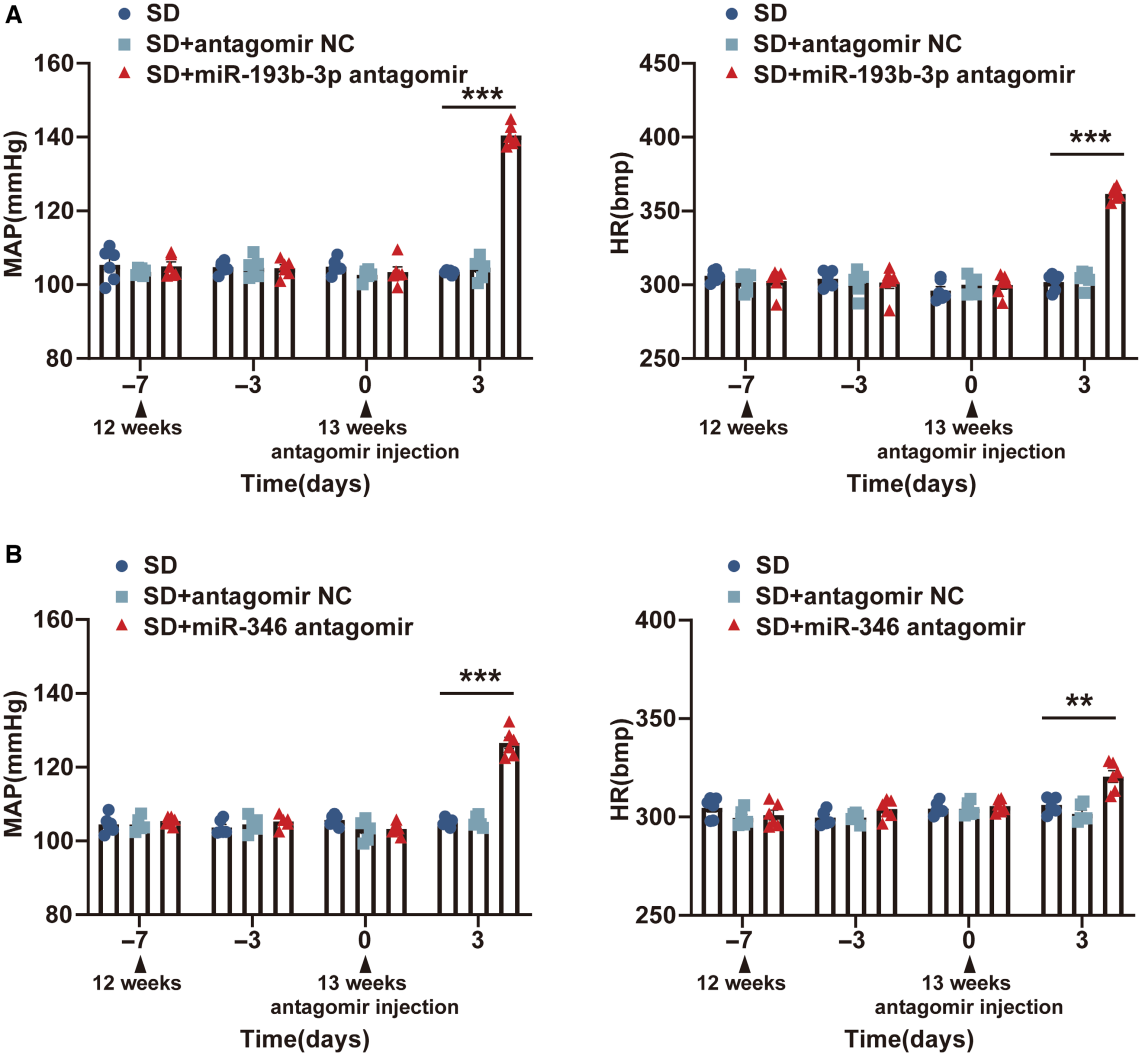
Inhibition of miR‐193b‐3p and miR‐346 in RVLM of SD rats was associated with BP elevation. **A** and **B**, Radiotelemetry was used to monitor the effects of miR‐193b‐3p and miR‐346 downregulation on MAP and HR in SD rats. N=6 rats per group (**A** and **B**). **P<0.01. ***P<0.001. BP indicates blood pressure; HR, heart rate; MAP, mean arterial pressure; NC, negative control; RVLM, rostral ventrolateral medulla; and SD, Sprague‐Dawley.

Reverse transcription‐qPCR assay revealed a significant downregulation in the expression levels of miR‐193b‐3p, miR‐346, and miR‐322‐3p in the RVLM of SHRs compared with WKY and SD rats (Figure S10). The expression differences of these miRNAs in other cardiovascular centers (nucleus tractus solitarius, caudal ventrolateral medulla, and paraventricular nucleus) of the 3 groups of rats were not significant (Figure S11). For further confirmation of their functional roles in hypertension in SD rats, the RVLMs of the 13‐week‐old SD rats were microinjected with miR‐193b‐3p antagomir, miR‐346 antagomir, and miR‐322‐3p antagomir. reverse transcription‐qPCR was used to assess the knockdown efficiency after 72 hours of microinjection (Figure S7C). The findings obtained through radiotelemetry demonstrated a notable enhancement in the MAP and HR levels of SD rats following the silencing of miR‐193b‐3p (Figure 6A) and miR‐346 (Figure 6B). The MAP and HR levels remained unaffected in SD rats after the silencing of miR‐322‐3p (Figure S12). These results provided additional evidence for the involvement of miR‐193b‐3p and miR‐346 in the regulation of hypertension.

### Functional Enrichment Analysis Identified a Strong Correlation Between miR‐193b‐3p and miR‐346 and Hypertension

By combining the analysis from TargetScan and miRanda software, the potential target genes of miR‐193b‐3p and miR‐346 were predicted. As displayed in Tables S4 and S5, a total of 949 and 1043 target genes were predicted for miR‐193b‐3p and miR‐346, respectively. The GO and KEGG survey on the target genes of miR‐193b‐3p showed significant enrichment of 72 GO terms (Table S6 and Figure S13A) and 30 KEGG pathways (Table S7 and Figure S13B). The analysis uncovered several terms and pathways involved in the neurogenic pathogenesis of hypertension, such as negative regulation of intrinsic apoptotic signaling pathway (GO: 2001243), Mitogen‐activated protein kinase signaling pathway, calcium signaling pathway, and PI3K‐Akt signaling pathway. Similarly, GO and KEGG analyses were conducted on the target genes of miR‐346, revealing significant enrichment of 51 GO terms (Table S8 and Figure S13C) and 35 KEGG pathways (Table S9 and Figure S13D). The findings, such as regulation of autophagy of mitochondrion (GO: 1903146), Mammalian target of rapamycin (mTOR) signaling pathway, cAMP signaling pathway, and calcium signaling pathway, were also reflected in the neurogenic pathogenesis of hypertension. Consequently, the target gene networks of miR‐193b‐3p and miR‐346 can participate in the central nervous regulation of hypertension through various aspects.

### miR‐193b‐3p Directly Targeted *Arhgef9*

**Figure 7:**
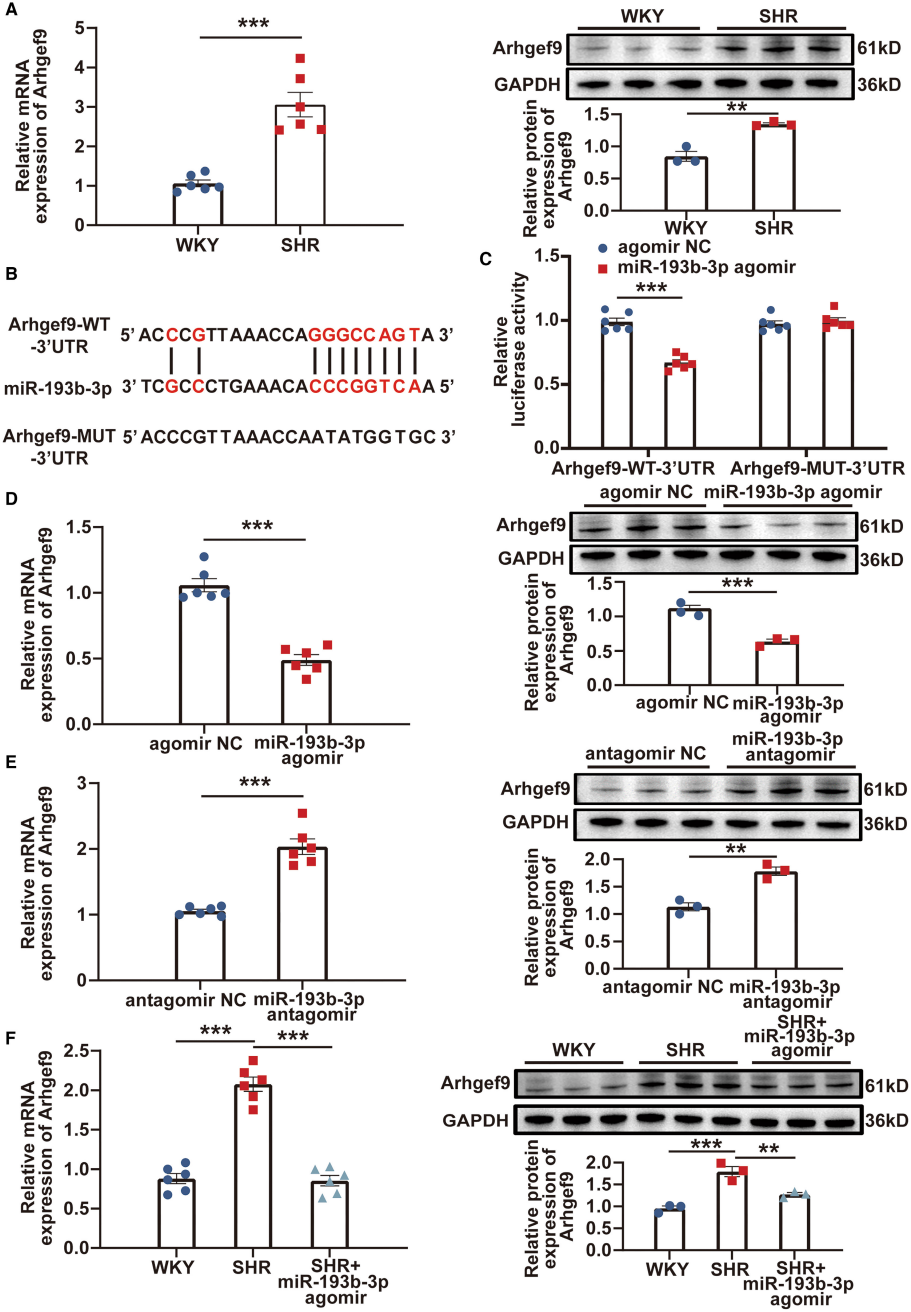
*Arhgef9* was a direct target of miR‐193b‐3p. **A**, *Arhgef9* expression in RVLM of SHR and WKY rats was assessed using RT‐qPCR and Western blot. **B**, Schematic of *Arhgef9*‐WT‐3′ UTR and *Arhgef9*‐MUT‐3′ UTR binding site for miR‐193b‐3p is shown. **C**, Dual‐luciferase reporter assay was performed to validate the direct interaction between miR‐193b‐3p and *Arhgef9*. **D** and **E**, *Arhgef9* expression in RVLM primary neurons was evaluated through RT‐qPCR and Western blot following overexpression or knockdown of miR‐193b‐3p. **F**, After miR‐193b‐3p was overexpressed in the RVLM of SHRs, the expression of *Arhgef9* was determined using RT‐qPCR and Western blot. N=3 to 6 rats per group (**A** and **F**). N=3 to 6 of independent cell culture preparations (**C** through **E**). **P<0.01. ***P<0.001. MUT indicates mutant; *Arhgef9* indicates Cdc42 guanine nucleotide exchange factor 9; BAX, BCL2‐associated X; BCL2, B cell lymphoma 2; GAPDH, Glyceraldehyde‐3‐phosphate dehydrogenase; NC, negative control; RT‐qPCR, reverse transcription‐quantitative polymerase chain reaction; RVLM, rostral ventrolateral medulla; SHR, spontaneously hypertensive rat; UTR, untranslated region; WKY, Wistar Kyoto; and WT, wild‐type.

Apoptosis of neuron cells in RVLM leads to elevated sympathetic nerve activity, thereby playing a role in the progression of hypertension.^18^, ^20^, ^32^ In the present study, Western blot analysis demonstrated a significant decrease in the expression of antiapoptotic B cell lymphoma 2 (BCL2) protein, along with a notable increase in the expression levels of proapoptotic BCL2‐associated X (BAX) and cleaved Caspase 3 proteins, in the RVLM of SHRs (Figure S14). *Arhgef9* has been identified as a factor that promotes apoptosis.^33^, ^34^ In SHRs, the expression levels of *Arhgef9* in RVLM were evaluated through reverse transcription‐qPCR and Western blot, revealing a substantial increase in *Arhgef9* mRNA and protein expression levels (Figure 7A). Moreover, reverse transcription‐qPCR and Western blot were performed to investigate the mRNA and protein expression levels of *Arhgef9* in microglia, astrocytes, and neurons isolated from the RVLM tissues of 13‐week‐old SHRs and WKY rats. The results uncovered that the expression of *Arhgef9* were significantly higher in neurons of the SHRs than that in WKY rats (Figure S15A). No significant differences were observed in the expression levels of *Arhgef9* between the 2 groups in RVLM microglia and astrocytes (Figure S15B and S15C). By using TargetScan and miRanda tools, miR‐193b‐3p was discovered to have potential binding sites within the *Arhgef9* gene (Figure 7B). B104 cells were cotransfected with miR‐193b‐3p agomir or agomir NC, along with either the *Arhgef9*‐wild‐type or *Arhgef9*‐mutant‐3′ UTR reporter plasmid, for dual‐luciferase reporter assay. Upon overexpression of miR‐193b‐3p, a notable decrease in luciferase activity was observed in the *Arhgef9*‐wild‐type‐3′ UTR reporter, whereas the luciferase activity of the Arhgef9‐mutant‐3′ UTR reporter remained unchanged (Figure 7C). By using miR‐193b‐3p agomir and miR‐193b‐3p antagomir, successful miR‐193b‐3p overexpression and knockdown were achieved in RVLM primary neurons. The efficiencies of overexpression and knockdown were determined by reverse transcription‐qPCR analysis (Figure S7D). The results depicted in Figure 7D demonstrate a significant reduction in the mRNA and protein levels of *Arhgef9* upon miR‐193b‐3p overexpression. Conversely, miR‐193b‐3p knockdown led to a considerable increase in *Arhgef9* expression (Figure 7E). Additionally, in an in vivo study, the expression of *Arhgef9* in the RVLM of SHR+agomir rats significantly reduced compared with that in the SHRs (Figure 7F). The results strongly supported the notion that miR‐193b‐3p targets *Arhgef9*, thereby providing robust evidence of their functional association.

### Increasing the Expression of miR‐193b‐3p Attenuated Neuronal Apoptosis by Targeting *Arhgef9* in the RVLM of SHRs

**Figure 8:**
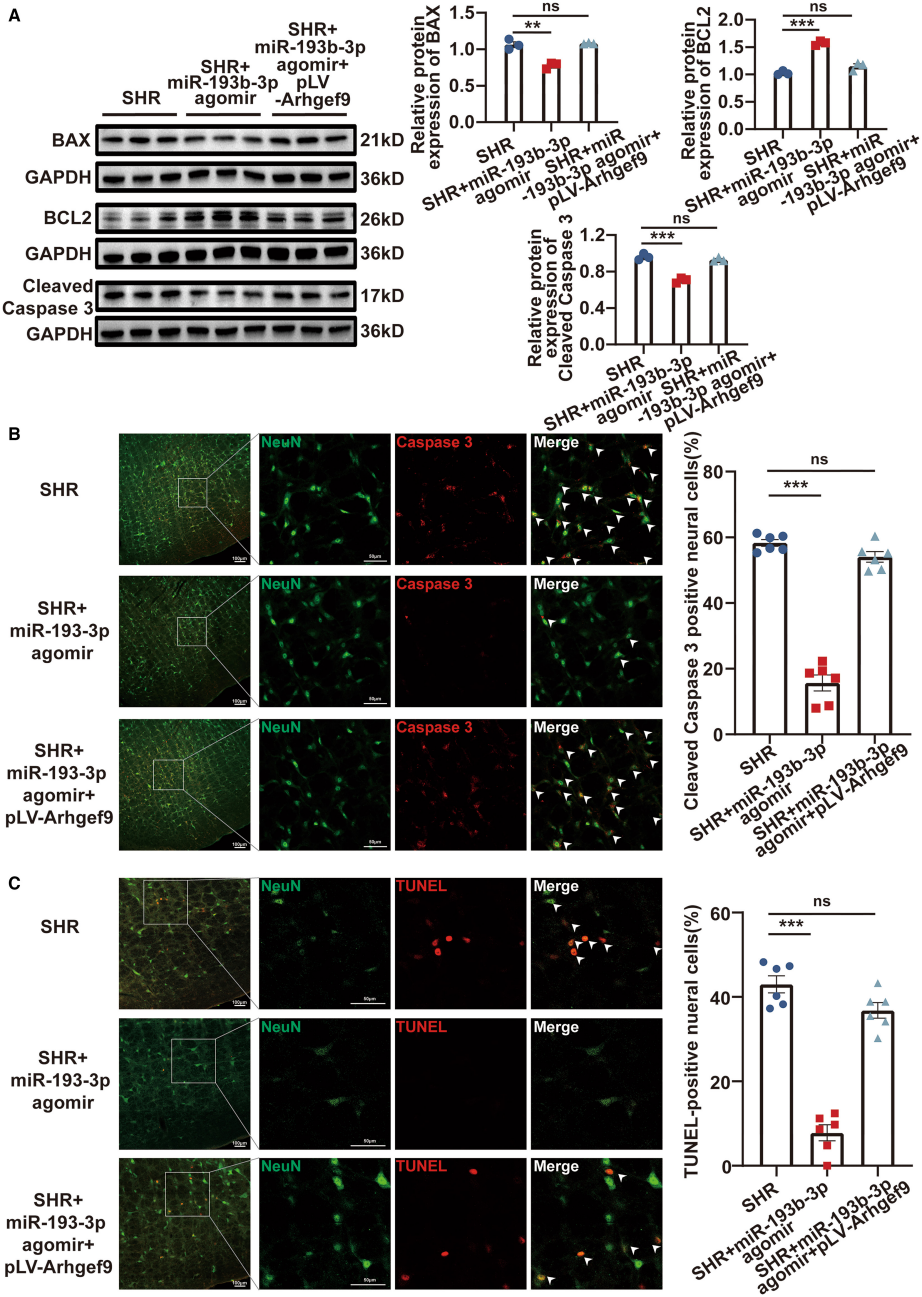
miR‐193b‐3p repressed RVLM neuronal apoptosis of SHRs by binding to *Arhgef9*. **A** through **C**, Following the microinjection of miR‐193b‐3p agomir and pLV‐*Arhgef9* plasmid into the RVLM of SHRs, BCL2, BAX, and cleaved Caspase 3 protein levels were assessed using Western blot; cleaved Caspase 3 protein level was analyzed through immunofluorescence staining; and neuronal apoptotic level was determined by TUNEL assay. N=3 to 6 rats per group (**A** through **C**). **P<0.01. ***P<0.001. *Arhgef9* indicates Cdc42 guanine nucleotide exchange factor 9; BAX, BCL2‐associated X; BCL2, B cell lymphoma 2; GAPDH, Glyceraldehyde‐3‐phosphate dehydrogenase; ns, nonsignificant RVLM, rostral ventrolateral medulla; SHR, spontaneously hypertensive rat; and TUNEL, TdT‐mediated dUTP‐biotin nick end‐labelinge.

By considering the binding relationship between miR‐193b‐3p and *Arhgef9*, the effect of miR‐193b‐3p/*Arhgef9* on neuronal apoptosis was elucidated in vivo. The RVLM of SHRs was bilaterally microinjected with pLV‐*Arhgef9* plasmid at 11 and 12 weeks of age. The 13‐week‐old rats were then administered with bilateral RVLM microinjections of miR‐193b‐3p agomir. Reverse transcription‐qPCR was performed to evaluate the expression of *Arhgef9* in the groups, as shown in Figure S7E. Western blot was performed to assess the expression of BCL2, BAX, and cleaved Caspase 3 in RVLM of the experimental groups. As shown in Figure 8A, overexpression of miR‐193b‐3p resulted in a significant upregulation of BCL2 expression and downregulation of BAX and cleaved Caspase 3 levels in the RVLM of SHRs. However, this effect was diminished when *Arhgef9* overexpression was introduced. Immunofluorescence staining was used to further examine the expression of cleaved Caspase 3 protein. miR‐193b‐3p overexpression in the RVLM notably reduced the percentage of cleaved Caspase 3‐positive neural cells in SHRs, and this decrease was reversed upon upregulation of *Arhgef9* (Figure 8B). TUNEL assay was performed to test the level of RVLM neuronal apoptosis. The number of TUNEL‐positive RVLM neural cells was decreased by miR‐193b‐3p agomir in SHRs, whereas the overexpression of *Arhgef9* hindered this influence (Figure 8C). Collectively, the data strongly suggested that miR‐193b‐3p acts as an inhibitor of RVLM neuronal apoptosis in SHRs by directly targeting *Arhgef9*.

### miR‐193b‐3p Downregulation Led to an Increase in Apoptosis by Binding to *Arhgef9* in RVLM Primary Neurons

**Figure 9:**
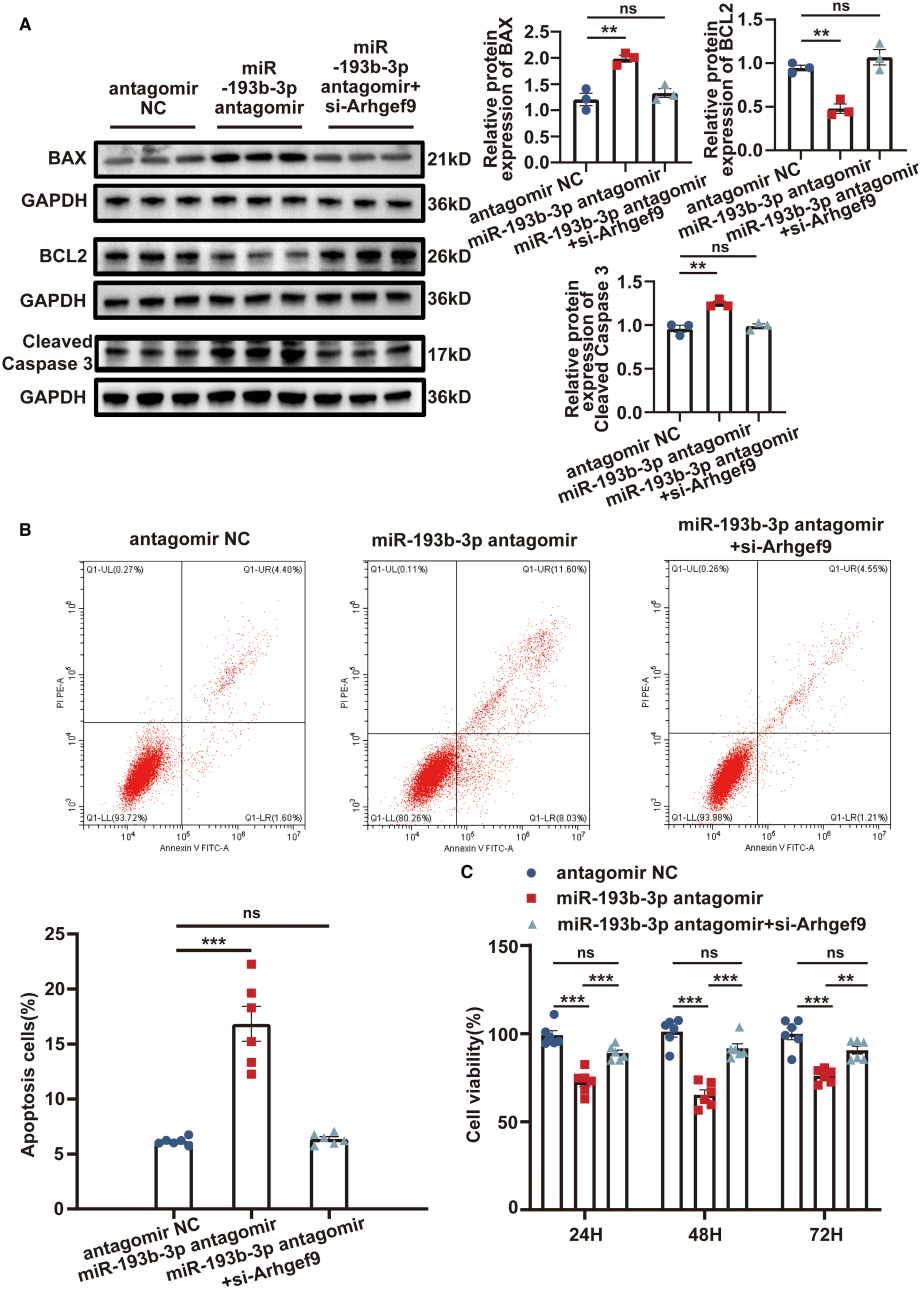
miR‐193b‐3p attenuated apoptosis in RVLM primary neurons via targeting *Arhgef9*. RVLM primary neurons were transfected with antagomir NC, miR‐193b‐3p antagomir, or miR‐193b‐3p antagomir + pLV‐Arhgef9‐shRNA for 48 hours. **A**, Western blot was performed to investigate the protein levels of BCL2, BAX, and cleaved Caspase 3. **B**, Flow cytometry was used to detect cell apoptosis. **C**, CCK‐8 was used to evaluate cell viability. N=3 to 6 of independent cell culture preparations (**A through C**). **P<0.01. ***P<0.001. *Arhgef9* indicates Cdc42 guanine nucleotide exchange factor 9; BAX, BCL2‐associated X; BCL2, B cell lymphoma 2; CCK, Cell Counting Kit‐8; FITC‐A, fluorescein isothiocyanate‐area; LL, lower left; UL, upper left; LR, lower right; NC, negative control; ns, nonsignificant; PIPE‐A, Propidium Iodide‐area; Q1, quadrant 1; RVLM, rostral ventrolateral medulla; GAPDH, Glyceraldehyde‐3‐phosphate dehydrogenase; shRNA, short hairpin RNA and UR, upper right.

In vitro experiments were performed to further provide insights into the role of miR‐193b‐3p/*Arhgef9* in neuronal apoptosis. miR‐193b‐3p antagomir and pLV‐*Arhgef9*‐shRNA were transfected into the RVLM primary neurons. After 48 hours of transfection, the expression of *Arhgef9* in the groups was assessed through reverse transcription‐qPCR (Figure S7F). Western blot indicated that miR‐193b‐3p silencing markedly reduced BCL2 expression and increased BAX and cleaved Caspase 3 levels in RVLM primary neurons, and *Arhgef9* downregulation attenuated this effect (Figure 9A). Flow cytometry and Cell Counting Kit‐8 assays demonstrated that downregulation of miR‐193b‐3p induced cell apoptosis (Figure 9B) and inhibited cell viability (Figure 9C). Notably, *Arhgef9* knockdown abrogated these effects. These results demonstrated that miR‐193b‐3p exerts an antiapoptotic effect on RVLM primary neurons by targeting *Arhgef9*.

### miR‐193b‐3p Absorbed *Arhgef9* in RVLM to Improve Hypertensive Symptoms of SHRs

**Figure 10:**
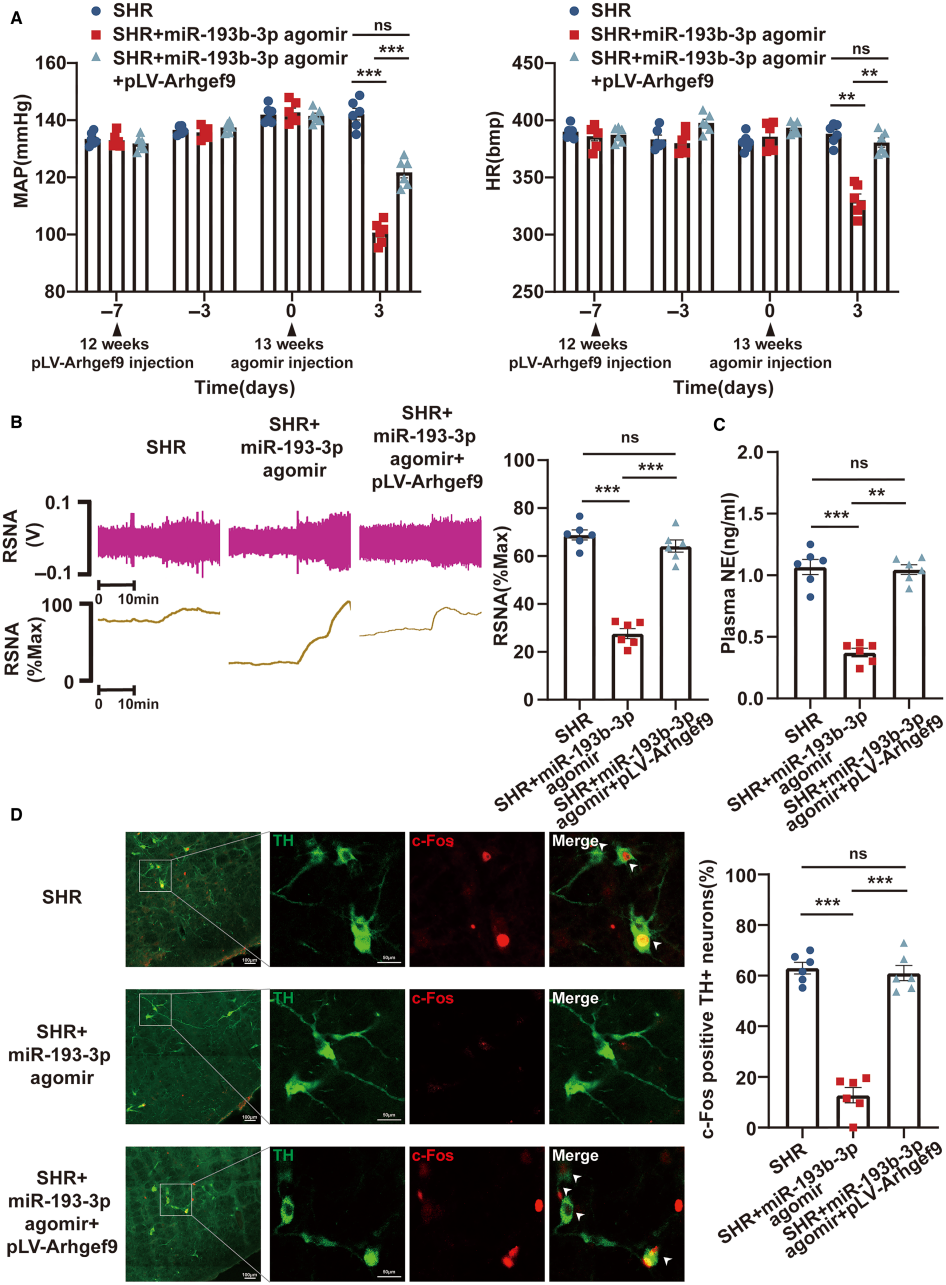
miR‐193b‐3p binding to *Arhgef9* in RVLM alleviated hypertensive symptoms in SHRs. **A** through **D**, After miR‐193b‐3p agomir and pLV‐Arhgef9 plasmid were microinjected into the RVLM of SHRs, MAP and HR were measured by radiotelemetry, RSNA was detected by RSNA recording, plasma NE was determined by ELISA, and the proportion of c‐Fos‐positive TH+ RVLM neurons was examined by immunofluorescence. N=6 rats per group (**A** through **D**). **P<0.01. ***P<0.001. *Arhgef9* indicates Cdc42 guanine nucleotide exchange factor 9; HR, heart rate; MAP, mean arterial pressure; NE, norepinephrine; ns, nonsignificant; RSNA, renal sympathetic nerve activity; RVLM, rostral ventrolateral medulla; SHR, spontaneously hypertensive rat; and TH, tyrosine hydroxylase.

Whether the miR‐193b‐3p/*Arhgef9*/neuronal apoptosis axis in RVLM is involved in the neurogenic pathogenesis of hypertension was unveiled. As illustrated in Figure 10A through 10D, the results obtained from radiotelemetry, RSNA recording, ELISA, and immunofluorescence experiments demonstrated that overexpression of miR‐193b‐3p led to observable reduction in the levels of MAP, HR, RSNA, and plasma norepinephrine and the proportion of c‐Fos‐positive TH+RVLM neurons in SHRs. However, these effects were reversed upon upregulation of *Arhgef9*. These results implied that miR‐193b‐3p suppresses neuronal apoptosis by targeting *Arhgef9* in RVLM to lessen neuronal excitability and sympathetic outflow, thereby opposing the progression of hypertension.

## Discussion

The neuronal network in RVLM plays a critical role in the regulation of sympathetic activity by receiving and integrating various internal and external inputs, making it a pivotal node in the control of BP.^7^, ^8^, ^35^ Exploring the gene expression changes in RVLM is crucial to understand the neurogenic pathogenesis of hypertension. As remarkable posttranscriptional regulators, miRNAs are pivotal in controlling gene expression with fine‐tuned precision and acknowledged as key players in modulating various physiological and pathological processes.^36^ Given their ability to influence numerous genes and pathways, targeting miRNAs holds great potential for therapeutic interventions in various diseases. The RVLM of a particular rat model with hypertension induced through electric foot‐shocks and noises exhibited a notable increase in the expression levels of miR‐335 and miR‐674‐3p, as reported in the authors' earlier study.^18^ Additional research and attention should be paid to the regulating functions of RVLM miRNAs on hypertension and the underlying mechanisms. This study aimed to continue the exploration of RVLM miRNAs and their involvement in the development of hypertension, unraveling their underlying regulatory mechanisms in the modulation of sympathetic drive and BP. This work could add to the existing literature and provide more references for the development of miRNA‐based therapies for hypertension.

The analysis of expression changes is of utmost importance in discerning the biological variations between disease and health, providing valuable information for the identification of potential diagnostic biomarkers and therapeutic targets. The expression levels of miR‐193b‐3p, miR‐346, and miR‐322‐3p in the RVLM of SHRs were significantly downregulated compared with those of the normal control WKY rats and normotensive SD rats. They exhibited similar expression levels in the RVLM of WKY and SD rats. In addition, no differences were noted in their expression levels in other cardiovascular centers (nucleus tractus solitarius, caudal ventrolateral medulla, and paraventricular nucleus). The downregulation of these 3 miRNAs in RVLM is widely observed in different models, suggesting their potential crucial role in BP regulation. Then, gain‐of‐function and loss‐of‐function strategies were used to examine whether they played roles in the neurogenic pathogenesis of hypertension. Administration of agomirs for miR‐193b‐3p and miR‐346 in the RVLM of SHRs led to a decrease in MAP, HR, RSNA, plasma norepinephrine, and the ratio of c‐Fos‐positive TH+RVLM neurons. Conversely, the application of specific antagomirs targeting miR‐193b‐3p and miR‐346 in WKY and SD rats resulted in an increase in MAP, HR, RSNA, plasma norepinephrine, and the ratio of c‐Fos‐positive TH+RVLM neurons. Overexpression or knockdown of miR‐322‐3p did not exhibit the abovementioned effects, suggesting that the differential expression of miR‐322‐3p may be a mere response without functional consequences. Hence, the changes in miR‐193b‐3p and miR‐346 expression in RVLM are pivotal in the central nervous system's regulation of hypertension, indicating their noteworthy clinical translational significance.

miR‐193b‐3p, a highly conserved miRNA across species (hsa‐miR‐193b‐3p, mmu‐miR‐193b‐3p, and rno‐miR‐193b‐3p), is implicated in the regulation of various disease‐related processes. Lai et al demonstrated that the delivery of miR‐193b‐3p effectively mitigated neuroinflammation in the early stages of brain injury after subarachnoid hemorrhage in mice.^37^ Jing et al showed that miR‐193b‐3p played a role in inflammation inhibition in allergic rhinitis through the targeting of ETS proto‐oncogene 1 and subsequent regulation of Toll like receptor 4 expression.^38^ Scientific evidence strongly supports the involvement of miR‐193b‐3p in the regulation of chondrogenesis and chondrocyte metabolism through the direct targeting of Histone deacetylase 3.^39^ miR‐193b‐3p upregulation alleviated neuronal apoptosis by targeting Calmodulin in vascular dementia model rats (2‐vessel occlusion rats).^40^ miR‐193b‐3p acts as a key player in the pathogenesis of cerebral ischemia–reperfusion injury, exerting neuroprotection through its ability to suppress neuronal apoptosis.^41^, ^42^ The current study unveiled a previously unreported phenomenon of diminished miR‐193b‐3p expression in the RVLM of SHRs. Elevated levels of miR‐193b‐3p in RVLM were found to suppress neuronal excitability and sympathetic outflow, leading to improved BP control. Across different species, including humans, rats, and mice, the presence of miR‐346 (hsa‐miR‐346, mmu‐miR‐346‐5p, and rno‐miR‐346) is highly conserved. Dysregulated expression of miR‐346 has been associated with numerous physiological and pathological processes. Zhang et al reported that the administration of miR‐346 resulted in the downregulation of SMAD family member 3/4 expression in renal tissue, ultimately leading to the amelioration of renal function and improvement in glomerular histology in diabetic nephropathy mice.^43^ Moreover, miR‐346 upregulation stimulates liver cancer cell proliferation, migration, and invasion.^44^ The colon of patients with primary sclerosing cholangitis exhibited an upregulation of miR‐346, potentially leading to the disturbance of the vitamin D receptor and Tumor necrosis factor‐α signaling pathway.^45^ The interaction between circ0083429 and miR‐346 can regulate osteoarthritis progression.^46^ For the first time, the present study reported a decrease in miR‐346 expression in the RVLM of SHRs when compared with healthy controls. The enhancement of miR‐346 expression in RVLM can effectively inhibit neuronal excitability, sympathetic outflow, and lower BP, offering improvements in hypertension symptoms.

The mature form of miRNA binds to its target mRNAs by recognizing complementary sequences located in the 3′ UTR through the seed region, which usually encompasses positions 2 to 8 in the miRNA molecule.^47^, ^48^, ^49^, ^50^ Following this principle, TargetScan and miRanda tools were used to identify a total of 949 potential target genes for miR‐193b‐3p and 1043 for miR‐346. GO and KEGG enrichments pertaining to the interactions between miR‐193b‐3p and mRNAs, as well as miR‐346 and mRNAs, were subsequently examined. miR‐193b‐3p and miR‐346 were found to be involved in the neurogenic pathogenesis of hypertension from different aspects, such as negative regulation of intrinsic apoptotic signaling pathway (GO: 2001243) and mTOR signaling pathway. Studies suggested that neuronal apoptosis in RVLM promotes neuronal excitability and sympathetic outflow, thereby causing an increase in BP.^18^, ^20^, ^32^ Through the analysis of BCL2, BAX, and cleaved Caspase 3 expression, evident apoptosis was detected in the RVLM neurons of SHRs. The *Arhgef9* gene encodes for ARHGEF9, a cell division cycle 42 (CDC42) guanine nucleotide exchange factor that modulates CDC42 and other genes by transitioning between its active (guanosine triphosphate‐bound) and inactive (guanosine diphosphate‐bound) states.^51^ It has been recognized as a contributor to apoptosis.^33^, ^34^ The present study revealed an upregulation of *Arhgef9* expression in the RVLM of SHRs, which displayed a negative correlation with miR‐193b‐3p expression. The results obtained from dual‐luciferase reporter assays, gain‐of‐function studies, and loss‐of‐function studies provided compelling evidence that *Arhgef9* is a direct target of miR‐193b‐3p. Overexpression of *Arhgef9* negated the protective effect of miR‐193b‐3p agomir against RVLM neuronal apoptosis in SHRs. *Arhgef9* knockdown counteracted the proapoptotic effect of miR‐193b‐3p antagomir in the RVLM primary neurons. Most notably, this research substantiated the regulatory role of miR‐193b‐3p in inhibiting RVLM neuronal apoptosis through targeted suppression of *Arhgef9*, resulting in a decrease in neuronal excitability and sympathetic outflow and consequently impeding the development of hypertension. miR‐193b‐3p suppressed the progression of hypertension by decreasing RVLM neuronal apoptosis, and the underlying mechanism was that miR‐193b‐3p negatively regulated *Arhgef9* expression.

This study has a limitation. Previous research indicated that sex difference was connected to the pathogenesis of hypertension.^52^ Female rats were not used in this study.

## Conclusions

Overall, these findings provide evidence that the decrease in miR‐193b‐3p and miR‐346 expression in RVLM contributes to elevated neuronal excitability, sympathetic nerve activity, and BP in rats. Mechanistically, the miR‐193b‐3p/*Arhgef9*/apoptosis axis emerges as a key pathway linked to the neurogenic pathogenesis of hypertension. The innovative nature of this study lies in its significant advancements in understanding the functions of noncoding RNAs, specifically miR‐193b‐3p and miR‐346, in the central neural regulation of hypertension, with profound implications for clinical practice.

## Sources of Funding

This work was supported by the National Natural Science Foundation of China to S.Z. (32200929) and D.D. (32071111, 31871151, 31571171, 32371165), the Natural Science Foundation of Shandong Province to D.D. (ZR202112030301), the Special Project of Science and Technology Plan of Shaoxing Bureau of Science and Technology to D.D. (2020B33004), and the Zhejiang Chinese Medical University Research Funding to S.Z. (111100E018/002/001/096).

## Disclosures

None.

## Supporting information

Data S1
