# Supplementary material for: miR‐193b‐3p and miR‐346 Exert Antihypertensive Effects in the Rostral Ventrolateral Medulla
Source: J Am Heart Assoc. 2024 Jun 27;13(13):e034965. doi: 10.1161/JAHA.124.034965 (PMC11255704; doi:10.1161/JAHA.124.034965)
Supplement: Supplementary file 1 — Data S1 [file JAH3-13-e034965-s001.pdf]

SUPPLEMENTAL MATERIAL

Table S1 Primer pairs used in RT-qPCR

Name	Primer sequence (5'-3')	Annealing temperature (℃)	The size of production (bp)
miR-193b-3p	F: AAGCGGAAACTGGCCCACAAA	60 ℃	65bp
	R: ATCCAGTGCAGGGTCCGAGG		
miR-346	F: AACACGCTGTCTGCCTGAGTG	60 ℃	65bp
	R: ATCCAGTGCAGGGTCCGAGG		
miR-322-3p	F: AGGCGCAAACATGAAGCGCT	60 ℃	65bp
	R: ATCCAGTGCAGGGTCCGAGG		
miR-582-3p	F: AAGCGCCTAACCTGTTGAACAAC	60 ℃	65bp
	R: ATCCAGTGCAGGGTCCGAGG		
miR-488-3p	F: AAGCGACCTTGAAAGGCTGTTTC	60 ℃	65bp
	R: ATCCAGTGCAGGGTCCGAGG		
Arhgef9	F: ATCACCCCTCCAATTCTGCC	60 ℃	136bp
	R: AGGTGCTGTCCTCCAAACAC		
U6	F:GCTTCGGCAGCACATATACTAAAAT	60 ℃	94bp
	R: CGCTTCACGAATTTGCGTGTCAT		
GAPDH	F: GTCGGTGTGAACGGATTTG	60 ℃	181bp
	R: TCCCATTCTCAGCCTTGAC		

Table S2 AgomiRs, antagomiRs, and Arhgef9 siRNA used in this study.

Acession No.	Sequence (5'-3')
miR-193b-3p agomir	AACUGGCCCACAAAGUCCCGCU
	AGCGGGACUUUGUGGGCCAGUU
miR-193b-3p antagomir	AGCGGGACUUUGUGGGCCAGUU
miR-346 agomir	TGTCTGCCTGAGTGCCTGCCTCT
	UGUCUGCCUGAGUGCCUGCCUCU
miR-346 antagomir	ACAGACGGACUCACGGACGGAGA
miR-322 agomir	AAACATGAAGCGCTGCAACAC
	GUGUUGCAGCGCUUCAUGUUU
miR-322 antagomir	UUUGUACUUCGCGACGUUGUG
agomir NC	UUUGUACUACACAAAAGUACUG
	CAGUACUUUUGUGUAGUACAAA
antagomir NC	CAGUACUUUUGUGUAGUACAAA
Arhgef9 siRNA	TTCTCTGAAAGCTCTAAGC

Table S3 The important pairwise post hoc *P* values

Figure 1D

		P value
Relative expression of miR-193b-3p	RVLM versus Heart	<0.0001
Relative expression of miR-346	RVLM versus Heart	0.0001
Relative expression of miR-322-3p	RVLM versus Heart	<0.0001

Figure 2A

		P value
Mean arterial pressure (MAP)	SHR+miR-193b-3p agomir versus SHR	<0.0001
Heart rate (HR)	SHR+miR-193b-3p agomir versus SHR	<0.0001

Figure 2B

		P value
Renal sympathetic nerve activity (RSNA)	SHR+miR-193b-3p agomir versus SHR	<0.0001

Figure 2C

		P value
Plasma NE	SHR+miR-193b-3p agomir versus SHR	0.0022

Figure 2D

		P value
c-Fos positive TH+ neurons	SHR+miR-193b-3p agomir versus SHR	0.0009

Figure 3A

		P value
Mean arterial pressure (MAP)	SHR+miR-346 agomir versus SHR	<0.0001
Heart rate (HR)	SHR+miR-346 agomir versus SHR	<0.0001

Figure 3B

		P value
Renal sympathetic nerve activity (RSNA)	SHR+miR-346 agomir versus SHR	<0.0001

Figure 3C

		P value
Plasma NE	SHR+miR-346 agomir versus SHR	<0.0001

Figure 3D

		P value
c-Fos positive TH+ neurons	SHR+miR-346 agomir versus SHR	0.0002

Figure 4A

		P value
Mean arterial pressure (MAP)	WKY+miR-193-3p antagomir versus WKY	<0.0001
Heart rate (HR)	WKY+miR-193-3p antagomir versus WKY	<0.0001

Figure 4B

		P value
Renal sympathetic nerve activity (RSNA)	WKY+miR-193-3p antagomir versus WKY	<0.0001

Figure 4C

		P value
Plasma NE	WKY+miR-193-3p antagomir versus WKY	<0.0001

Figure 4D

		P value
c-Fos positive TH+ neurons	WKY+miR-193-3p antagomir versus WKY	<0.0001

Figure 5A

		P value
Mean arterial pressure (MAP)	WKY+miR-346 antagomir versus WKY	<0.0001
Heart rate (HR)	WKY+miR-346 antagomir versus WKY	<0.0001

Figure 5B

		P value
Renal sympathetic nerve activity (RSNA)	WKY+miR-346 antagomir versus WKY	<0.0001

Figure 5C

		P value

Plasma NE	WKY+miR-346 antagomir versus WKY	<0.0001
**Figure 5D**		
		P value
c-Fos positive TH+ neurons	WKY+miR-346 antagomir versus WKY	<0.0001
**Figure 6A**		
		P value
Mean arterial pressure (MAP)	SD+miR-193-3p antagomir versus SD	<0.0001
Heart rate (HR)	SD+miR-193-3p antagomir versus SD	<0.0001
**Figure 6B**		
		P value
Mean arterial pressure (MAP)	SD+miR-346 antagomir versus SD	<0.0001
Heart rate (HR)	SD+miR-346 antagomir versus SD	<0.0001
**Figure 7F**		
		P value
Relative mRNA expression of Arhgef9	WKY versus SHR	<0.0001
	SHR+miR-193b-3p agomir versus SHR	<0.0001
Relative protein expression of Arhgef9	WKY versus SHR	0.0022
	SHR+miR-193b-3p agomir versus SHR	0.0009
**Figure 8A**		
		P value
Relative protein expression of BAX	SHR+miR-193b-3p agomir versus SHR	0.0006
	SHR+miR-193b-3p agomir+pLV-Arhgef9 versus SHR	0.8713
Relative protein expression of BCL2	SHR+miR-193b-3p agomir versus SHR	<0.0001
	SHR+miR-193b-3p agomir+pLV-Arhgef9 versus SHR	0.0544
Relative protein expression of Cleaved Caspase-3	SHR+miR-193b-3p agomir versus SHR	<0.0001
	SHR+miR-193b-3p agomir+pLV-Arhgef9 versus SHR	0.3831
**Figure 8B**		
		P value
Cleaved Caspase-3 positive neural cells	SHR+miR-193b-3p agomir versus SHR	<0.0001
	SHR+miR-193b-3p agomir+pLV-Arhgef9 versus SHR	0.1387
**Figure 8C**		
		P value
TUNEL-positive neural cells	SHR+miR-193b-3p agomir versus SHR	<0.0001
	SHR+miR-193b-3p agomir+pLV-Arhgef9 versus SHR	0.0064
**Figure 9A**		
		P value
Relative protein expression of BAX	MiR-193b-3p antagomir versus antagomir NC	0.0018
	MiR-193b-3p antagomir+si-Arhgef9 versus antagomir NC	0.5814
Relative protein expression of BCL2	MiR-193b-3p antagomir versus antagomir NC	0.0035
	MiR-193b-3p antagomir+si-Arhgef9 versus antagomir NC	0.3426
Relative protein expression of Cleaved Caspase-3	MiR-193b-3p antagomir versus antagomir NC	0.001
	MiR-193b-3p antagomir+si-Arhgef9 versus antagomir NC	0.7248
**Figure 9B**		
		P value
Apoptosis cells	MiR-193b-3p antagomir versus antagomir NC	0.0054
	MiR-193b-3p antagomir+si-Arhgef9 versus antagomir NC	0.9909
**Figure 9C**		
		P value
Cell viability-24H	MiR-193b-3p antagomir versus antagomir NC	<0.0001
	MiR-193b-3p antagomir+si-Arhgef9 versus miR-193b-3p antagomir	0.0004
	MiR-193b-3p antagomir+si-Arhgef9 versus antagomir NC	0.217
Cell viability-48H	MiR-193b-3p antagomir versus antagomir NC	<0.0001

	MiR-193b-3p antagomir+si-Arhgef9 versus miR-193b-3p antagomir	<0.0001
	MiR-193b-3p antagomir+si-Arhgef9 versus antagomir NC	0.245
Cell viability-72H	MiR-193b-3p antagomir versus antagomir NC	<0.0001
	MiR-193b-3p antagomir+si-Arhgef9 versus miR-193b-3p antagomir	0.0019
	MiR-193b-3p antagomir+si-Arhgef9 versus antagomir NC	0.513
**Figure 10A**		
		P value
Mean arterial pressure (MAP)	SHR+miR-193b-3p agomir versus SHR	<0.0001
	SHR+miR-193b-3p agomir+pLV-Arhgef9 versus SHR+miR-193b-3p agomir	<0.0001
	SHR+miR-193b-3p agomir+pLV-Arhgef9 versus SHR	0.3221
Heart rate (HR)	SHR+miR-193b-3p agomir versus SHR	0.004
	SHR+miR-193b-3p agomir+pLV-Arhgef9 versus SHR+miR-193b-3p agomir	0.0056
	SHR+miR-193b-3p agomir+pLV-Arhgef9 versus SHR	0.9797
**Figure 10B**		
		P value
Renal sympathetic nerve activity (RSNA)	SHR+miR-193b-3p agomir versus SHR	<0.0001
	SHR+miR-193b-3p agomir+pLV-Arhgef9 versus SHR+miR-193b-3p agomir	<0.0001
	SHR+miR-193b-3p agomir+pLV-Arhgef9 versus SHR	0.271
**Figure 10C**		
		P value
Plasma NE	SHR+miR-193b-3p agomir versus SHR	<0.0001
	SHR+miR-193b-3p agomir+pLV-Arhgef9 versus SHR+miR-193b-3p agomir	<0.0001
	SHR+miR-193b-3p agomir+pLV-Arhgef9 versus SHR	0.9296
**Figure 10D**		
		P value
c-Fos positive TH+ neurons	SHR+miR-193b-3p agomir versus SHR	<0.0001
	SHR+miR-193b-3p agomir+pLV-Arhgef9 versus SHR+miR-193b-3p agomir	<0.0001
	SHR+miR-193b-3p agomir+pLV-Arhgef9 versus SHR	0.8312
**Figure S5**		
		P value
Relative expression of miR-193b-3p	RVLM versus Heart	0.0041
Relative expression of miR-346	RVLM versus Heart	0.003
Relative expression of miR-322-3p	RVLM versus Heart	0.0317
**Figure S7A**		
		P value
Relative expression of miR-193b-3p	SHR+miR-193b-3p agomir versus SHR	<0.0001
Relative expression of miR-346	SHR+miR-346 agomir versus SHR	<0.0001
Relative expression of miR-322-3p	SHR+miR-322-3p agomir versus SHR	<0.0001
**Figure S7B**		
		P value
Relative expression of miR-193b-3p	WKY+miR-193-3p antagomir versus WKY	0.0005
Relative expression of miR-346	WKY+miR-346 antagomir versus WKY	<0.0001
Relative expression of miR-322-3p	WKY+miR-322-3p antagomir versus WKY	0.0002
**Figure S7C**		
		P value
Relative expression of miR-193b-3p	SD+miR-193-3p antagomir versus SD	<0.0001
Relative expression of miR-346	SD+miR-346 antagomir versus SD	<0.0001

Relative expression of miR-322-3p	SD+miR-322-3p antagomir versus SD	<0.0001
**Figure S7E**		
		P value
Relative mRNA expression of Arhgef9	SHR versus SHR+miR-193b-3p agomir	<0.0001
	SHR+miR-193b-3p agomir+pLV-Arhgef9 versus SHR+miR-193b-3p agomir	<0.0001
**Figure S7F**		
		P value
Relative mRNA expression of Arhgef9	Antagomir NC versus miR-193b-3p antagomir	<0.0001
	MiR-193b-3p antagomir+pLV-Arhgef9-shRNA versus miR-193b-3p antagomir	<0.0001
**Figure S8**		
		P value
Mean arterial pressure (MAP)	SHR+miR-322-3p agomir versus SHR	<0.0001
Heart rate (HR)	SHR+miR-322-3p agomir versus SHR	<0.0001
**Figure S9**		
		P value
Mean arterial pressure (MAP)	WKY+miR-322-3p antagomir versus WKY	0.903
Heart rate (HR)	WKY+miR-322-3p antagomir versus WKY	0.7901
**Figure S10**		
		P value
Relative expression of miR-193b-3p	SD versus SHR	<0.0001
	WKY versus SHR	0.0001
Relative expression of miR-346	SD versus SHR	<0.0001
	WKY versus SHR	<0.0001
Relative expression of miR-322-3p	SD versus SHR	<0.0001
	WKY versus SHR	0.0006
**Figure S11A**		
		P value
Relative expression of miR-193b-3p	SD versus SHR	0.7706
	WKY versus SHR	0.6396
Relative expression of miR-346	SD versus SHR	0.2712
	WKY versus SHR	0.5295
Relative expression of miR-322-3p	SD versus SHR	0.2003
	WKY versus SHR	0.4363
**Figure S11B**		
		P value
Relative expression of miR-193b-3p	SD versus SHR	0.9045
	WKY versus SHR	0.5674
Relative expression of miR-346	SD versus SHR	0.556
	WKY versus SHR	0.2909
Relative expression of miR-322-3p	SD versus SHR	0.385
	WKY versus SHR	0.3808
**Figure S11C**		
		P value
Relative expression of miR-193b-3p	SD versus SHR	0.9113
	WKY versus SHR	0.7017
Relative expression of miR-346	SD versus SHR	0.9999
	WKY versus SHR	0.9865
Relative expression of miR-322-3p	SD versus SHR	0.9999
	WKY versus SHR	0.925
**Figure S12**		
		P value
Mean arterial pressure (MAP)	SD+miR-322-3p antagomir versus SD	0.6935

Heart rate (HR)	SD+miR-322-3p antagomir versus SD	0.9991

Table S4 Target genes of miR-193b-3p

miRNA ID	Gene ID	Gene Symbol	TargetScan_score	miranda_Energy
miR-193b-3p	ENSRNOG00000000033	Tmcc2	77	-12.74
miR-193b-3p	ENSRNOG00000000068	Ppp2r5a	89	-15.72
miR-193b-3p	ENSRNOG00000000121	Pigv	80	-19.68
miR-193b-3p	ENSRNOG00000000129	Phf24	83	-35.79
miR-193b-3p	ENSRNOG00000000151	Ldlrap1	69	-35.09
miR-193b-3p	ENSRNOG00000000246	Amz2	70	-17.09
miR-193b-3p	ENSRNOG00000000247	Mfsd11	64	-24
miR-193b-3p	ENSRNOG00000000465	Slc39a7	72	-21.87
miR-193b-3p	ENSRNOG00000000635	Arid5b	57	-18.23
miR-193b-3p	ENSRNOG00000000795	RT1-N3	63	-18.43
miR-193b-3p	ENSRNOG00000000904	Wdr95	74	-15.13
miR-193b-3p	ENSRNOG00000001050	Eif2ak1	78	-15.2
miR-193b-3p	ENSRNOG00000001086	Vps37b	76	-84.58
miR-193b-3p	ENSRNOG00000001134	Rfc5	51	-18.81
miR-193b-3p	ENSRNOG00000001169	Slc37a1	50	-23.24
miR-193b-3p	ENSRNOG00000001178	Sppl3	83	-25.62
miR-193b-3p	ENSRNOG00000001184	Pknox1	68	-18.75
miR-193b-3p	ENSRNOG00000001228	Pofut2	82	-26.83
miR-193b-3p	ENSRNOG00000001241	Iqce	56	-28
miR-193b-3p	ENSRNOG00000001273	Psmg3	60	-19.56
miR-193b-3p	ENSRNOG00000001288	Gpr146	74	-29.17
miR-193b-3p	ENSRNOG00000001377	Iqcd	96	-14.91
miR-193b-3p	ENSRNOG00000001397	Rbm19	54	-20.96
miR-193b-3p	ENSRNOG00000001451	Fkbp6	95	-22.34
miR-193b-3p	ENSRNOG00000001476	Cldn4	65	-18.35
miR-193b-3p	ENSRNOG00000001588	Hoxd13	99	-32.65
miR-193b-3p	ENSRNOG00000001680	Riox2	98	-12.65
miR-193b-3p	ENSRNOG00000045728	Crybg3	83	-17.44
miR-193b-3p	ENSRNOG00000001708	Dvl3	77	-22.24
miR-193b-3p	ENSRNOG00000001717	Opa1	68	-19.18
miR-193b-3p	ENSRNOG00000001793	Heg1	86	-25.81
miR-193b-3p	ENSRNOG00000001785	Etv5	50	-13.25
miR-193b-3p	ENSRNOG00000001811	Fgfr1op2	84	-19.34
miR-193b-3p	ENSRNOG00000001868	Crkl	64	-38.59
miR-193b-3p	ENSRNOG00000001877	Med15	97	-15.57
miR-193b-3p	ENSRNOG00000001989	Alcam	93	-19.36
miR-193b-3p	ENSRNOG00000002021	Son	78	-24.21
miR-193b-3p	ENSRNOG00000002069	Slc35a5	97	-16.36
miR-193b-3p	ENSRNOG00000002139	Cracd	93	-31.45
miR-193b-3p	ENSRNOG00000002185	Pgm2	77	-19.06
miR-193b-3p	ENSRNOG00000002227	Kit	98	-12.99
miR-193b-3p	ENSRNOG00000002244	Pdgfra	69	-18.75
miR-193b-3p	ENSRNOG00000002276	Phf6	89	-18.88
miR-193b-3p	ENSRNOG00000056248	Marf1	61	-13.5
miR-193b-3p	ENSRNOG00000002361	Prkg2	94	-13.11
miR-193b-3p	ENSRNOG00000002369	Rgs8	87	-24.59
miR-193b-3p	ENSRNOG00000002419	Plp1	97	-23.84
miR-193b-3p	ENSRNOG00000002407	Pdxdc1	84	-16.66
miR-193b-3p	ENSRNOG00000002418	Tgfb2	81	-15.21
miR-193b-3p	ENSRNOG00000025318	Scyl3	66	-22.08
miR-193b-3p	ENSRNOG00000002659	Ciita	64	-14.09
miR-193b-3p	ENSRNOG00000002695	Tfb2m	80	-27.92
miR-193b-3p	ENSRNOG00000002757	Tada2a	68	-16.06
miR-193b-3p	ENSRNOG00000002949	Pgs1	52	-12.87
miR-193b-3p	ENSRNOG00000002999	Timmdc1	75	-18.56
miR-193b-3p	ENSRNOG00000003105	Kif19	65	-15.95
miR-193b-3p	ENSRNOG00000003151	Bfar	63	-24.33
miR-193b-3p	ENSRNOG00000003147	Sqstm1	96	-15.01
miR-193b-3p	ENSRNOG00000003268	Maml1	93	-17.45

miR-193b-3p	ENSRNOG00000003183	Fmod	60	-60.01
miR-193b-3p	ENSRNOG00000003332	Nt5m	63	-16.46
miR-193b-3p	ENSRNOG00000003367	Tmem220	89	-21.64
miR-193b-3p	ENSRNOG00000003330	Acsf2	70	-15.06
miR-193b-3p	ENSRNOG00000003541	Pggt1b	86	-13.53
miR-193b-3p	ENSRNOG00000003544	Tbx4	93	-15.6
miR-193b-3p	ENSRNOG00000003543	Gpr143	96	-21.95
miR-193b-3p	ENSRNOG00000003603	Arhgap44	66	-15.02
miR-193b-3p	ENSRNOG00000042691	Armc7	86	-22.21
miR-193b-3p	ENSRNOG00000003545	Uchl5	94	-17.57
miR-193b-3p	ENSRNOG00000003600	Pnpt1	71	-12.22
miR-193b-3p	ENSRNOG00000003803	Zdhhc17	60	-27.9
miR-193b-3p	ENSRNOG00000003947	Ntn1	77	-38.43
miR-193b-3p	ENSRNOG00000003955	Spata7	99	-15.71
miR-193b-3p	ENSRNOG00000003821	Rptor	85	-12.51
miR-193b-3p	ENSRNOG00000003948	Llgl1	90	-21.21
miR-193b-3p	ENSRNOG00000004112	RGD1308775	60	-17.7
miR-193b-3p	ENSRNOG00000004195	Dtl	70	-22.98
miR-193b-3p	ENSRNOG00000003972	Tshr	70	-13.88
miR-193b-3p	ENSRNOG00000004351	Slc25a29	81	-12.73
miR-193b-3p	ENSRNOG00000004077	Kcnc2	79	-16.95
miR-193b-3p	ENSRNOG00000004400	Avpr1a	64	-32.7
miR-193b-3p	ENSRNOG00000004300	Gtf2a1	54	-18.51
miR-193b-3p	ENSRNOG00000004367	Elk3	85	-23.18
miR-193b-3p	ENSRNOG00000004310	Caskin2	52	-24.72
miR-193b-3p	ENSRNOG00000004534	Spdya	50	-40.1
miR-193b-3p	ENSRNOG00000004641	Sstr4	94	-16.84
miR-193b-3p	ENSRNOG00000004699	Fibin	81	-20.13
miR-193b-3p	ENSRNOG00000004382	Arel1	94	-13.92
miR-193b-3p	ENSRNOG00000004826	Sos2	95	-14.95
miR-193b-3p	ENSRNOG00000004874	Flrt3	80	-16.15
miR-193b-3p	ENSRNOG00000004783	Fam171b	90	-15.7
miR-193b-3p	ENSRNOG00000004786	Cyp4f1	73	-24.52
miR-193b-3p	ENSRNOG00000038921	Tex35	85	-22.85
miR-193b-3p	ENSRNOG00000005132	Slc52a3	70	-15.09
miR-193b-3p	ENSRNOG00000005083	Rtfdc1	74	-14.95
miR-193b-3p	ENSRNOG00000004846	Atp6v1c1	76	-22.46
miR-193b-3p	ENSRNOG00000005154	Alkal2	57	-13.9
miR-193b-3p	ENSRNOG00000005332	Csdc2	64	-77.08
miR-193b-3p	ENSRNOG00000005660	Fam110c	98	-15.41
miR-193b-3p	ENSRNOG00000068642	Slc6a20	99	-12.54
miR-193b-3p	ENSRNOG00000005551	Derl1	67	-46.09
miR-193b-3p	ENSRNOG00000004973	Ppp2r5c	90	-15.29
miR-193b-3p	ENSRNOG00000005719	Emc4	57	-21.66
miR-193b-3p	ENSRNOG00000005589	Dhrs7	78	-12.95
miR-193b-3p	ENSRNOG00000005667	Astn1	57	-36.48
miR-193b-3p	ENSRNOG00000005707	Bfsp1	93	-21.39
miR-193b-3p	ENSRNOG00000005933	Yap1	67	-20.75
miR-193b-3p	ENSRNOG00000005342	Rassf5	67	-12.54
miR-193b-3p	ENSRNOG00000005602	Mthfd1	80	-19.01
miR-193b-3p	ENSRNOG00000006412	Zhx1	85	-22.89
miR-193b-3p	ENSRNOG00000005733	Cpsf2	88	-17.78
miR-193b-3p	ENSRNOG00000006617	Cntnap2	73	-13.99
miR-193b-3p	ENSRNOG00000006636	Otud6b	99	-12.22
miR-193b-3p	ENSRNOG00000006551	Dcaf15	54	-15.83
miR-193b-3p	ENSRNOG00000006736	Ccr3	79	-25.17
miR-193b-3p	ENSRNOG00000006802	Lrrn1	79	-14.07
miR-193b-3p	ENSRNOG00000006778	Mmp19	98	-14.26
miR-193b-3p	ENSRNOG00000006846	En2	86	-18.76
miR-193b-3p	ENSRNOG00000006727	Dtd2	91	-13.97
miR-193b-3p	ENSRNOG00000006901	Ndufa7	50	-26.15

miR-193b-3p	ENSRNOG00000006628	Dusp16	92	-12.98
miR-193b-3p	ENSRNOG00000006990	Grb7	90	-19.06
miR-193b-3p	ENSRNOG00000007131	Paxip1	93	-21.78
miR-193b-3p	ENSRNOG00000007202	Sema3d	88	-16.97
miR-193b-3p	ENSRNOG00000006940	Ncf4	65	-17.58
miR-193b-3p	ENSRNOG00000007300	C1qtnf6	83	-13.14
miR-193b-3p	ENSRNOG00000006763	Rbm18	91	-13.6
miR-193b-3p	ENSRNOG00000006919	Clvs1	66	-13.99
miR-193b-3p	ENSRNOG00000007578	Zfp830	87	-21.52
miR-193b-3p	ENSRNOG00000007412	Dok1	96	-22.4
miR-193b-3p	ENSRNOG00000007604	Igsf8	78	-15.33
miR-193b-3p	ENSRNOG00000007454	Aloxe3	96	-17.53
miR-193b-3p	ENSRNOG00000007478	Cry2	73	-33.35
miR-193b-3p	ENSRNOG00000042467	Ttc4	54	-15.06
miR-193b-3p	ENSRNOG00000007821	Dyrk2	85	-15.29
miR-193b-3p	ENSRNOG00000007678	Zfp263	79	-23.18
miR-193b-3p	ENSRNOG00000007564	Evc	88	-19.05
miR-193b-3p	ENSRNOG00000008026	C2cd4c	66	-20.24
miR-193b-3p	ENSRNOG00000008027	Cavin4	52	-16.75
miR-193b-3p	ENSRNOG00000007687	Sema7a	52	-17.9
miR-193b-3p	ENSRNOG00000007764	Frmd4b	62	-13.76
miR-193b-3p	ENSRNOG00000007955	Timp4	95	-19.11
miR-193b-3p	ENSRNOG00000008169	Slc24a2	56	-14.15
miR-193b-3p	ENSRNOG00000007456	Calb1	92	-13.97
miR-193b-3p	ENSRNOG00000007519	Tmem43	87	-18.93
miR-193b-3p	ENSRNOG00000008049	Max	74	-22.38
miR-193b-3p	ENSRNOG00000008135	Pla2g4f	68	-23.12
miR-193b-3p	ENSRNOG00000008137	Cdk18	62	-14.87
miR-193b-3p	ENSRNOG00000037134	Shc4	63	-22.06
miR-193b-3p	ENSRNOG00000007970	Plxnc1	94	-16.36
miR-193b-3p	ENSRNOG00000008142	Brpf1	52	-37.12
miR-193b-3p	ENSRNOG00000007719	Ccnc	95	-16.87
miR-193b-3p	ENSRNOG00000008445	Dact1	93	-13.26
miR-193b-3p	ENSRNOG00000008696	Nopchap1	61	-15.8
miR-193b-3p	ENSRNOG00000008634	Cc2d1b	61	-19.76
miR-193b-3p	ENSRNOG00000009068	Phlda3	64	-16.28
miR-193b-3p	ENSRNOG00000008927	Hbp1	57	-13.53
miR-193b-3p	ENSRNOG00000008354	Slc9a8	70	-14.39
miR-193b-3p	ENSRNOG00000008557	Abcb8	52	-19.28
miR-193b-3p	ENSRNOG00000009149	Jam3	98	-17.3
miR-193b-3p	ENSRNOG00000008167	Abhd6	55	-12.39
miR-193b-3p	ENSRNOG00000009245	Exosc2	95	-18.07
miR-193b-3p	ENSRNOG00000009156	Tra2a	83	-38.2
miR-193b-3p	ENSRNOG00000009196	Rc3h2	53	-29.03
miR-193b-3p	ENSRNOG00000009094	Nudt4	50	-33.8
miR-193b-3p	ENSRNOG00000009102	Fermt2	53	-16.85
miR-193b-3p	ENSRNOG00000009152	Caprin1	87	-11.96
miR-193b-3p	ENSRNOG00000009110	Psen1	80	-13.11
miR-193b-3p	ENSRNOG00000009113	Marcksl1	92	-15.16
miR-193b-3p	ENSRNOG00000009589	Angptl7	92	-15.39
miR-193b-3p	ENSRNOG00000009888	Timm8b	91	-20.37
miR-193b-3p	ENSRNOG00000009910	Swap70	79	-14
miR-193b-3p	ENSRNOG00000009871	Piwil2	69	-34.36
miR-193b-3p	ENSRNOG00000010018	Clec4a3	50	-14.33
miR-193b-3p	ENSRNOG00000009934	Emc3	92	-13.71
miR-193b-3p	ENSRNOG00000010347	Styk1	80	-20.3
miR-193b-3p	ENSRNOG00000010435	Nupl2	74	-12.54
miR-193b-3p	ENSRNOG00000010461	Gpx8	95	-17.91
miR-193b-3p	ENSRNOG00000010438	Cpt1b	62	-19.65
miR-193b-3p	ENSRNOG00000010457	Vash1	64	-13.41
miR-193b-3p	ENSRNOG00000010600	Cysrt1	55	-45.95

miR-193b-3p	ENSRNOG00000010665	Ccr7	69	-42.79
miR-193b-3p	ENSRNOG00000010610	Hpgd	83	-25.95
miR-193b-3p	ENSRNOG00000010516	Plau	92	-14.11
miR-193b-3p	ENSRNOG00000010731	Gpm6a	69	-15.68
miR-193b-3p	ENSRNOG00000010794	Dennd3	96	-21.73
miR-193b-3p	ENSRNOG00000010712	Terf2ip	97	-16.13
miR-193b-3p	ENSRNOG00000010789	Dusp7	80	-65.66
miR-193b-3p	ENSRNOG00000010914	Pigh	75	-13.43
miR-193b-3p	ENSRNOG00000011120	Radx	88	-14.69
miR-193b-3p	ENSRNOG00000010906	Ccl5	71	-20.15
miR-193b-3p	ENSRNOG00000011316	Fam167a	56	-18.17
miR-193b-3p	ENSRNOG00000011424	Cldn23	98	-13.63
miR-193b-3p	ENSRNOG00000010702	Ube3c	71	-14.34
miR-193b-3p	ENSRNOG00000011401	Usp50	54	-17.08
miR-193b-3p	ENSRNOG00000010895	Tmem30a	71	-14.63
miR-193b-3p	ENSRNOG00000011491	Dnajc13	95	-14.14
miR-193b-3p	ENSRNOG00000011451	Lrp3	58	-40.72
miR-193b-3p	ENSRNOG00000011216	Tbl1xr1	80	-12.61
miR-193b-3p	ENSRNOG00000011467	Uck1	75	-20.97
miR-193b-3p	ENSRNOG00000011752	Sh3d19	69	-16.92
miR-193b-3p	ENSRNOG00000011918	Vsx2	62	-14.43
miR-193b-3p	ENSRNOG00000011821	S100a4	74	-20.92
miR-193b-3p	ENSRNOG00000022402	Luzp1	82	-39.32
miR-193b-3p	ENSRNOG00000011937	Sgtb	83	-14.18
miR-193b-3p	ENSRNOG00000010752	Tmem41b	83	-12.61
miR-193b-3p	ENSRNOG00000012183	Glrx	68	-14.24
miR-193b-3p	ENSRNOG00000012164	Plppr2	68	-19.35
miR-193b-3p	ENSRNOG00000012258	Rras2	95	-14.79
miR-193b-3p	ENSRNOG00000012811	Spint1	68	-17.24
miR-193b-3p	ENSRNOG00000012803	Wdr61	58	-18.92
miR-193b-3p	ENSRNOG00000012347	Gata2	54	-17.22
miR-193b-3p	ENSRNOG00000012866	Nek8	78	-20.27
miR-193b-3p	ENSRNOG00000012862	Spsb4	77	-15.64
miR-193b-3p	ENSRNOG00000012840	Sparc	68	-27.64
miR-193b-3p	ENSRNOG00000013171	Grm2	97	-20.18
miR-193b-3p	ENSRNOG00000013017	Arnt2	56	-20.96
miR-193b-3p	ENSRNOG00000013160	Sash1	96	-15.81
miR-193b-3p	ENSRNOG00000013263	Odad3	85	-19.38
miR-193b-3p	ENSRNOG00000013282	Mctp1	76	-18.87
miR-193b-3p	ENSRNOG00000013491	Setx	67	-16.3
miR-193b-3p	ENSRNOG00000013300	Atp5if1	94	-14.95
miR-193b-3p	ENSRNOG00000013526	Rassf4	97	-13.91
miR-193b-3p	ENSRNOG00000013514	Maf1	94	-16.78
miR-193b-3p	ENSRNOG00000013647	Polm	60	-15.71
miR-193b-3p	ENSRNOG00000013751	Plpbp	93	-11.93
miR-193b-3p	ENSRNOG00000049489	Rad54l2	77	-18.05
miR-193b-3p	ENSRNOG00000013253	Armc1	92	-16.01
miR-193b-3p	ENSRNOG00000013902	P2ry12	98	-18.65
miR-193b-3p	ENSRNOG00000031612	Gls2	80	-18.11
miR-193b-3p	ENSRNOG00000013232	Nr6a1	81	-19.67
miR-193b-3p	ENSRNOG00000023851	Igsf3	67	-40.49
miR-193b-3p	ENSRNOG00000014083	Iqsec3	91	-62.18
miR-193b-3p	ENSRNOG00000013763	Erlin2	71	-18.96
miR-193b-3p	ENSRNOG00000014000	Kif2a	62	-20.82
miR-193b-3p	ENSRNOG00000014248	Erbb4	98	-12.51
miR-193b-3p	ENSRNOG00000014454	Ap1m1	55	-17.94
miR-193b-3p	ENSRNOG00000014524	S1pr3	69	-19.13
miR-193b-3p	ENSRNOG00000014440	Josd1	89	-13.1
miR-193b-3p	ENSRNOG00000014311	Slc7a8	66	-16.14
miR-193b-3p	ENSRNOG00000014838	Glipr2	79	-15.49
miR-193b-3p	ENSRNOG00000014828	Avpi1	64	-15.55

miR-193b-3p	ENSRNOG00000014874	Zfyve28	73	-23.73
miR-193b-3p	ENSRNOG00000015077	Acsf3	69	-20.79
miR-193b-3p	ENSRNOG00000015143	Siah1	77	-11.96
miR-193b-3p	ENSRNOG00000014917	Nck1	64	-23.02
miR-193b-3p	ENSRNOG00000015732	Bcl2l2	50	-60.8
miR-193b-3p	ENSRNOG00000015071	Zim1	75	-31.54
miR-193b-3p	ENSRNOG00000015158	Pikfyve	93	-13.41
miR-193b-3p	ENSRNOG00000015133	Kmt2a	78	-16.03
miR-193b-3p	ENSRNOG00000015288	Dip2c	71	-17.95
miR-193b-3p	ENSRNOG00000014776	Adcy7	82	-17.02
miR-193b-3p	ENSRNOG00000015614	Ppp1r16b	97	-31.32
miR-193b-3p	ENSRNOG00000015727	Loxl4	58	-22.57
miR-193b-3p	ENSRNOG00000015567	Slc9a2	61	-41.33
miR-193b-3p	ENSRNOG00000015285	Lrp4	93	-17.84
miR-193b-3p	ENSRNOG00000015913	Tspan5	90	-18.59
miR-193b-3p	ENSRNOG00000015970	Tbc1d13	64	-17.17
miR-193b-3p	ENSRNOG00000015928	Dhx35	73	-16.56
miR-193b-3p	ENSRNOG00000015633	Cul3	95	-19.36
miR-193b-3p	ENSRNOG00000015686	Cert1	90	-23.2
miR-193b-3p	ENSRNOG00000016055	Fkrp	97	-16.67
miR-193b-3p	ENSRNOG00000016252	Mblac2	88	-13.88
miR-193b-3p	ENSRNOG00000016212	Ankfy1	81	-43.68
miR-193b-3p	ENSRNOG00000016010	Mul1	69	-15.92
miR-193b-3p	ENSRNOG00000016244	Mical2	91	-13.41
miR-193b-3p	ENSRNOG00000016279	Zfp865	83	-22.71
miR-193b-3p	ENSRNOG00000016312	Catsper4	56	-12.54
miR-193b-3p	ENSRNOG00000016468	Tpmt	87	-12.51
miR-193b-3p	ENSRNOG00000016303	Zfp236	86	-12.36
miR-193b-3p	ENSRNOG00000016490	Nox3	70	-17.92
miR-193b-3p	ENSRNOG00000016688	Ston1	72	-19.63
miR-193b-3p	ENSRNOG00000016595	Hhex	56	-15.6
miR-193b-3p	ENSRNOG00000016654	Galr1	95	-14.62
miR-193b-3p	ENSRNOG00000016427	Letm1	72	-22.77
miR-193b-3p	ENSRNOG00000016810	Stmn1	92	-17.91
miR-193b-3p	ENSRNOG00000016815	Tmem135	73	-20.68
miR-193b-3p	ENSRNOG00000016863	Pnma8b	71	-15.96
miR-193b-3p	ENSRNOG00000016875	Cbx7	81	-41.27
miR-193b-3p	ENSRNOG00000022054	Paqr7	79	-16.94
miR-193b-3p	ENSRNOG00000016838	Pla2g5	52	-12.54
miR-193b-3p	ENSRNOG00000017017	Zbtb43	99	-36.59
miR-193b-3p	ENSRNOG00000016791	Chka	55	-22.6
miR-193b-3p	ENSRNOG00000016913	Stk36	85	-15.71
miR-193b-3p	ENSRNOG00000016932	Simc1	94	-24.26
miR-193b-3p	ENSRNOG00000017188	Cyp27a1	79	-13.26
miR-193b-3p	ENSRNOG00000017318	Zfp322a	86	-12.78
miR-193b-3p	ENSRNOG00000017208	Cspg4	52	-25.81
miR-193b-3p	ENSRNOG00000017311	Me3	92	-14.41
miR-193b-3p	ENSRNOG00000017206	Igfbp5	80	-15.46
miR-193b-3p	ENSRNOG00000017354	Zyx	51	-16.04
miR-193b-3p	ENSRNOG00000017514	Btn1a1	59	-21.36
miR-193b-3p	ENSRNOG00000017680	Dennd2d	84	-15.68
miR-193b-3p	ENSRNOG00000017684	Fbxl22	73	-18.61
miR-193b-3p	ENSRNOG00000017836	Rrp36	71	-21.58
miR-193b-3p	ENSRNOG00000017693	Slc2a5	76	-26.7
miR-193b-3p	ENSRNOG00000017930	Lpcat1	58	-14.08
miR-193b-3p	ENSRNOG00000017775	Slc5a1	88	-21.28
miR-193b-3p	ENSRNOG00000017981	Mcm10	76	-19.53
miR-193b-3p	ENSRNOG00000017773	Crispld1	89	-20.29
miR-193b-3p	ENSRNOG00000026277	Zc3h6	85	-15.85
miR-193b-3p	ENSRNOG00000018214	Bok	52	-16.07
miR-193b-3p	ENSRNOG00000018385	Chrm1	78	-22.08

miR-193b-3p	ENSRNOG00000018330	Fam83f	77	-38.62
miR-193b-3p	ENSRNOG00000018411	Pold3	62	-16.82
miR-193b-3p	ENSRNOG00000018457	Ptpa	91	-13.55
miR-193b-3p	ENSRNOG00000018665	Bud13	93	-16.94
miR-193b-3p	ENSRNOG00000049179	Pidd1	78	-17.62
miR-193b-3p	ENSRNOG00000018906	Ghdc	94	-17.92
miR-193b-3p	ENSRNOG00000018847	Stx5	77	-30.24
miR-193b-3p	ENSRNOG00000018853	Slc28a3	70	-20.16
miR-193b-3p	ENSRNOG00000018931	Dis3l2	60	-16.89
miR-193b-3p	ENSRNOG00000018898	Mpi	75	-21.12
miR-193b-3p	ENSRNOG00000067387	Sbk1	87	-52.32
miR-193b-3p	ENSRNOG00000019132	Fv1	78	-50.17
miR-193b-3p	ENSRNOG00000019169	RGD1562218	59	-22.97
miR-193b-3p	ENSRNOG00000029336	Zfp180	87	-13.17
miR-193b-3p	ENSRNOG00000019174	Chtf18	85	-16.14
miR-193b-3p	ENSRNOG00000019349	Sf3a2	81	-13.01
miR-193b-3p	ENSRNOG00000019296	Gnat2	79	-15.65
miR-193b-3p	ENSRNOG00000019316	Sh3bp4	50	-17.84
miR-193b-3p	ENSRNOG00000019464	Ttc9c	65	-13.08
miR-193b-3p	ENSRNOG00000019559	Eaf1	98	-13.8
miR-193b-3p	ENSRNOG00000019570	Gng3	86	-45.09
miR-193b-3p	ENSRNOG00000019660	Spib	67	-19.52
miR-193b-3p	ENSRNOG00000019763	Mlph	57	-19.93
miR-193b-3p	ENSRNOG00000019883	Pak4	93	-14.46
miR-193b-3p	ENSRNOG00000019971	Kxd1	60	-15.33
miR-193b-3p	ENSRNOG00000019930	Rhot2	58	-21.9
miR-193b-3p	ENSRNOG00000019943	Slc7a6	85	-14.01
miR-193b-3p	ENSRNOG00000020110	B4gat1	81	-55.25
miR-193b-3p	ENSRNOG00000027924	Fam83g	79	-13.84
miR-193b-3p	ENSRNOG00000020254	Per2	63	-14.77
miR-193b-3p	ENSRNOG00000020361	Ip6k2	95	-22.37
miR-193b-3p	ENSRNOG00000020434	Ascl2	78	-15.87
miR-193b-3p	ENSRNOG00000020468	Stard4	52	-12.54
miR-193b-3p	ENSRNOG00000020441	Vps25	51	-19.72
miR-193b-3p	ENSRNOG00000020692	Tmem216	57	-15.83
miR-193b-3p	ENSRNOG00000020737	Cdc25a	77	-13.1
miR-193b-3p	ENSRNOG00000020797	She	83	-27.78
miR-193b-3p	ENSRNOG00000020875	Celf3	54	-20.38
miR-193b-3p	ENSRNOG00000020879	Nags	67	-18.25
miR-193b-3p	ENSRNOG00000020904	Cdc42ep2	63	-42.11
miR-193b-3p	ENSRNOG00000020893	Snx27	86	-16.7
miR-193b-3p	ENSRNOG00000020947	Egln2	93	-15.62
miR-193b-3p	ENSRNOG00000024363	Sertad1	74	-12.77
miR-193b-3p	ENSRNOG00000021032	Sphk2	72	-13.41
miR-193b-3p	ENSRNOG00000021039	Fam83e	81	-18.73
miR-193b-3p	ENSRNOG00000021054	Men1	58	-28.95
miR-193b-3p	ENSRNOG00000021218	Pias3	98	-12.51
miR-193b-3p	ENSRNOG00000030724	Adamdec1	76	-14.76
miR-193b-3p	ENSRNOG00000028074	Lcn10	82	-40.33
miR-193b-3p	ENSRNOG00000024641	Nyap1	63	-12.54
miR-193b-3p	ENSRNOG00000003781	Atp10b	75	-25.7
miR-193b-3p	ENSRNOG00000024632	Atf6	61	-21.7
miR-193b-3p	ENSRNOG00000023457	Stpg2	79	-17.82
miR-193b-3p	ENSRNOG00000026408	Rnf169	86	-40.17
miR-193b-3p	ENSRNOG00000025117	Noxo1	52	-25.25
miR-193b-3p	ENSRNOG00000021828	Coro1b	61	-17.78
miR-193b-3p	ENSRNOG00000027839	Ptk2b	51	-19.18
miR-193b-3p	ENSRNOG00000021966	Il17rd	63	-27.12
miR-193b-3p	ENSRNOG00000026248	Nmt2	88	-13.99
miR-193b-3p	ENSRNOG00000021475	Ldah	60	-17.34
miR-193b-3p	ENSRNOG00000028523	Tctn1	82	-24.35

miR-193b-3p	ENSRNOG00000037562	Zbtb42	68	-12.92
miR-193b-3p	ENSRNOG00000024025	Edc4	82	-22.53
miR-193b-3p	ENSRNOG00000005933	Yap1	75	-20.75
miR-193b-3p	ENSRNOG00000027617	Ttc26	72	-15.8
miR-193b-3p	ENSRNOG00000037798	Erich4	90	-18.23
miR-193b-3p	ENSRNOG00000028344	Mmp11	90	-24.35
miR-193b-3p	ENSRNOG00000026085	Rabl3	94	-14.34
miR-193b-3p	ENSRNOG00000028292	Gart	68	-20.82
miR-193b-3p	ENSRNOG00000010626	Sphk1	60	-21.77
miR-193b-3p	ENSRNOG00000021731	N4bp3	66	-22.31
miR-193b-3p	ENSRNOG00000019052	Ankzf1	93	-21.35
miR-193b-3p	ENSRNOG00000024040	Mb21d2	88	-18.8
miR-193b-3p	ENSRNOG00000023508	Mfsd2b	95	-11.93
miR-193b-3p	ENSRNOG00000009481	Ddhd1	56	-16.33
miR-193b-3p	ENSRNOG00000001469	Eln	67	-22.42
miR-193b-3p	ENSRNOG00000023760	Plekhm3	84	-49.82
miR-193b-3p	ENSRNOG00000018885	Zfp60	97	-18.64
miR-193b-3p	ENSRNOG00000015554	Ankdd1a	52	-42.59
miR-193b-3p	ENSRNOG00000027368	Ubald1	79	-18.76
miR-193b-3p	ENSRNOG00000028329	Zfp438	67	-19.46
miR-193b-3p	ENSRNOG00000024796	Lrrc47	97	-14.99
miR-193b-3p	ENSRNOG00000024454	Ccdc149	55	-12.91
miR-193b-3p	ENSRNOG00000021856	Lat2	80	-31.48
miR-193b-3p	ENSRNOG00000001613	Zbtb11	89	-15.52
miR-193b-3p	ENSRNOG00000025444	Lipo1	85	-12.16
miR-193b-3p	ENSRNOG00000026316	Wdr43	93	-21.04
miR-193b-3p	ENSRNOG00000023238	Dgkd	61	-17.21
miR-193b-3p	ENSRNOG00000026742	Ypel5	64	-27.06
miR-193b-3p	ENSRNOG00000025051	Tal1	94	-18.78
miR-193b-3p	ENSRNOG00000025951	Taar2	89	-23.66
miR-193b-3p	ENSRNOG00000023352	Fam78a	62	-24.85
miR-193b-3p	ENSRNOG00000008195	Ywhaz	66	-20.62
miR-193b-3p	ENSRNOG00000025806	Prr3	64	-20.66
miR-193b-3p	ENSRNOG00000023643	Mmp17	94	-22.5
miR-193b-3p	ENSRNOG00000009148	Cyb5rl	56	-17.4
miR-193b-3p	ENSRNOG00000025670	Shisa3	75	-20
miR-193b-3p	ENSRNOG00000021886	B4galt7	52	-29.12
miR-193b-3p	ENSRNOG00000026212	Micall1	79	-15.88
miR-193b-3p	ENSRNOG00000024918	Fam229b	96	-21.98
miR-193b-3p	ENSRNOG00000002667	Lamc2	97	-14.29
miR-193b-3p	ENSRNOG00000026770	Tspy26	74	-14.54
miR-193b-3p	ENSRNOG00000024194	Rsf1	69	-18.23
miR-193b-3p	ENSRNOG00000006844	Dnajc14	95	-15.43
miR-193b-3p	ENSRNOG00000021615	Mettl26	85	-15.86
miR-193b-3p	ENSRNOG00000023322	Ostc	79	-19.4
miR-193b-3p	ENSRNOG00000026036	Pdyn	68	-18.81
miR-193b-3p	ENSRNOG00000010748	Mtus1	69	-26.53
miR-193b-3p	ENSRNOG00000026053	Grem1	88	-21.3
miR-193b-3p	ENSRNOG00000016778	Cops7a	94	-24.26
miR-193b-3p	ENSRNOG00000022698	Vsig10	67	-13.41
miR-193b-3p	ENSRNOG00000064601	Mlec	81	-20.95
miR-193b-3p	ENSRNOG00000022593	Pdpr	56	-26.76
miR-193b-3p	ENSRNOG00000000567	Unc5b	99	-22.13
miR-193b-3p	ENSRNOG00000020295	Plekhn1	61	-12.55
miR-193b-3p	ENSRNOG00000028335	Fat4	98	-20.48
miR-193b-3p	ENSRNOG00000028456	Ush1g	68	-13.99
miR-193b-3p	ENSRNOG00000023467	Fam168b	57	-16.98
miR-193b-3p	ENSRNOG00000023908	Egfem1	93	-14.67
miR-193b-3p	ENSRNOG00000024264	Amz1	72	-13.94
miR-193b-3p	ENSRNOG00000069712	RGD1564827	88	-16.21
miR-193b-3p	ENSRNOG00000028708	Ntsr1	70	-22.27

miR-193b-3p	ENSRNOG00000029543	Cish	68	-15.41
miR-193b-3p	ENSRNOG00000019976	Spata24	92	-33.9
miR-193b-3p	ENSRNOG00000010389	Ndrg2	83	-25.67
miR-193b-3p	ENSRNOG00000032394	Tymp	72	-46.12
miR-193b-3p	ENSRNOG00000014903	Zfyve27	78	-18.39
miR-193b-3p	ENSRNOG00000023781	Plec	90	-14.84
miR-193b-3p	ENSRNOG00000015380	Jup	64	-21.31
miR-193b-3p	ENSRNOG00000034107	1110038F14Rik	75	-19.61
miR-193b-3p	ENSRNOG00000000985	Cpsf4	76	-16.78
miR-193b-3p	ENSRNOG00000019319	Fchsd2	84	-21.8
miR-193b-3p	ENSRNOG00000031232	Nrp2	76	-15.44
miR-193b-3p	ENSRNOG00000009653	Numb	80	-14.24
miR-193b-3p	ENSRNOG00000031675	Panx3	80	-17.98
miR-193b-3p	ENSRNOG00000037845	Cysltr1	91	-13.99
miR-193b-3p	ENSRNOG00000034078	Mxi1	66	-22.13
miR-193b-3p	ENSRNOG00000042833	Prp2l1	87	-22.87
miR-193b-3p	ENSRNOG00000021758	Tnpo3	89	-16.78
miR-193b-3p	ENSRNOG00000003554	Piga	96	-24.01
miR-193b-3p	ENSRNOG00000010944	Hyou1	52	-22.28
miR-193b-3p	ENSRNOG00000032819	Vom1r62	83	-19.06
miR-193b-3p	ENSRNOG00000031367	Hprt1	99	-17.24
miR-193b-3p	ENSRNOG00000033361	Slc39a5	93	-52.4
miR-193b-3p	ENSRNOG00000065730	Ctsm	85	-16.21
miR-193b-3p	ENSRNOG00000065347	Adgrl2	84	-12
miR-193b-3p	ENSRNOG00000010174	Enpp4	66	-21.68
miR-193b-3p	ENSRNOG00000019861	Tollip	66	-19.49
miR-193b-3p	ENSRNOG00000001645	Filip1l	70	-16.95
miR-193b-3p	ENSRNOG00000009932	Mctp2	93	-12.88
miR-193b-3p	ENSRNOG00000014471	B3gnt6	95	-26.16
miR-193b-3p	ENSRNOG00000020710	Raver1	53	-13.87
miR-193b-3p	ENSRNOG00000028837	AC128207.1	83	-18.55
miR-193b-3p	ENSRNOG00000031443	Havcr2	74	-14.35
miR-193b-3p	ENSRNOG00000031824	Slc44a2	62	-35.69
miR-193b-3p	ENSRNOG00000024849	Tor1aip2	54	-39.8
miR-193b-3p	ENSRNOG00000010213	Fgd5	68	-15.85
miR-193b-3p	ENSRNOG00000015019	Spg21	82	-13.98
miR-193b-3p	ENSRNOG00000021660	Nprl2	91	-17.18
miR-193b-3p	ENSRNOG00000020360	Clcc1	90	-17.4
miR-193b-3p	ENSRNOG00000024688	Erfe	70	-30.18
miR-193b-3p	ENSRNOG00000001774	Lrch3	91	-14.08
miR-193b-3p	ENSRNOG00000016566	Arhgap39	52	-14.74
miR-193b-3p	ENSRNOG00000001094	Zfp316	52	-17.81
miR-193b-3p	ENSRNOG00000004320	Tgm4	82	-12.82
miR-193b-3p	ENSRNOG00000014152	Kcnip3	65	-17.84
miR-193b-3p	ENSRNOG00000007400	Srebf2	91	-19.39
miR-193b-3p	ENSRNOG00000020862	Ptpn23	91	-17.83
miR-193b-3p	ENSRNOG00000021176	Mtmr11	94	-21.42
miR-193b-3p	ENSRNOG00000020297	Gon4l	89	-12.97
miR-193b-3p	ENSRNOG00000010835	Dmbx1	75	-22.3
miR-193b-3p	ENSRNOG00000008193	Cr1l	85	-15.81
miR-193b-3p	ENSRNOG00000016182	Tgfa	96	-12.65
miR-193b-3p	ENSRNOG00000023972	Col4a2	69	-18.36
miR-193b-3p	ENSRNOG00000037835	Catip	95	-27.01
miR-193b-3p	ENSRNOG00000030719	Csmd1	91	-40.65
miR-193b-3p	ENSRNOG00000000638	Zfp365	58	-25.72
miR-193b-3p	ENSRNOG00000001709	Ap2m1	88	-12.67
miR-193b-3p	ENSRNOG00000005234	Gxylt1	80	-53.13
miR-193b-3p	ENSRNOG00000017146	Nfatc1	58	-46.9
miR-193b-3p	ENSRNOG00000015888	Larp4b	60	-13.93
miR-193b-3p	ENSRNOG00000038505	Gmnc	94	-13.41
miR-193b-3p	ENSRNOG00000007419	Pank3	85	-82.84

miR-193b-3p	ENSRNOG00000010332	Nipsnap3b	85	-18.36
miR-193b-3p	ENSRNOG00000002473	Odr4	69	-15.59
miR-193b-3p	ENSRNOG00000002473	Odr4	94	-15.59
miR-193b-3p	ENSRNOG00000022312	Lrsam1	59	-33.74
miR-193b-3p	ENSRNOG00000019688	Diaph1	87	-19.22
miR-193b-3p	ENSRNOG00000001253	Eif3b	51	-23.64
miR-193b-3p	ENSRNOG00000004449	E2f6	97	-16.71
miR-193b-3p	ENSRNOG00000011956	Aktip	60	-18.45
miR-193b-3p	ENSRNOG00000039740	Cenpk	98	-18.99
miR-193b-3p	ENSRNOG00000016250	Ammecr1l	82	-34.21
miR-193b-3p	ENSRNOG00000018876	Entr1	66	-26.51
miR-193b-3p	ENSRNOG00000016528	Ppp1r21	82	-15.56
miR-193b-3p	ENSRNOG00000011871	Kat2b	98	-13.27
miR-193b-3p	ENSRNOG00000042245	Dcaf7	98	-27.8
miR-193b-3p	ENSRNOG00000007733	Arhgef9	89	-16.36
miR-193b-3p	ENSRNOG00000042449	Cdc42se2	70	-13.21
miR-193b-3p	ENSRNOG00000018755	Acss2	59	-16.39
miR-193b-3p	ENSRNOG00000028062	Arpc5	82	-14.03
miR-193b-3p	ENSRNOG00000024954	Mgat5b	78	-18.88
miR-193b-3p	ENSRNOG00000004402	Lpgat1	66	-18.2
miR-193b-3p	ENSRNOG00000042950	Slc5a9	63	-19.73
miR-193b-3p	ENSRNOG00000004060	Calm1	72	-61.24
miR-193b-3p	ENSRNOG00000013934	Dennd2b	73	-24.36
miR-193b-3p	ENSRNOG00000007590	Eya1	87	-16.27
miR-193b-3p	ENSRNOG00000019964	Taok2	55	-22.82
miR-193b-3p	ENSRNOG00000018988	Ing5	84	-37.94
miR-193b-3p	ENSRNOG00000042421	1110065P20Rik	53	-16.76
miR-193b-3p	ENSRNOG00000018145	Crat	52	-12.32
miR-193b-3p	ENSRNOG00000013290	Nrip3	71	-23.59
miR-193b-3p	ENSRNOG00000006867	Etv1	91	-14.38
miR-193b-3p	ENSRNOG00000011440	Ccdc39	90	-21.73
miR-193b-3p	ENSRNOG00000015385	Pink1	83	-16.66
miR-193b-3p	ENSRNOG00000019718	Galnt15	72	-18.09
miR-193b-3p	ENSRNOG00000005775	Phf14	89	-14.75
miR-193b-3p	ENSRNOG00000007110	Ankrd6	62	-18.64
miR-193b-3p	ENSRNOG00000008922	Trim14	81	-18.15
miR-193b-3p	ENSRNOG00000014087	Kifc3	56	-39.62
miR-193b-3p	ENSRNOG00000018602	Camta1	97	-14.83
miR-193b-3p	ENSRNOG00000005673	Runx1t1	97	-12.65
miR-193b-3p	ENSRNOG00000009170	Dmxl2	91	-25.23
miR-193b-3p	ENSRNOG00000022325	Smc2	55	-22.95
miR-193b-3p	ENSRNOG00000005281	Stx16	72	-18.17
miR-193b-3p	ENSRNOG00000017297	Cab39	97	-11.93
miR-193b-3p	ENSRNOG00000024594	Fhdc1	86	-33.05
miR-193b-3p	ENSRNOG00000011254	Fam76a	63	-13.07
miR-193b-3p	ENSRNOG00000016364	Gba2	99	-12.51
miR-193b-3p	ENSRNOG00000015063	Dhodh	82	-23.58
miR-193b-3p	ENSRNOG00000018198	Dapk1	73	-13.65
miR-193b-3p	ENSRNOG00000003936	Pwwp2a	65	-15.74
miR-193b-3p	ENSRNOG00000052080	Camk2b	59	-20.44
miR-193b-3p	ENSRNOG00000014548	Nedd9	77	-13.41
miR-193b-3p	ENSRNOG00000027480	Tmem116	96	-16.36
miR-193b-3p	ENSRNOG00000004566	Arhgef15	57	-14.33
miR-193b-3p	ENSRNOG00000026415	Col14a1	72	-20.05
miR-193b-3p	ENSRNOG00000000248	Srsf2	99	-11.93
miR-193b-3p	ENSRNOG00000007697	C8a	70	-38.96
miR-193b-3p	ENSRNOG00000017285	Tab1	84	-15.24
miR-193b-3p	ENSRNOG00000006004	Phc2	55	-17.94
miR-193b-3p	ENSRNOG00000042189	Rab31	79	-14.09
miR-193b-3p	ENSRNOG00000021669	Mybl1	64	-15.41
miR-193b-3p	ENSRNOG00000066137	Tmem240	92	-14.66

miR-193b-3p	ENSRNOG00000013692	Abhd18	75	-36.65
miR-193b-3p	ENSRNOG00000010983	Otog	72	-19.17
miR-193b-3p	ENSRNOG00000010086	Plagl2	76	-13.1
miR-193b-3p	ENSRNOG00000047783	Tmem200a	95	-16.42
miR-193b-3p	ENSRNOG00000045621	Gatc	59	-18.31
miR-193b-3p	ENSRNOG00000050714	Islr2	52	-18.86
miR-193b-3p	ENSRNOG00000007430	Slx4ip	57	-15.8
miR-193b-3p	ENSRNOG00000053406	Gramd1c	81	-17.9
miR-193b-3p	ENSRNOG00000047526	Zfp526	74	-42.38
miR-193b-3p	ENSRNOG00000047867	Klhdc8b	62	-15.21
miR-193b-3p	ENSRNOG00000050884	AABR07058745.1	91	-227.22
miR-193b-3p	ENSRNOG00000049076	Vwc2	91	-20.28
miR-193b-3p	ENSRNOG00000047213	Gnpda1	84	-13.11
miR-193b-3p	ENSRNOG00000003603	Arhgap44	59	-15.02
miR-193b-3p	ENSRNOG00000047873	Sec22c	74	-14.78
miR-193b-3p	ENSRNOG00000050169	Pet100	64	-20.07
miR-193b-3p	ENSRNOG00000048242	Txlna	91	-57.7
miR-193b-3p	ENSRNOG00000046211	Fbxw4	83	-17.53
miR-193b-3p	ENSRNOG00000050223	Rin1	74	-20.87
miR-193b-3p	ENSRNOG00000048891	Prr14l	93	-25.78
miR-193b-3p	ENSRNOG00000050282	Srcap	84	-28.51
miR-193b-3p	ENSRNOG00000050052	Cox19	92	-33.29
miR-193b-3p	ENSRNOG00000049426	Mmab	87	-41.89
miR-193b-3p	ENSRNOG00000047573	Ttc39a	76	-12.89
miR-193b-3p	ENSRNOG00000049179	Pidd1	86	-17.62
miR-193b-3p	ENSRNOG00000033693	AC135826.1	68	-14.19
miR-193b-3p	ENSRNOG00000047781	Slc25a23	53	-12.74
miR-193b-3p	ENSRNOG00000045846	Rfx2	64	-19.03
miR-193b-3p	ENSRNOG00000049053	Glipr1l2	99	-12.84
miR-193b-3p	ENSRNOG00000031058	Was	78	-15.07
miR-193b-3p	ENSRNOG00000049223	Vipas39	97	-20.53
miR-193b-3p	ENSRNOG00000017067	Haus1	95	-13.72
miR-193b-3p	ENSRNOG00000046744	Ankrd29	68	-14.68
miR-193b-3p	ENSRNOG00000050258	Ccnd3	74	-18.19
miR-193b-3p	ENSRNOG00000049326	Tmem234	98	-12.75
miR-193b-3p	ENSRNOG00000046307	Glyctk	71	-23.63
miR-193b-3p	ENSRNOG00000002462	B9d1	90	-19.93
miR-193b-3p	ENSRNOG00000050766	Rd3	50	-46.14
miR-193b-3p	ENSRNOG00000049758	Tbc1d16	87	-15.22
miR-193b-3p	ENSRNOG00000047295	Prr22	75	-24.42
miR-193b-3p	ENSRNOG00000068220	Ctdsp2	91	-20.1
miR-193b-3p	ENSRNOG00000050090	Slc6a17	54	-13.97
miR-193b-3p	ENSRNOG00000046204	Tspan15	59	-15.19
miR-193b-3p	ENSRNOG00000048441	Wdr82	82	-24.38
miR-193b-3p	ENSRNOG00000042333	Dnal1	53	-13.84
miR-193b-3p	ENSRNOG00000020441	Vps25	55	-19.72
miR-193b-3p	ENSRNOG00000009955	Plch1	61	-46.32
miR-193b-3p	ENSRNOG00000003009	AABR07039336.1	79	-12.74
miR-193b-3p	ENSRNOG00000007445	Asph	86	-43.37
miR-193b-3p	ENSRNOG00000000847	Csnk2b	95	-14.39
miR-193b-3p	ENSRNOG00000016810	Stmn1	90	-17.91
miR-193b-3p	ENSRNOG00000023430	AABR07014573.2	99	-11.93
miR-193b-3p	ENSRNOG00000054218	Il18rap	65	-14.53
miR-193b-3p	ENSRNOG00000017159	Dsc3	88	-18.24
miR-193b-3p	ENSRNOG00000001039	Eif2b1	95	-12.51
miR-193b-3p	ENSRNOG00000010484	Zdhhc21	86	-39.72
miR-193b-3p	ENSRNOG00000042576	Tcp11l1	57	-24.58
miR-193b-3p	ENSRNOG00000013930	Rnf4	69	-17.76
miR-193b-3p	ENSRNOG00000058975	Iqsec2	55	-13.58
miR-193b-3p	ENSRNOG00000060193	Prr29	50	-36.67
miR-193b-3p	ENSRNOG00000003699	Endov	53	-18.8

miR-193b-3p	ENSRNOG00000006967	Xiap	84	-17.75
miR-193b-3p	ENSRNOG00000009196	Rc3h2	82	-18.4
miR-193b-3p	ENSRNOG00000038597	Dlg1	84	-17.5
miR-193b-3p	ENSRNOG00000053015	Ccdc189	63	-15.69
miR-193b-3p	ENSRNOG00000022141	Ctdspl2	99	-14.44
miR-193b-3p	ENSRNOG00000051688	Syt15	86	-11.72
miR-193b-3p	ENSRNOG00000011986	Krt12	92	-16.67
miR-193b-3p	ENSRNOG00000002506	Cdkl2	65	-29.38
miR-193b-3p	ENSRNOG00000061876	Aldh4a1	57	-46.29
miR-193b-3p	ENSRNOG00000047218	Clic5	52	-22.62
miR-193b-3p	ENSRNOG00000005144	Tmem18	83	-17.16
miR-193b-3p	ENSRNOG00000052469	Bmpr1a	86	-24.33
miR-193b-3p	ENSRNOG00000047581	AABR07066700.1	58	-24.51
miR-193b-3p	ENSRNOG00000015423	Ccna2	89	-13.72
miR-193b-3p	ENSRNOG00000058478	Mapk8ip1	93	-12.51
miR-193b-3p	ENSRNOG00000061714	Slc14a2	75	-14.27
miR-193b-3p	ENSRNOG00000060137	AABR07037489.1	92	-16.11
miR-193b-3p	ENSRNOG00000052486	Kcna6	86	-51.87
miR-193b-3p	ENSRNOG00000004863	Mpped2	59	-12.88
miR-193b-3p	ENSRNOG00000007645	Kcnj9	95	-13
miR-193b-3p	ENSRNOG00000008195	Ywhaz	76	-20.62
miR-193b-3p	ENSRNOG00000018198	Dapk1	80	-13.65
miR-193b-3p	ENSRNOG00000000614	Bicc1	92	-15.08
miR-193b-3p	ENSRNOG00000059008	Socs7	51	-15.31
miR-193b-3p	ENSRNOG00000015835	Cacna2d2	66	-12.32
miR-193b-3p	ENSRNOG00000057180	Gpx5	59	-15.78
miR-193b-3p	ENSRNOG00000053047	Top2a	87	-13.99
miR-193b-3p	ENSRNOG00000054203	Sim2	68	-17.84
miR-193b-3p	ENSRNOG00000005287	Syne3	69	-14.46
miR-193b-3p	ENSRNOG00000055714	Ak7	95	-19.64
miR-193b-3p	ENSRNOG00000054757	Adcy6	92	-20.21
miR-193b-3p	ENSRNOG00000000924	Slc7a1	65	-42.92
miR-193b-3p	ENSRNOG00000055226	Dab2ip	58	-18.21
miR-193b-3p	ENSRNOG00000058136	Vom1r90	83	-23.44
miR-193b-3p	ENSRNOG00000031440	Nova1	86	-12.17
miR-193b-3p	ENSRNOG00000014903	Zfyve27	67	-45.43
miR-193b-3p	ENSRNOG00000004566	Arhgef15	56	-14.33
miR-193b-3p	ENSRNOG00000051977	Mmrn2	78	-17
miR-193b-3p	ENSRNOG00000054954	Lilrb2	97	-16.36
miR-193b-3p	ENSRNOG00000001047	Map2k7	72	-16.74
miR-193b-3p	ENSRNOG00000014981	Usp14	96	-17.14
miR-193b-3p	ENSRNOG00000051970	Aqp5	75	-23.61
miR-193b-3p	ENSRNOG00000051372	Mycn	86	-23.11
miR-193b-3p	ENSRNOG00000053047	Top2a	83	-13.99
miR-193b-3p	ENSRNOG00000053347	Ccdc28a	89	-14.04
miR-193b-3p	ENSRNOG00000057769	Oas1h	51	-19.75
miR-193b-3p	ENSRNOG00000008510	Abtb2	91	-14.75
miR-193b-3p	ENSRNOG00000018644	Slc6a7	89	-26.19
miR-193b-3p	ENSRNOG00000014668	RGD621098	96	-38.77
miR-193b-3p	ENSRNOG00000060141	Epdr1	91	-20.52
miR-193b-3p	ENSRNOG00000058057	AABR07019403.1	62	-20.91
miR-193b-3p	ENSRNOG00000017311	Me3	89	-14.41
miR-193b-3p	ENSRNOG00000058626	Kcnh8	92	-22.89
miR-193b-3p	ENSRNOG00000053725	Usf2	70	-11.98
miR-193b-3p	ENSRNOG00000013867	Fgf1	79	-38.14
miR-193b-3p	ENSRNOG00000051690	Clec9a	56	-19.75
miR-193b-3p	ENSRNOG00000061543	Ap2b1	71	-53.51
miR-193b-3p	ENSRNOG00000056209	Exo1	94	-11.93
miR-193b-3p	ENSRNOG00000058898	Nedd4	62	-14.79
miR-193b-3p	ENSRNOG00000013076	Csnk1e	81	-20.74
miR-193b-3p	ENSRNOG00000067655	Aldoa	72	-35.72

miR-193b-3p	ENSRNOG00000061424	Map3k3	65	-14.2
miR-193b-3p	ENSRNOG00000014811	Cyhr1	64	-19.91
miR-193b-3p	ENSRNOG00000061012	Kat5	94	-20.81
miR-193b-3p	ENSRNOG00000053923	Thap12	89	-16.91
miR-193b-3p	ENSRNOG00000057753	Nup62cl	88	-18.23
miR-193b-3p	ENSRNOG00000060571	Peds1	59	-14.74
miR-193b-3p	ENSRNOG00000059550	Ppp1r8	65	-20.88
miR-193b-3p	ENSRNOG00000054033	Adap1	94	-14.4
miR-193b-3p	ENSRNOG00000059919	Mtg2	69	-24.52
miR-193b-3p	ENSRNOG00000058870	Mroh7	75	-16.74
miR-193b-3p	ENSRNOG00000021758	Tnpo3	64	-16.78
miR-193b-3p	ENSRNOG00000053303	Bend4	87	-20.19
miR-193b-3p	ENSRNOG00000012803	Wdr61	74	-18.92
miR-193b-3p	ENSRNOG00000060571	Peds1	63	-14.74
miR-193b-3p	ENSRNOG00000054901	Rif1	99	-14
miR-193b-3p	ENSRNOG00000060665	Afap1	66	-24.24
miR-193b-3p	ENSRNOG00000016031	Bicd2	79	-52.48
miR-193b-3p	ENSRNOG00000053659	RGD1311703	96	-13.41
miR-193b-3p	ENSRNOG00000053232	Ror2	58	-15.39
miR-193b-3p	ENSRNOG00000001055	Rilpl1	73	-16.72
miR-193b-3p	ENSRNOG00000053210	Zc3h11a	65	-26.3
miR-193b-3p	ENSRNOG00000053428	Ccpg1	74	-22.88
miR-193b-3p	ENSRNOG00000059474	Mob1a	76	-16.09
miR-193b-3p	ENSRNOG00000019473	Dcun1d2	96	-13.16
miR-193b-3p	ENSRNOG00000059615	Exoc6b	62	-21.07
miR-193b-3p	ENSRNOG00000054286	Rrm2	73	-18.42
miR-193b-3p	ENSRNOG00000058202	Ppp2r2c	75	-16.9
miR-193b-3p	ENSRNOG00000021184	Mark2	54	-14.09
miR-193b-3p	ENSRNOG00000018191	Oprm1	75	-16.39
miR-193b-3p	ENSRNOG00000053285	Mllt6	89	-23.6
miR-193b-3p	ENSRNOG00000018320	Rps15a	54	-23.28
miR-193b-3p	ENSRNOG00000001050	Eif2ak1	80	-15.2
miR-193b-3p	ENSRNOG00000017553	Mief1	51	-16.36
miR-193b-3p	ENSRNOG00000018602	Camta1	91	-14.83
miR-193b-3p	ENSRNOG00000012726	Zbtb5	76	-49.88
miR-193b-3p	ENSRNOG00000006104	Tg	79	-53.47
miR-193b-3p	ENSRNOG00000013692	Abhd18	54	-36.65
miR-193b-3p	ENSRNOG00000018818	Txnl1	79	-14.34
miR-193b-3p	ENSRNOG00000020643	Bub3	83	-14.71
miR-193b-3p	ENSRNOG00000012379	Wdr18	77	-16.37
miR-193b-3p	ENSRNOG00000005310	Cul1	61	-16.77
miR-193b-3p	ENSRNOG00000002069	Slc35a5	95	-16.36
miR-193b-3p	ENSRNOG00000019281	Glmp	62	-14.5
miR-193b-3p	ENSRNOG00000047526	Zfp526	85	-29.09
miR-193b-3p	ENSRNOG00000070828	Fbxl14	59	-15.29
miR-193b-3p	ENSRNOG00000021828	Coro1b	80	-18.76
miR-193b-3p	ENSRNOG00000001774	Lrch3	90	-26.96
miR-193b-3p	ENSRNOG00000012818	Ksr1	54	-48.28
miR-193b-3p	ENSRNOG00000014874	Zfyve28	64	-13.67
miR-193b-3p	ENSRNOG00000018411	Pold3	64	-16.82
miR-193b-3p	ENSRNOG00000017017	Zbtb43	91	-36.59
miR-193b-3p	ENSRNOG00000045728	Crybg3	89	-56.08
miR-193b-3p	ENSRNOG00000069187	Inip	99	-13.91
miR-193b-3p	ENSRNOG00000004328	Deptor	55	-15.17
miR-193b-3p	ENSRNOG00000008748	Pex2	52	-12.93
miR-193b-3p	ENSRNOG00000015594	Rftn2	59	-23.53
miR-193b-3p	ENSRNOG00000019464	Ttc9c	65	-32.53
miR-193b-3p	ENSRNOG00000021615	Mettl26	87	-15.86
miR-193b-3p	ENSRNOG00000002238	Sema5b	52	-18.07
miR-193b-3p	ENSRNOG00000064511	Ankfn1	89	-14.03
miR-193b-3p	ENSRNOG00000011491	Dnajc13	94	-14.14

miR-193b-3p	ENSRNOG00000063253	Adgrl2	84	-12
miR-193b-3p	ENSRNOG00000019132	Fv1	81	-21.65
miR-193b-3p	ENSRNOG00000016468	Tpmt	99	-12.51
miR-193b-3p	ENSRNOG00000058898	Nedd4	72	-14.79
miR-193b-3p	ENSRNOG00000063660	Rpp14	96	-16.73
miR-193b-3p	ENSRNOG00000024801	Slc35a2	83	-25.02
miR-193b-3p	ENSRNOG00000053347	Ccdc28a	88	-14.04
miR-193b-3p	ENSRNOG00000026527	Snx32	73	-17.5
miR-193b-3p	ENSRNOG00000067320	Vom2r29	99	-18.06
miR-193b-3p	ENSRNOG00000016620	Csnk1g1	51	-26.42
miR-193b-3p	ENSRNOG00000026994	Afg3l1	61	-34.96
miR-193b-3p	ENSRNOG00000015143	Siah1	87	-11.96
miR-193b-3p	ENSRNOG00000052486	Kcna6	82	-51.87
miR-193b-3p	ENSRNOG00000002473	Odr4	74	-15.59
miR-193b-3p	ENSRNOG00000003955	Spata7	93	-15.33
miR-193b-3p	ENSRNOG00000066694	Mrtfb	76	-13.15
miR-193b-3p	ENSRNOG00000063819	Olr1686	89	-13
miR-193b-3p	ENSRNOG00000010303	Trim32	82	-21.78
miR-193b-3p	ENSRNOG00000064654	Olr677	82	-14.67
miR-193b-3p	ENSRNOG00000023322	Ostc	81	-19.4
miR-193b-3p	ENSRNOG00000012840	Sparc	70	-27.64
miR-193b-3p	ENSRNOG00000053015	Ccdc189	61	-15.69
miR-193b-3p	ENSRNOG00000021437	AABR07073181.1	57	-16.6
miR-193b-3p	ENSRNOG00000062572	Olr531	78	-18.87
miR-193b-3p	ENSRNOG00000023657	Gprin3	84	-12.51
miR-193b-3p	ENSRNOG00000001055	Rilpl1	98	-33.23
miR-193b-3p	ENSRNOG00000062731	Diras2	56	-37.36
miR-193b-3p	ENSRNOG00000063660	Rpp14	98	-16.73
miR-193b-3p	ENSRNOG00000018988	Ing5	89	-37.94
miR-193b-3p	ENSRNOG00000070578	Vom2r50	85	-21.79
miR-193b-3p	ENSRNOG00000000164	Lamp2	91	-19.55
miR-193b-3p	ENSRNOG00000000985	Cpsf4	70	-16.78
miR-193b-3p	ENSRNOG00000062383	Dio2	91	-39.85
miR-193b-3p	ENSRNOG00000000488	Hmga1	93	-23.66
miR-193b-3p	ENSRNOG00000016778	Cops7a	92	-24.26
miR-193b-3p	ENSRNOG00000006412	Zhx1	82	-22.89
miR-193b-3p	ENSRNOG00000024801	Slc35a2	76	-25.02
miR-193b-3p	ENSRNOG00000009149	Jam3	99	-17.3
miR-193b-3p	ENSRNOG00000005818	Elapor2	70	-15.46
miR-193b-3p	ENSRNOG00000003594	Tmem183a	94	-16.27
miR-193b-3p	ENSRNOG00000064294	Mdfic2	66	-18.96
miR-193b-3p	ENSRNOG00000003275	Spata32	82	-18.86
miR-193b-3p	ENSRNOG00000065733	Olr756	92	-14.02
miR-193b-3p	ENSRNOG00000057116	Pex19	72	-22.88
miR-193b-3p	ENSRNOG00000062777	Rsrc1	98	-11.93
miR-193b-3p	ENSRNOG00000026742	Ypel5	74	-27.06
miR-193b-3p	ENSRNOG00000013934	Dennd2b	71	-24.36
miR-193b-3p	ENSRNOG00000047867	Klhdc8b	70	-15.21
miR-193b-3p	ENSRNOG00000009149	Jam3	98	-39.1
miR-193b-3p	ENSRNOG00000001585	Nrip1	78	-11.96
miR-193b-3p	ENSRNOG00000005195	Cst3	52	-17.71
miR-193b-3p	ENSRNOG00000007666	Cacnb4	52	-21.74
miR-193b-3p	ENSRNOG00000033623	Pigx	81	-12.16
miR-193b-3p	ENSRNOG00000015063	Dhodh	81	-23.58
miR-193b-3p	ENSRNOG00000064933	Kcnj2	97	-23.51
miR-193b-3p	ENSRNOG00000003554	Piga	73	-16.63
miR-193b-3p	ENSRNOG00000004305	Abl2	83	-22.76
miR-193b-3p	ENSRNOG00000007604	Igsf8	79	-15.33
miR-193b-3p	ENSRNOG00000026085	Rabl3	92	-14.34
miR-193b-3p	ENSRNOG00000014722	Raph1	77	-19.59
miR-193b-3p	ENSRNOG00000062890	Zfp618	57	-19.28

miR-193b-3p	ENSRNOG00000003508	Ergic1	56	-17.7
miR-193b-3p	ENSRNOG00000016677	Csnk1g3	87	-13.83
miR-193b-3p	ENSRNOG00000042449	Cdc42se2	78	-13.21
miR-193b-3p	ENSRNOG00000004973	Ppp2r5c	94	-15.29
miR-193b-3p	ENSRNOG00000062809	RT1-M10-1	88	-48.29
miR-193b-3p	ENSRNOG00000004269	Myt1l	67	-14.14
miR-193b-3p	ENSRNOG00000008725	Slc37a3	91	-26.93
miR-193b-3p	ENSRNOG00000006311	Ccr9	75	-33.68
miR-193b-3p	ENSRNOG00000018829	Rnasek	63	-27.45
miR-193b-3p	ENSRNOG00000050044	Ptp4a2	96	-16.4
miR-193b-3p	ENSRNOG00000068444	Kcnj10	55	-30.48
miR-193b-3p	ENSRNOG00000042195	Pabpn1	81	-19.91
miR-193b-3p	ENSRNOG00000020269	Sugp2	51	-20.22
miR-193b-3p	ENSRNOG00000023657	Gprin3	85	-12.51
miR-193b-3p	ENSRNOG00000023870	Sh2d7	98	-18.37
miR-193b-3p	ENSRNOG00000007131	Paxip1	80	-21.78
miR-193b-3p	ENSRNOG00000021259	Prnp	98	-17.09
miR-193b-3p	ENSRNOG00000027503	Rps25	58	-21.23
miR-193b-3p	ENSRNOG00000014087	Kifc3	53	-39.62
miR-193b-3p	ENSRNOG00000026900	Ppil2	77	-11.96
miR-193b-3p	ENSRNOG00000001989	Alcam	57	-19.36
miR-193b-3p	ENSRNOG00000052486	Kcna6	88	-51.87
miR-193b-3p	ENSRNOG00000018988	Ing5	90	-37.94
miR-193b-3p	ENSRNOG00000063640	Bri3bp	95	-34.69
miR-193b-3p	ENSRNOG00000001047	Map2k7	61	-16.74
miR-193b-3p	ENSRNOG00000059540	Gcnt3	92	-35.03
miR-193b-3p	ENSRNOG00000011157	Jak1	60	-17.83
miR-193b-3p	ENSRNOG00000068220	Ctdsp2	91	-39.92
miR-193b-3p	ENSRNOG00000040281	Zc3h10	66	-16.94
miR-193b-3p	ENSRNOG00000032437	Pard3	50	-14.73
miR-193b-3p	ENSRNOG00000009481	Ddhd1	57	-16.33
miR-193b-3p	ENSRNOG00000020904	Cdc42ep2	63	-19.76
miR-193b-3p	ENSRNOG00000047867	Klhdc8b	64	-15.21
miR-193b-3p	ENSRNOG00000007280	Naa60	96	-16.37
miR-193b-3p	ENSRNOG00000027988	Zhx3	68	-68.54
miR-193b-3p	ENSRNOG00000036676	Csnk1d	61	-24.04
miR-193b-3p	ENSRNOG00000002021	Son	81	-24.21
miR-193b-3p	ENSRNOG00000064764	Kbtbd7	59	-27.09
miR-193b-3p	ENSRNOG00000009512	Esd	63	-15
miR-193b-3p	ENSRNOG00000021174	Macrod1	60	-12.77
miR-193b-3p	ENSRNOG00000014493	Golga1	50	-22.77
miR-193b-3p	ENSRNOG00000015143	Siah1	94	-11.96
miR-193b-3p	ENSRNOG00000018457	Ptpa	56	-31.39
miR-193b-3p	ENSRNOG00000010600	Cysrt1	60	-45.95
miR-193b-3p	ENSRNOG00000008922	Trim14	91	-18.15
miR-193b-3p	ENSRNOG00000026085	Rabl3	97	-14.34
miR-193b-3p	ENSRNOG00000053881	Baz2a	86	-18.4
miR-193b-3p	ENSRNOG00000000568	Slc29a3	94	-13.21
miR-193b-3p	ENSRNOG00000069117	Mmgt2	51	-16.17
miR-193b-3p	ENSRNOG00000022593	Pdpr	51	-47
miR-193b-3p	ENSRNOG00000018371	Tubb6	68	-40.15
miR-193b-3p	ENSRNOG00000028062	Arpc5	81	-14.03
miR-193b-3p	ENSRNOG00000014521	Armc8	88	-21.07
miR-193b-3p	ENSRNOG00000017775	Slc5a1	89	-21.28
miR-193b-3p	ENSRNOG00000017017	Zbtb43	76	-36.59
miR-193b-3p	ENSRNOG00000065635	Olfr1258	88	-13.41
miR-193b-3p	ENSRNOG00000049511	Safb2	59	-52.18
miR-193b-3p	ENSRNOG00000014520	Ing1	96	-16.27
miR-193b-3p	ENSRNOG00000067810	Olr287	96	-41.7
miR-193b-3p	ENSRNOG00000008427	Necap2	74	-44.83
miR-193b-3p	ENSRNOG00000019630	Hacl1	50	-16.18

miR-193b-3p	ENSRNOG00000071009	Kif19b	65	-45.94
miR-193b-3p	ENSRNOG00000064119	Timm8a1	97	-17.83
miR-193b-3p	ENSRNOG00000015830	Ano9	50	-39.12
miR-193b-3p	ENSRNOG00000014274	Trit1	79	-12.54
miR-193b-3p	ENSRNOG00000042560	Bag4	99	-15.2
miR-193b-3p	ENSRNOG00000063286	Olr753	91	-17.7
miR-193b-3p	ENSRNOG00000070914	Tmem238	52	-16.39
miR-193b-3p	ENSRNOG00000043182	Septin6	73	-17.02
miR-193b-3p	ENSRNOG00000049780	Sri	60	-17.28
miR-193b-3p	ENSRNOG00000012879	Fabp3	85	-21.8
miR-193b-3p	ENSRNOG00000068418	Gbp6	78	-21.79
miR-193b-3p	ENSRNOG00000065168	RGD1566007	71	-41.86
miR-193b-3p	ENSRNOG00000031824	Slc44a2	92	-19.09
miR-193b-3p	ENSRNOG00000005589	Dhrs7	79	-12.95
miR-193b-3p	ENSRNOG00000002278	Tec	68	-20.09
miR-193b-3p	ENSRNOG00000008966	Slco5a1	97	-12.51
miR-193b-3p	ENSRNOG00000063678	Mcl1	66	-19.15
miR-193b-3p	ENSRNOG00000007722	Tlcd3a	58	-34.01
miR-193b-3p	ENSRNOG00000049988	Mbtd1	77	-18.69
miR-193b-3p	ENSRNOG00000033568	Mapk8ip3	61	-58.34
miR-193b-3p	ENSRNOG00000024101	Phkb	50	-29.08
miR-193b-3p	ENSRNOG00000012818	Ksr1	54	-11.96
miR-193b-3p	ENSRNOG00000018778	Cadm1	85	-13.99
miR-193b-3p	ENSRNOG00000004367	Elk3	82	-23.18
miR-193b-3p	ENSRNOG00000024863	Fam76b	81	-18
miR-193b-3p	ENSRNOG00000070835	Ndufc1	96	-14.82
miR-193b-3p	ENSRNOG00000068418	Gbp6	52	-70.34
miR-193b-3p	ENSRNOG00000015669	Kctd11	96	-14.84
miR-193b-3p	ENSRNOG00000014454	Ap1m1	56	-17.94
miR-193b-3p	ENSRNOG00000010378	Slc4a5	61	-16.39
miR-193b-3p	ENSRNOG00000016913	Stk36	70	-15.71
miR-193b-3p	ENSRNOG00000016372	Slc12a7	83	-21.18
miR-193b-3p	ENSRNOG00000010109	Nol9	50	-19.28
miR-193b-3p	ENSRNOG00000004118	Frmpd4	68	-16.68
miR-193b-3p	ENSRNOG00000051915	Spred3	90	-35.1
miR-193b-3p	ENSRNOG00000065741	Il7r	80	-15.95
miR-193b-3p	ENSRNOG00000013267	Helz2	63	-20.49
miR-193b-3p	ENSRNOG00000019461	Zbtb3	71	-13.94
miR-193b-3p	ENSRNOG00000054460	Khdc3	73	-26.06
miR-193b-3p	ENSRNOG00000022066	Fam117b	58	-14.61
miR-193b-3p	ENSRNOG00000054549	Lss	95	-15.19
miR-193b-3p	ENSRNOG00000060141	Epdr1	89	-20.52
miR-193b-3p	ENSRNOG00000064275	Grpel2	67	-18.12
miR-193b-3p	ENSRNOG00000051948	Hcfc1	50	-12.84
miR-193b-3p	ENSRNOG00000027459	Zbtb45	76	-22.77
miR-193b-3p	ENSRNOG00000019634	Eif4e2	53	-28.19
miR-193b-3p	ENSRNOG00000032183	Scmh1	53	-22.84
miR-193b-3p	ENSRNOG00000064275	Grpel2	65	-18.12
miR-193b-3p	ENSRNOG00000068955	Rps13	92	-15.38
miR-193b-3p	ENSRNOG00000001593	Lnpk	71	-19.6
miR-193b-3p	ENSRNOG00000027770	Trpm3	67	-21.1
miR-193b-3p	ENSRNOG00000017692	Ppp1r37	65	-16.78
miR-193b-3p	ENSRNOG00000007333	Wdr20	68	-13.99
miR-193b-3p	ENSRNOG00000001868	Crkl	63	-14
miR-193b-3p	ENSRNOG00000005551	Derl1	79	-46.09
miR-193b-3p	ENSRNOG00000021475	Ldah	66	-17.34
miR-193b-3p	ENSRNOG00000013992	Arfrp1	54	-18.55
miR-193b-3p	ENSRNOG00000012803	Wdr61	56	-18.92
miR-193b-3p	ENSRNOG00000001055	Rilpl1	70	-16.72
miR-193b-3p	ENSRNOG00000062981	Smagp	71	-24.2
miR-193b-3p	ENSRNOG00000070548	Olr213	71	-19.94

miR-193b-3p	ENSRNOG00000004874	Flrt3	85	-16.15
miR-193b-3p	ENSRNOG00000016055	Fkrp	87	-16.67
miR-193b-3p	ENSRNOG00000010461	Gpx8	96	-17.91
miR-193b-3p	ENSRNOG00000065168	RGD1566007	57	-21.92
miR-193b-3p	ENSRNOG00000042576	Tcp11l1	55	-24.58
miR-193b-3p	ENSRNOG00000010239	Cnbp	69	-16.24
miR-193b-3p	ENSRNOG00000000247	Mfsd11	52	-24
miR-193b-3p	ENSRNOG00000013934	Dennd2b	70	-24.36
miR-193b-3p	ENSRNOG00000015143	Siah1	91	-11.96
miR-193b-3p	ENSRNOG00000063387	Olr310	72	-21.45
miR-193b-3p	ENSRNOG00000071101	Tmem127	54	-16.14
miR-193b-3p	ENSRNOG00000046445	Rcor3	60	-12.9
miR-193b-3p	ENSRNOG00000058733	Asap1	93	-17.85
miR-193b-3p	ENSRNOG00000046889	Dbi	89	-14.92
miR-193b-3p	ENSRNOG00000062741	Ldhal6b	71	-14.59
miR-193b-3p	ENSRNOG00000015474	Nelfa	62	-14.71
miR-193b-3p	ENSRNOG00000063808	Akain1	89	-20.98
miR-193b-3p	ENSRNOG00000003554	Piga	95	-40.64
miR-193b-3p	ENSRNOG00000064275	Grpel2	59	-18.12
miR-193b-3p	ENSRNOG00000018884	Ttc13	67	-14.17
miR-193b-3p	ENSRNOG00000016688	Ston1	70	-19.63
miR-193b-3p	ENSRNOG00000003108	Acbd4	71	-16.03
miR-193b-3p	ENSRNOG00000013267	Helz2	62	-20.49
miR-193b-3p	ENSRNOG00000063314	Utp23	64	-12.54
miR-193b-3p	ENSRNOG00000004328	Deptor	60	-15.17
miR-193b-3p	ENSRNOG00000022229	Sdad1	52	-14.53
miR-193b-3p	ENSRNOG00000068382	Olr1407	58	-12.9
miR-193b-3p	ENSRNOG00000028062	Arpc5	80	-14.03
miR-193b-3p	ENSRNOG00000008696	Nopchap1	58	-15.8
miR-193b-3p	ENSRNOG00000022686	Zdhhc2	90	-19.87
miR-193b-3p	ENSRNOG00000068444	Kcnj10	52	-30.48
miR-193b-3p	ENSRNOG00000059919	Mtg2	78	-24.52
miR-193b-3p	ENSRNOG00000063660	Rpp14	97	-16.73
miR-193b-3p	ENSRNOG00000013149	Vps33b	74	-16.03
miR-193b-3p	ENSRNOG00000012630	Rhoc	72	-19
miR-193b-3p	ENSRNOG00000014520	Ing1	94	-16.27

Table S5 Target genes of miR-346

miRNA ID	Gene ID	Gene Symbol	TargetScan_score	miranda_Energy
miR-346	ENSRNOG00000027731	Ubxn10	56	-20.15
miR-346	ENSRNOG00000000185	Mpst	93	-29.09
miR-346	ENSRNOG00000000456	Psmb8	68	-40.74
miR-346	ENSRNOG00000022289	Aip	88	-24.45
miR-346	ENSRNOG00000000556	Nodal	97	-19.27
miR-346	ENSRNOG00000000641	Nrbf2	97	-18.36
miR-346	ENSRNOG00000000704	Cmklr1	97	-19.67
miR-346	ENSRNOG00000000804	Mrps18b	82	-16.98
miR-346	ENSRNOG00000000815	Smpdl3a	97	-21.19
miR-346	ENSRNOG00000000827	Ier3	82	-16.53
miR-346	ENSRNOG00000029682	Clic1	88	-24.37
miR-346	ENSRNOG00000000885	Auts2l1	60	-22.98
miR-346	ENSRNOG00000000906	Medag	95	-47.46
miR-346	ENSRNOG00000001058	Timm44	92	-23.09
miR-346	ENSRNOG00000001135	Wsb2	56	-27.75
miR-346	ENSRNOG00000001142	Prkab1	70	-26
miR-346	ENSRNOG00000001163	Srsf9	89	-65.73
miR-346	ENSRNOG00000001191	Tchp	76	-43.15
miR-346	ENSRNOG00000001213	Aire	70	-30.99
miR-346	ENSRNOG00000001214	Pfkl	59	-17.58
miR-346	ENSRNOG00000001295	S100b	93	-37.78
miR-346	ENSRNOG00000001427	Orai2	91	-48.67
miR-346	ENSRNOG00000001544	Cyyr1	91	-72.03
miR-346	ENSRNOG00000001711	Plaat1	84	-36.48
miR-346	ENSRNOG00000001781	Lmln	92	-26.27
miR-346	ENSRNOG00000001843	Bcl6	97	-39.87
miR-346	ENSRNOG00000001844	Klhl42	82	-49.24
miR-346	ENSRNOG00000037909	Ppm1f	92	-21.91
miR-346	ENSRNOG00000001849	Mapk1	85	-78.69
miR-346	ENSRNOG00000001956	Dzip3	86	-20.85
miR-346	ENSRNOG00000002031	Naa11	86	-31.25
miR-346	ENSRNOG00000002085	Thegl	97	-33
miR-346	ENSRNOG00000002117	Barhl2	84	-24.75
miR-346	ENSRNOG00000002142	Cds1	52	-42.87
miR-346	ENSRNOG00000002200	AABR07072371.1	97	-18.36
miR-346	ENSRNOG00000002305	Slc15a2	87	-13.86
miR-346	ENSRNOG00000002414	Tfcp2l1	95	-41.31
miR-346	ENSRNOG00000002659	Ciita	90	-73.94
miR-346	ENSRNOG00000002695	Tfb2m	66	-20.93
miR-346	ENSRNOG00000002794	Selp	71	-22.89
miR-346	ENSRNOG00000002951	Ccnb3	99	-25.57
miR-346	ENSRNOG00000002823	Mapk9	82	-20.88
miR-346	ENSRNOG00000002930	Ppl	88	-22.04
miR-346	ENSRNOG00000024578	Ttyh2	58	-29.61
miR-346	ENSRNOG00000003122	Hdac8	90	-25.17
miR-346	ENSRNOG00000003254	Il23a	72	-22.51
miR-346	ENSRNOG00000003243	Wdr81	56	-20.52
miR-346	ENSRNOG00000003248	Mpzl1	65	-17.57
miR-346	ENSRNOG00000003183	Fmod	61	-42.8
miR-346	ENSRNOG00000003284	Epn3	91	-45.5
miR-346	ENSRNOG00000003365	Cadm3	96	-25.17
miR-346	ENSRNOG00000003611	Dynlt3	89	-20.14
miR-346	ENSRNOG00000003546	Tnfrsf12a	90	-54.35
miR-346	ENSRNOG00000003661	Jpt1	79	-45.05
miR-346	ENSRNOG00000003732	Flrt2	72	-17.49
miR-346	ENSRNOG00000003800	Rgs9	87	-19.58
miR-346	ENSRNOG00000003988	Dnajc27	71	-33.64
miR-346	ENSRNOG00000003979	Zranb3	67	-20.87
miR-346	ENSRNOG00000004033	Sema6a	63	-21.84

miR-346	ENSRNOG00000004132	Lasp1	52	-49.82
miR-346	ENSRNOG00000004195	Dtl	91	-18.85
miR-346	ENSRNOG00000004206	Glrx5	86	-44.41
miR-346	ENSRNOG00000004400	Avpr1a	99	-18.16
miR-346	ENSRNOG00000004424	Rabif	88	-20.21
miR-346	ENSRNOG00000004327	Ddc	50	-24.54
miR-346	ENSRNOG00000023176	Kidins220	67	-50.2
miR-346	ENSRNOG00000004317	Vipr2	59	-42.85
miR-346	ENSRNOG00000004411	Tspan8	92	-26.72
miR-346	ENSRNOG00000004417	Fam117a	84	-19.43
miR-346	ENSRNOG00000004524	Desi2	73	-20.07
miR-346	ENSRNOG00000004430	Cep131	60	-25.11
miR-346	ENSRNOG00000004330	Chrdl1	91	-31.74
miR-346	ENSRNOG00000004464	Sel1l	83	-21.66
miR-346	ENSRNOG00000004682	Parpbp	86	-37.72
miR-346	ENSRNOG00000004571	Cdin1	81	-27.83
miR-346	ENSRNOG00000008764	Otud5	65	-26.91
miR-346	ENSRNOG00000004751	Jkamp	86	-50.48
miR-346	ENSRNOG00000004201	Rims2	99	-19.4
miR-346	ENSRNOG00000004854	Has2	69	-15.37
miR-346	ENSRNOG00000004616	Npm1	76	-26.08
miR-346	ENSRNOG00000004342	Ahr	79	-21.19
miR-346	ENSRNOG00000004755	Commd9	93	-26.43
miR-346	ENSRNOG00000004756	Syt2	66	-23.41
miR-346	ENSRNOG00000005008	Angpt4	71	-23.19
miR-346	ENSRNOG00000005016	Tmed4	56	-20.74
miR-346	ENSRNOG00000004940	Rnf215	56	-51.35
miR-346	ENSRNOG00000005083	Rtfdc1	70	-42.85
miR-346	ENSRNOG00000005133	Mapt	57	-49.99
miR-346	ENSRNOG00000005093	Lgr6	52	-43.33
miR-346	ENSRNOG00000005248	Slc1a4	61	-24.16
miR-346	ENSRNOG00000005258	Myef2	80	-21.03
miR-346	ENSRNOG00000005353	Rybp	78	-17.12
miR-346	ENSRNOG00000005365	Kcnip1	66	-19.47
miR-346	ENSRNOG00000005260	Acp1	99	-21.53
miR-346	ENSRNOG00000005447	Ypel2	97	-44.37
miR-346	ENSRNOG00000005278	Trhde	88	-17.53
miR-346	ENSRNOG00000005331	Vapb	80	-19.43
miR-346	ENSRNOG00000005299	Kif5a	88	-62.37
miR-346	ENSRNOG00000005703	Arfgef1	69	-21.33
miR-346	ENSRNOG00000005781	Wnt16	84	-16.43
miR-346	ENSRNOG00000005955	Naalad2	99	-46.6
miR-346	ENSRNOG00000005853	Tacr1	87	-21.39
miR-346	ENSRNOG00000005309	Scn8a	61	-23.95
miR-346	ENSRNOG00000005927	Cpsf6	64	-32.29
miR-346	ENSRNOG00000005464	Lgalsl	89	-17.14
miR-346	ENSRNOG00000005736	Eif1ax	63	-18.36
miR-346	ENSRNOG00000006403	Nectin1	50	-30.91
miR-346	ENSRNOG00000006505	Borcs5	56	-44.88
miR-346	ENSRNOG00000006296	Six6	53	-16.05
miR-346	ENSRNOG00000006235	Nell2	68	-16.57
miR-346	ENSRNOG00000006727	Dtd2	98	-16.04
miR-346	ENSRNOG00000006979	Hpca	81	-22.28
miR-346	ENSRNOG00000006470	Camk1g	86	-22.6
miR-346	ENSRNOG00000007002	Lif	64	-52.94
miR-346	ENSRNOG00000007059	Atp1b4	66	-42.51
miR-346	ENSRNOG00000006965	Aff4	51	-16.01
miR-346	ENSRNOG00000007258	Sbspon	83	-19.82
miR-346	ENSRNOG00000007253	Cbll1	90	-17.61
miR-346	ENSRNOG00000006227	Ifih1	84	-28.66
miR-346	ENSRNOG00000007142	B3gat1	84	-23.53

miR-346	ENSRNOG00000007682	Gria3	84	-59.02
miR-346	ENSRNOG00000007882	Ablim2	62	-54.85
miR-346	ENSRNOG00000007660	Churc1	50	-26.54
miR-346	ENSRNOG00000008113	RGD1561149	96	-26.23
miR-346	ENSRNOG00000008118	Sync	74	-26.8
miR-346	ENSRNOG00000007710	Usp20	58	-17.43
miR-346	ENSRNOG00000008121	Slc13a1	80	-24.2
miR-346	ENSRNOG00000007393	Ndrg1	68	-19.06
miR-346	ENSRNOG00000008187	Ubash3b	66	-18.18
miR-346	ENSRNOG00000008264	Fndc8	50	-47.51
miR-346	ENSRNOG00000007982	Slc9a1	59	-22.41
miR-346	ENSRNOG00000008316	Vps39	63	-29.53
miR-346	ENSRNOG00000008591	Colec10	71	-19.89
miR-346	ENSRNOG00000008000	Syt13	51	-45.46
miR-346	ENSRNOG00000008478	Mmp13	98	-27.87
miR-346	ENSRNOG00000008676	Emp1	92	-43.75
miR-346	ENSRNOG00000008297	Oser1	66	-21.5
miR-346	ENSRNOG00000008543	Pdlim2	71	-24.61
miR-346	ENSRNOG00000008634	Cc2d1b	75	-20.61
miR-346	ENSRNOG00000022730	Zfp57	71	-21.84
miR-346	ENSRNOG00000008939	Nxph1	89	-43.56
miR-346	ENSRNOG00000009068	Phlda3	91	-24.82
miR-346	ENSRNOG00000008927	Hbp1	51	-15.33
miR-346	ENSRNOG00000008641	Gnpnat1	91	-19.02
miR-346	ENSRNOG00000037211	Kif14	99	-18.65
miR-346	ENSRNOG00000008815	Fbxo46	62	-84.95
miR-346	ENSRNOG00000008851	Slc35b4	85	-24.55
miR-346	ENSRNOG00000008872	Tmem68	90	-23.05
miR-346	ENSRNOG00000009166	Ddx24	56	-19.53
miR-346	ENSRNOG00000009094	Nudt4	83	-15.52
miR-346	ENSRNOG00000009316	Bmp10	80	-20.65
miR-346	ENSRNOG00000009326	Smcp	62	-23.73
miR-346	ENSRNOG00000009046	Phf13	70	-34.94
miR-346	ENSRNOG00000032178	Cenpa	60	-21.16
miR-346	ENSRNOG00000008862	Abcg4	91	-25.18
miR-346	ENSRNOG00000009076	Ttpal	79	-23.43
miR-346	ENSRNOG00000009425	Fgf7	95	-23.02
miR-346	ENSRNOG00000009649	Slitrk5	70	-19.83
miR-346	ENSRNOG00000009244	Abhd4	66	-20.08
miR-346	ENSRNOG00000009832	Slc39a14	55	-22.84
miR-346	ENSRNOG00000009636	Scrn1	75	-55.96
miR-346	ENSRNOG00000009910	Swap70	75	-20.27
miR-346	ENSRNOG00000010034	Asah1	86	-16.57
miR-346	ENSRNOG00000010091	Efcab14	91	-26.51
miR-346	ENSRNOG00000009934	Emc3	54	-21.73
miR-346	ENSRNOG00000010217	Prrc2b	66	-43.81
miR-346	ENSRNOG00000010083	Prpsap1	80	-22.32
miR-346	ENSRNOG00000010312	Bhlhe23	56	-25.5
miR-346	ENSRNOG00000010087	Zfp143	69	-18.42
miR-346	ENSRNOG00000009760	Palm	72	-28.86
miR-346	ENSRNOG00000009421	Ivd	65	-15.95
miR-346	ENSRNOG00000010415	Atxn7l1	75	-45.23
miR-346	ENSRNOG00000010053	Calcr	76	-22.3
miR-346	ENSRNOG00000010438	Cpt1b	87	-36.44
miR-346	ENSRNOG00000010553	Pnma1	54	-53.91
miR-346	ENSRNOG00000010538	Commd7	87	-19.61
miR-346	ENSRNOG00000010331	Ctsb	90	-75.29
miR-346	ENSRNOG00000010630	Prcp	55	-25.99
miR-346	ENSRNOG00000010750	Polr1f	89	-19.56
miR-346	ENSRNOG00000010555	Phyhip	58	-47.93
miR-346	ENSRNOG00000010379	Celf1	74	-18.26

miR-346	ENSRNOG00000010882	Sptlc1	74	-27.17
miR-346	ENSRNOG00000010914	Pigh	75	-50.22
miR-346	ENSRNOG00000011032	Lhfpl2	91	-29.21
miR-346	ENSRNOG00000009820	Bnip3l	75	-13.86
miR-346	ENSRNOG00000010898	Prok2	84	-34.45
miR-346	ENSRNOG00000011132	D430041D05Rik	78	-18.08
miR-346	ENSRNOG00000011320	Igfbpl1	74	-18.79
miR-346	ENSRNOG00000011363	Napepld	82	-18.49
miR-346	ENSRNOG00000011750	Fam180a	71	-21.63
miR-346	ENSRNOG00000011467	Uck1	78	-54.06
miR-346	ENSRNOG00000011863	Gins3	79	-20.21
miR-346	ENSRNOG00000011826	Lzts1	84	-74.09
miR-346	ENSRNOG00000011918	Vsx2	79	-23.1
miR-346	ENSRNOG00000011460	Arfgef3	78	-21.23
miR-346	ENSRNOG00000011991	Slc10a7	99	-17.73
miR-346	ENSRNOG00000046192	Nsun4	59	-19.31
miR-346	ENSRNOG00000011646	Rem2	71	-14.61
miR-346	ENSRNOG00000012192	Znf740	69	-27.43
miR-346	ENSRNOG00000046881	Pglyrp3b	87	-22.69
miR-346	ENSRNOG00000012054	Zmpste24	97	-19.82
miR-346	ENSRNOG00000012333	Kbtbd11	53	-43.86
miR-346	ENSRNOG00000012312	Prr9	82	-37.61
miR-346	ENSRNOG00000012343	Pdp2	98	-20.31
miR-346	ENSRNOG00000012184	Urgcp	93	-23.23
miR-346	ENSRNOG00000011548	Ebf2	97	-37.27
miR-346	ENSRNOG00000012083	St6galnac2	81	-51.16
miR-346	ENSRNOG00000012566	Kcnv2	52	-21.19
miR-346	ENSRNOG00000012719	Tdrd12	83	-20.16
miR-346	ENSRNOG00000012759	Col19a1	61	-22.01
miR-346	ENSRNOG00000012826	Creb3l2	61	-47.2
miR-346	ENSRNOG00000012965	Themis2	67	-23.07
miR-346	ENSRNOG00000013085	Nkain3	63	-20.54
miR-346	ENSRNOG00000012840	Sparc	84	-29.9
miR-346	ENSRNOG00000013005	Rpa2	93	-26
miR-346	ENSRNOG00000013118	Atox1	80	-19.93
miR-346	ENSRNOG00000012622	Mmp15	80	-23
miR-346	ENSRNOG00000013222	Cd207	97	-20.14
miR-346	ENSRNOG00000012815	Chst10	51	-44.96
miR-346	ENSRNOG00000013340	Twsg1	59	-16.94
miR-346	ENSRNOG00000012934	Arhgef7	50	-56.62
miR-346	ENSRNOG00000012929	Wsb1	99	-17.37
miR-346	ENSRNOG00000013565	Zfp507	63	-31.69
miR-346	ENSRNOG00000013661	Kif26a	82	-27.79
miR-346	ENSRNOG00000013719	Dph5	91	-49.31
miR-346	ENSRNOG00000049489	Rad54l2	85	-30.43
miR-346	ENSRNOG00000049498	Nat1	78	-27.29
miR-346	ENSRNOG00000023851	Igsf3	80	-48.75
miR-346	ENSRNOG00000014164	Paqr5	63	-23.05
miR-346	ENSRNOG00000014064	Ctsh	99	-27.7
miR-346	ENSRNOG00000014322	Kazn	76	-23.97
miR-346	ENSRNOG00000014363	Arhgef3	85	-23.58
miR-346	ENSRNOG00000014274	Trit1	96	-24.75
miR-346	ENSRNOG00000014603	Sgcg	98	-24.05
miR-346	ENSRNOG00000014619	Dhx38	96	-26.15
miR-346	ENSRNOG00000014723	Cbfa2t3	97	-16.04
miR-346	ENSRNOG00000014811	Cyhr1	85	-30.13
miR-346	ENSRNOG00000064866	Rnf183	86	-19.1
miR-346	ENSRNOG00000014218	Tmem25	60	-23.41
miR-346	ENSRNOG00000014857	RGD1560065	73	-18.76
miR-346	ENSRNOG00000014834	Epc1	99	-18.24
miR-346	ENSRNOG00000014659	Ebpl	79	-22.14

miR-346	ENSRNOG00000032246	Acsm3	81	-22.45
miR-346	ENSRNOG00000014801	Exog	82	-38.63
miR-346	ENSRNOG00000015027	Il18r1	93	-21.19
miR-346	ENSRNOG00000014338	Slc25a25	72	-21.52
miR-346	ENSRNOG00000015084	Necab2	78	-17.33
miR-346	ENSRNOG00000014992	M6pr	59	-59.55
miR-346	ENSRNOG00000015192	Mrps5	52	-26.44
miR-346	ENSRNOG00000014876	Lpin2	71	-42.99
miR-346	ENSRNOG00000015269	Atf7	93	-18.9
miR-346	ENSRNOG00000015191	Phc1	85	-20.43
miR-346	ENSRNOG00000015215	Cript	85	-51.92
miR-346	ENSRNOG00000015339	Shoc2	91	-16.86
miR-346	ENSRNOG00000015052	Star	88	-23.61
miR-346	ENSRNOG00000014785	Ykt6	80	-29.51
miR-346	ENSRNOG00000015409	Usp5	77	-25.03
miR-346	ENSRNOG00000015558	Zfp512b	92	-20.26
miR-346	ENSRNOG00000015417	Kansl3	66	-40.23
miR-346	ENSRNOG00000015441	Il4r	88	-23.37
miR-346	ENSRNOG00000015666	Abcc12	64	-17.96
miR-346	ENSRNOG00000015614	Ppp1r16b	86	-56.56
miR-346	ENSRNOG00000015591	Cndp2	96	-28.08
miR-346	ENSRNOG00000015928	Dhx35	94	-28.75
miR-346	ENSRNOG00000016091	Tmem169	75	-16.39
miR-346	ENSRNOG00000015977	Zfp609	81	-42.05
miR-346	ENSRNOG00000016161	Lman2	79	-17.67
miR-346	ENSRNOG00000016190	Coq9	89	-22.47
miR-346	ENSRNOG00000016108	Phlpp2	84	-44.5
miR-346	ENSRNOG00000016269	Rpl7l1	58	-80
miR-346	ENSRNOG00000016298	Lysmd3	59	-20.22
miR-346	ENSRNOG00000016412	Fxyd6	82	-20.27
miR-346	ENSRNOG00000016358	Apba2	67	-35.16
miR-346	ENSRNOG00000016574	Fiz1	95	-20.87
miR-346	ENSRNOG00000016374	Fgfr2	63	-23.41
miR-346	ENSRNOG00000016196	Dlgap1	71	-19.08
miR-346	ENSRNOG00000016405	Pcsk4	97	-27.85
miR-346	ENSRNOG00000016698	Rnmt	84	-16.14
miR-346	ENSRNOG00000016734	Emilin3	59	-22.03
miR-346	ENSRNOG00000016475	Nt5c3b	89	-22.3
miR-346	ENSRNOG00000016769	Rab38	87	-20.33
miR-346	ENSRNOG00000016538	Itga8	84	-23.3
miR-346	ENSRNOG00000016978	AI467606	86	-23.49
miR-346	ENSRNOG00000016783	Fshr	87	-20.23
miR-346	ENSRNOG00000017002	Adrb1	53	-41.76
miR-346	ENSRNOG00000017017	Zbtb43	81	-28.39
miR-346	ENSRNOG00000016780	RGD1310951	93	-28.05
miR-346	ENSRNOG00000017022	Cerk	88	-22.77
miR-346	ENSRNOG00000017078	Selenon	97	-47.72
miR-346	ENSRNOG00000017087	Man1c1	63	-20.36
miR-346	ENSRNOG00000027343	Ttc39d	66	-16.31
miR-346	ENSRNOG00000017194	Prdx1	98	-39.54
miR-346	ENSRNOG00000017210	Slc22a23	63	-16.79
miR-346	ENSRNOG00000017233	Mmachc	78	-14.04
miR-346	ENSRNOG00000017249	Zfp366	66	-21.78
miR-346	ENSRNOG00000017201	Cd2bp2	81	-21.09
miR-346	ENSRNOG00000017342	Zdhhc7	67	-17.66
miR-346	ENSRNOG00000017426	Rragc	76	-21.5
miR-346	ENSRNOG00000017206	Igfbp5	93	-23.9
miR-346	ENSRNOG00000017523	H6pd	87	-41.78
miR-346	ENSRNOG00000017403	Apobr	70	-19.3
miR-346	ENSRNOG00000017441	Tpm3	53	-29.55
miR-346	ENSRNOG00000017459	C1ql3	80	-22.76

miR-346	ENSRNOG00000017254	Nsun2	93	-41.82
miR-346	ENSRNOG00000017500	Mtss2	74	-57.37
miR-346	ENSRNOG00000017575	Gins2	72	-21.87
miR-346	ENSRNOG00000017684	Fbxl22	91	-26.29
miR-346	ENSRNOG00000017728	Edrf1	81	-25.6
miR-346	ENSRNOG00000017695	AABR07012795.1	99	-39.54
miR-346	ENSRNOG00000017791	Arhgap12	81	-16.27
miR-346	ENSRNOG00000017606	P2rx1	95	-27.77
miR-346	ENSRNOG00000017883	Camsap1	87	-17.9
miR-346	ENSRNOG00000032990	4931406B18Rik	95	-42.58
miR-346	ENSRNOG00000017816	Pde6a	89	-27.34
miR-346	ENSRNOG00000017773	Crispld1	94	-21.38
miR-346	ENSRNOG00000017974	Gprin1	69	-42.58
miR-346	ENSRNOG00000018012	Tulp4	56	-28.07
miR-346	ENSRNOG00000018159	Anxa4	80	-21.87
miR-346	ENSRNOG00000018027	Taf5l	81	-18
miR-346	ENSRNOG00000023317	Colgalt1	96	-26.91
miR-346	ENSRNOG00000018406	Wipf1	94	-65.48
miR-346	ENSRNOG00000018603	Carns1	66	-24.14
miR-346	ENSRNOG00000018570	C1qtnf3	99	-17.02
miR-346	ENSRNOG00000022101	Crabp2	65	-17.68
miR-346	ENSRNOG00000018655	Adsl	51	-38.77
miR-346	ENSRNOG00000018627	Plekhb1	94	-30.97
miR-346	ENSRNOG00000018708	Ppp1ca	87	-20.31
miR-346	ENSRNOG00000023172	Dnajb3	75	-18.37
miR-346	ENSRNOG00000018441	Ggt7	94	-46.68
miR-346	ENSRNOG00000018642	Leng8	75	-22.76
miR-346	ENSRNOG00000049442	AABR07026317.1	95	-24.24
miR-346	ENSRNOG00000018765	Pold4	95	-19.51
miR-346	ENSRNOG00000018971	Mob3a	78	-21.83
miR-346	ENSRNOG00000018980	Tjap1	68	-19.47
miR-346	ENSRNOG00000018835	Notch2	62	-19.71
miR-346	ENSRNOG00000019221	Gstm4	91	-18.32
miR-346	ENSRNOG00000019000	Limk2	92	-22.36
miR-346	ENSRNOG00000019235	Rbm22	70	-17.03
miR-346	ENSRNOG00000019288	Ogfod1	76	-55.93
miR-346	ENSRNOG00000019458	Shpk	81	-20
miR-346	ENSRNOG00000019590	Smg5	90	-112.69
miR-346	ENSRNOG00000019615	Colq	95	-26.39
miR-346	ENSRNOG00000019616	Prss8	94	-29.68
miR-346	ENSRNOG00000019529	Btbd1	91	-18.72
miR-346	ENSRNOG00000019689	Vwf	90	-20.66
miR-346	ENSRNOG00000019760	Oxnad1	77	-22.18
miR-346	ENSRNOG00000019825	Zdhhc24	72	-16.49
miR-346	ENSRNOG00000019848	Rbm39	98	-21.4
miR-346	ENSRNOG00000019955	Ogdhl	80	-16.57
miR-346	ENSRNOG00000020247	Cnih2	71	-22.38
miR-346	ENSRNOG00000020261	Fam53c	65	-42.23
miR-346	ENSRNOG00000020288	Slc25a20	97	-23.59
miR-346	ENSRNOG00000020336	Ezh1	64	-50.91
miR-346	ENSRNOG00000020588	Efna4	95	-29.29
miR-346	ENSRNOG00000020803	Meox1	80	-24.41
miR-346	ENSRNOG00000050994	Cttn	81	-25.2
miR-346	ENSRNOG00000020865	Ano1	73	-22.95
miR-346	ENSRNOG00000020975	Mrpl49	98	-27.06
miR-346	ENSRNOG00000020979	Psmb4	95	-24.12
miR-346	ENSRNOG00000021025	Ppp2r5b	83	-19.98
miR-346	ENSRNOG00000021085	Sf1	87	-21.54
miR-346	ENSRNOG00000021099	Lysmd1	76	-22.18
miR-346	ENSRNOG00000021100	Tnfaip8l2	57	-72.57
miR-346	ENSRNOG00000021108	Slc22a12	60	-49.84

miR-346	ENSRNOG00000021120	Prune1	98	-21.56
miR-346	ENSRNOG00000021137	Kctd15	98	-22.29
miR-346	ENSRNOG00000021274	Fermt1	57	-21.96
miR-346	ENSRNOG00000025273	AABR07068728.1	94	-18.19
miR-346	ENSRNOG00000021798	Pglyrp4	68	-21.98
miR-346	ENSRNOG00000025930	Chst13	89	-24.53
miR-346	ENSRNOG00000001448	Hip1	87	-45.63
miR-346	ENSRNOG00000022260	Senp1	62	-16.02
miR-346	ENSRNOG00000024364	Enkd1	81	-21.87
miR-346	ENSRNOG00000026217	Armc2	83	-47.58
miR-346	ENSRNOG00000027955	Zfp133	97	-52.09
miR-346	ENSRNOG00000023318	Tigd3	77	-17.24
miR-346	ENSRNOG00000023946	Dmac1	59	-22.58
miR-346	ENSRNOG00000013602	Usp48	52	-45.75
miR-346	ENSRNOG00000025724	Mtf1	86	-50.29
miR-346	ENSRNOG00000022483	Trim50	93	-20.09
miR-346	ENSRNOG00000024420	Eme1	83	-16.94
miR-346	ENSRNOG00000026647	Cxcl16	94	-24.97
miR-346	ENSRNOG00000021962	Fzd2	71	-24.86
miR-346	ENSRNOG00000012084	Xpnpep1	79	-21.58
miR-346	ENSRNOG00000006302	Gclc	89	-17.24
miR-346	ENSRNOG00000015554	Ankdd1a	82	-23.7
miR-346	ENSRNOG00000022136	BC016579	97	-28.1
miR-346	ENSRNOG00000027368	Ubald1	95	-23.33
miR-346	ENSRNOG00000026403	Naf1	99	-18.42
miR-346	ENSRNOG00000032884	Scn11a	58	-25.1
miR-346	ENSRNOG00000024796	Lrrc47	81	-27.11
miR-346	ENSRNOG00000002919	Gfap	57	-22.85
miR-346	ENSRNOG00000026748	Dennd2a	80	-27.02
miR-346	ENSRNOG00000024543	Mfsd4b	90	-28.01
miR-346	ENSRNOG00000011063	Dennd1b	77	-22.41
miR-346	ENSRNOG00000023238	Dgkd	72	-50
miR-346	ENSRNOG00000012735	Kif24	93	-26.8
miR-346	ENSRNOG00000023338	Tspan2	72	-32.31
miR-346	ENSRNOG00000026523	RGD1309106	91	-19.51
miR-346	ENSRNOG00000023993	Kif1a	56	-18.84
miR-346	ENSRNOG00000012442	Cemip	94	-19.69
miR-346	ENSRNOG00000000314	Sec63	52	-23.26
miR-346	ENSRNOG00000024825	Fam163a	53	-19.6
miR-346	ENSRNOG00000024930	Smim19	61	-61.13
miR-346	ENSRNOG00000026527	Snx32	75	-18.13
miR-346	ENSRNOG00000024221	Mettl24	95	-17.67
miR-346	ENSRNOG00000028075	Dnaaf8	89	-25.4
miR-346	ENSRNOG00000023937	Slc49a3	93	-24.19
miR-346	ENSRNOG00000028404	Ppp1r1b	73	-17.05
miR-346	ENSRNOG00000013265	Tgfbr2	70	-41.1
miR-346	ENSRNOG00000026053	Grem1	81	-19.32
miR-346	ENSRNOG00000026866	Syn3	50	-176.12
miR-346	ENSRNOG00000022421	Crtc1	64	-19.64
miR-346	ENSRNOG00000027859	Tmem26	98	-19.82
miR-346	ENSRNOG00000022732	Tmem126b	88	-65.81
miR-346	ENSRNOG00000022698	Vsig10	83	-30.96
miR-346	ENSRNOG00000025075	Relt	61	-23.55
miR-346	ENSRNOG00000027833	Fnip2	95	-24.21
miR-346	ENSRNOG00000006958	AC126572.1	84	-24.8
miR-346	ENSRNOG00000027873	RT1-M1-4	84	-23.65
miR-346	ENSRNOG00000023458	Dcaf12l1	93	-18.03
miR-346	ENSRNOG00000017215	RGD1308601	54	-21.26
miR-346	ENSRNOG00000059890	Clec2d2	81	-23.7
miR-346	ENSRNOG00000021573	Dpy19l3	56	-88.2
miR-346	ENSRNOG00000022030	Aunip	82	-29.63

miR-346	ENSRNOG00000027257	Gmip	76	-19.66
miR-346	ENSRNOG00000008620	Smad3	88	-51.56
miR-346	ENSRNOG00000032472	Adgrg2	89	-15.55
miR-346	ENSRNOG00000033192	Osmr	82	-39.21
miR-346	ENSRNOG00000033796	Kcnj5	93	-46.69
miR-346	ENSRNOG00000049590	RT1-M2	97	-24.67
miR-346	ENSRNOG00000043059	Aplf	73	-24.75
miR-346	ENSRNOG00000022261	Cenpu	72	-71.96
miR-346	ENSRNOG00000005633	Zbtb49	65	-22.43
miR-346	ENSRNOG00000000282	Gsc2	92	-28.65
miR-346	ENSRNOG00000031269	RGD1304694	71	-38.9
miR-346	ENSRNOG00000015666	Abcc12	72	-17.96
miR-346	ENSRNOG00000020703	Sipa1l3	50	-15.27
miR-346	ENSRNOG00000031688	Olr1073	68	-19.53
miR-346	ENSRNOG00000018317	Aak1	55	-19.77
miR-346	ENSRNOG00000047981	Olr728	95	-21.53
miR-346	ENSRNOG00000034089	Ttc21a	64	-18.66
miR-346	ENSRNOG00000006470	Camk1g	97	-22.6
miR-346	ENSRNOG00000001232	Slc19a1	69	-21.86
miR-346	ENSRNOG00000031266	Siae	96	-21.99
miR-346	ENSRNOG00000018877	Zfp629	56	-25.72
miR-346	ENSRNOG00000031801	Ephb3	91	-25.61
miR-346	ENSRNOG00000001622	Impg2	78	-18.85
miR-346	ENSRNOG00000009580	Lca5	74	-17.7
miR-346	ENSRNOG00000030515	Nfasc	58	-22.7
miR-346	ENSRNOG00000014613	Ddah1	90	-22.33
miR-346	ENSRNOG00000032058	Pogk	93	-22.21
miR-346	ENSRNOG00000009183	Szrd1	82	-23.23
miR-346	ENSRNOG00000030888	Rela	61	-46.6
miR-346	ENSRNOG00000018500	Frmd4a	51	-19.13
miR-346	ENSRNOG00000029520	AC110315.1	79	-18.66
miR-346	ENSRNOG00000005620	Lcp2	98	-20.73
miR-346	ENSRNOG00000025155	Lmtk2	85	-42.06
miR-346	ENSRNOG00000009932	Mctp2	90	-38.65
miR-346	ENSRNOG00000033921	Als2cl	65	-49.15
miR-346	ENSRNOG00000029608	Nkain2	58	-21.42
miR-346	ENSRNOG00000029141	Trabd2b	97	-93.66
miR-346	ENSRNOG00000013280	Olfm4	89	-26.08
miR-346	ENSRNOG00000014751	Ret	85	-21.23
miR-346	ENSRNOG00000007866	Clec2dl1	80	-23.7
miR-346	ENSRNOG00000021891	Zdhhc8	62	-23.89
miR-346	ENSRNOG00000012818	Ksr1	65	-74.66
miR-346	ENSRNOG00000021480	Rad9b	81	-18.01
miR-346	ENSRNOG00000031443	Havcr2	77	-22.73
miR-346	ENSRNOG00000029260	Pitpnm2	68	-18.28
miR-346	ENSRNOG00000032215	Cdyl	68	-19.84
miR-346	ENSRNOG00000015121	N4bp1	77	-20.3
miR-346	ENSRNOG00000025527	Mtcl1	75	-14.55
miR-346	ENSRNOG00000014253	Pax2	88	-25.51
miR-346	ENSRNOG00000014060	Mras	69	-15.29
miR-346	ENSRNOG00000010682	Clcn3	76	-27.08
miR-346	ENSRNOG00000020351	Vps4a	91	-22.08
miR-346	ENSRNOG00000024849	Tor1aip2	96	-24.35
miR-346	ENSRNOG00000024322	Shroom2	99	-21.42
miR-346	ENSRNOG00000022911	Hjurp	81	-52.16
miR-346	ENSRNOG00000007984	Amer1	90	-23.02
miR-346	ENSRNOG00000001916	Tmprss15	99	-24.63
miR-346	ENSRNOG00000029535	Nrbp2	86	-48.45
miR-346	ENSRNOG00000011474	Ppp1r3b	84	-24.29
miR-346	ENSRNOG00000024688	Erfe	76	-14.49
miR-346	ENSRNOG00000025581	Vwa2	77	-24.25

miR-346	ENSRNOG00000015902	Cpxm2	91	-18.66
miR-346	ENSRNOG00000028783	Wdr35	58	-25.2
miR-346	ENSRNOG00000033372	Klhl24	95	-20.38
miR-346	ENSRNOG00000025408	Dux4	95	-23.52
miR-346	ENSRNOG00000004921	Nusap1	80	-39.62
miR-346	ENSRNOG00000003562	Susd4	68	-24.91
miR-346	ENSRNOG00000014705	Rbm20	55	-19.74
miR-346	ENSRNOG00000036604	Ifit2	91	-31.38
miR-346	ENSRNOG00000036666	Cybc1	82	-38.74
miR-346	ENSRNOG00000036676	Csnk1d	96	-17
miR-346	ENSRNOG00000036830	Gtsf2	61	-19.59
miR-346	ENSRNOG00000024707	Tp73	54	-19.42
miR-346	ENSRNOG00000024492	Ap1ar	58	-25.12
miR-346	ENSRNOG00000000001	Arsj	93	-18.47
miR-346	ENSRNOG00000002373	Akap1	65	-70.47
miR-346	ENSRNOG00000022778	Pphln1	56	-25.54
miR-346	ENSRNOG00000009819	Vezf1	55	-22.2
miR-346	ENSRNOG00000004320	Tgm4	98	-20.74
miR-346	ENSRNOG00000002470	Ifi47	94	-20.5
miR-346	ENSRNOG00000007400	Srebf2	69	-22.6
miR-346	ENSRNOG00000011796	C1r	98	-22.39
miR-346	ENSRNOG00000020923	Tuft1	55	-17.9
miR-346	ENSRNOG00000037483	Ep400	88	-22.55
miR-346	ENSRNOG00000037505	Ulk1	72	-15.63
miR-346	ENSRNOG00000005130	Ogdh	60	-17.87
miR-346	ENSRNOG00000037795	Ldlrad1	73	-17.25
miR-346	ENSRNOG00000038066	Fam155b	82	-22.99
miR-346	ENSRNOG00000038088	Nanos2	89	-20.59
miR-346	ENSRNOG00000038166	Ptgr2	56	-49.27
miR-346	ENSRNOG00000038328	Gjc2	91	-48.9
miR-346	ENSRNOG00000017146	Nfatc1	76	-49.43
miR-346	ENSRNOG00000006956	Adamtsl1	89	-55.85
miR-346	ENSRNOG00000038766	Atxn1l	87	-18.3
miR-346	ENSRNOG00000005504	Pkp4	95	-20.14
miR-346	ENSRNOG00000015701	Rreb1	80	-25.04
miR-346	ENSRNOG00000049034	Ptrh1	91	-22.25
miR-346	ENSRNOG00000023403	Gtpbp3	68	-13.36
miR-346	ENSRNOG00000019688	Diaph1	89	-25.9
miR-346	ENSRNOG00000010125	Mtrex	95	-27.67
miR-346	ENSRNOG00000019501	Rmnd1	73	-19.82
miR-346	ENSRNOG00000039832	Gpr12	83	-17.73
miR-346	ENSRNOG00000002917	Sec14l5	98	-26.04
miR-346	ENSRNOG00000012007	Slc38a7	65	-23.29
miR-346	ENSRNOG00000002039	Evi5	79	-19.26
miR-346	ENSRNOG00000012004	Ubap1	74	-17.28
miR-346	ENSRNOG00000002979	Tbx19	89	-22.55
miR-346	ENSRNOG00000022239	Trarg1	72	-40.45
miR-346	ENSRNOG00000007942	Fscn3	94	-27.22
miR-346	ENSRNOG00000011912	Tmem38a	81	-20.8
miR-346	ENSRNOG00000004395	Fbxw11	63	-40.87
miR-346	ENSRNOG00000030016	Robo3	61	-26.41
miR-346	ENSRNOG00000008600	Sytl1	96	-25.96
miR-346	ENSRNOG00000025071	Qrich1	54	-14.11
miR-346	ENSRNOG00000009466	Unc45b	90	-19.71
miR-346	ENSRNOG00000004060	Calm1	94	-74.78
miR-346	ENSRNOG00000042501	Tmem250	91	-27.19
miR-346	ENSRNOG00000043390	Samd12	86	-17.06
miR-346	ENSRNOG00000026110	Scml4	52	-17.28
miR-346	ENSRNOG00000000691	Foxn4	55	-49.37
miR-346	ENSRNOG00000007887	Elk4	84	-68.9
miR-346	ENSRNOG00000042749	RGD1562024	58	-45.71

miR-346	ENSRNOG00000036835	Copz1	75	-18.59
miR-346	ENSRNOG00000012918	Mtmr6	89	-17.07
miR-346	ENSRNOG00000005428	Ctbp1	82	-27.38
miR-346	ENSRNOG00000011044	Clmn	84	-20.94
miR-346	ENSRNOG00000003256	Ccng1	80	-19.22
miR-346	ENSRNOG00000042230	RGD1307182	93	-18.02
miR-346	ENSRNOG00000013610	Chrna5	67	-52.78
miR-346	ENSRNOG00000018914	Napg	94	-17.73
miR-346	ENSRNOG00000008922	Trim14	74	-18.14
miR-346	ENSRNOG00000043445	Them5	93	-24.12
miR-346	ENSRNOG00000031136	Ntng1	84	-18.34
miR-346	ENSRNOG00000000123	Rnf19b	82	-24.41
miR-346	ENSRNOG00000020541	Nprl3	86	-23.84
miR-346	ENSRNOG00000043186	Ppil6	98	-27.96
miR-346	ENSRNOG00000013962	Magi2	66	-28.67
miR-346	ENSRNOG00000013589	Cxcl12	50	-38.55
miR-346	ENSRNOG00000025462	Wdr41	89	-25.44
miR-346	ENSRNOG00000069185	Tmem9b	98	-18.39
miR-346	ENSRNOG00000003121	Rtn4rl1	87	-27.4
miR-346	ENSRNOG00000036960	Abcc9	94	-23.35
miR-346	ENSRNOG00000006004	Phc2	71	-26.87
miR-346	ENSRNOG00000043182	Septin6	93	-20.9
miR-346	ENSRNOG00000042189	Rab31	66	-38.1
miR-346	ENSRNOG00000021669	Mybl1	81	-22.43
miR-346	ENSRNOG00000046955	Cbx6	80	-50.98
miR-346	ENSRNOG00000018160	Zswim5	96	-20.32
miR-346	ENSRNOG00000024863	Fam76b	90	-17.78
miR-346	ENSRNOG00000018366	Cracdl	90	-20.36
miR-346	ENSRNOG00000042286	Nsl1	61	-19.15
miR-346	ENSRNOG00000043186	Ppil6	97	-27.96
miR-346	ENSRNOG00000006995	Ano6	50	-45.6
miR-346	ENSRNOG00000045998	Sema6b	88	-25.83
miR-346	ENSRNOG00000007430	Slx4ip	84	-43.74
miR-346	ENSRNOG00000045568	Rbm14	71	-22.29
miR-346	ENSRNOG00000046468	Ptgfr	94	-16.6
miR-346	ENSRNOG00000049378	Dennd5b	57	-23.2
miR-346	ENSRNOG00000048166	Arhgap19	76	-20.41
miR-346	ENSRNOG00000048898	Wasf3	98	-60.24
miR-346	ENSRNOG00000046361	Hoxa7	89	-19.4
miR-346	ENSRNOG00000046458	Pdx1	72	-19.06
miR-346	ENSRNOG00000045952	Ephb4	92	-26.87
miR-346	ENSRNOG00000050223	Rin1	56	-42.18
miR-346	ENSRNOG00000049402	Nbl1	60	-24.12
miR-346	ENSRNOG00000049426	Mmab	86	-23.99
miR-346	ENSRNOG00000046803	Arhgef16	99	-20.14
miR-346	ENSRNOG00000063031	Fam131a	94	-30.59
miR-346	ENSRNOG00000047219	Ube3b	57	-28.91
miR-346	ENSRNOG00000014750	Tasor	73	-25.44
miR-346	ENSRNOG00000047194	Arl13b	83	-16.68
miR-346	ENSRNOG00000050990	Glyatl3	66	-19.01
miR-346	ENSRNOG00000049349	Tafa5	86	-33.61
miR-346	ENSRNOG00000049281	Gba	59	-44.47
miR-346	ENSRNOG00000047035	Fadd	85	-16.92
miR-346	ENSRNOG00000050624	Lysmd4	70	-16.65
miR-346	ENSRNOG00000050564	AABR07073021.1	75	-20.4
miR-346	ENSRNOG00000047734	Chst2	54	-16.65
miR-346	ENSRNOG00000045961	Lyrm7	97	-19.73
miR-346	ENSRNOG00000046002	Micos13	71	-21.45
miR-346	ENSRNOG00000046227	Tprg1l	57	-19.36
miR-346	ENSRNOG00000025643	Ccdc13	71	-44.29
miR-346	ENSRNOG00000050090	Slc6a17	88	-42.26

miR-346	ENSRNOG00000015554	Ankdd1a	93	-23.7
miR-346	ENSRNOG00000010591	Kdm5a	94	-67.75
miR-346	ENSRNOG00000014851	Col4a4	92	-58.11
miR-346	ENSRNOG00000003030	Awat2	67	-15.09
miR-346	ENSRNOG00000028856	Pknox2	53	-44.92
miR-346	ENSRNOG00000010415	Atxn7l1	67	-45.23
miR-346	ENSRNOG00000015921	Esco2	79	-18.66
miR-346	ENSRNOG00000027124	Tdg	88	-19.92
miR-346	ENSRNOG00000061152	Mphosph8	66	-39.7
miR-346	ENSRNOG00000012184	Urgcp	92	-23.23
miR-346	ENSRNOG00000013679	Sema4d	90	-20.21
miR-346	ENSRNOG00000002907	Plekha6	79	-35.32
miR-346	ENSRNOG00000031689	Trim15	95	-49.82
miR-346	ENSRNOG00000014851	Col4a4	92	-30.62
miR-346	ENSRNOG00000024533	Eogt	96	-24.68
miR-346	ENSRNOG00000005641	Fbxl4	62	-25.05
miR-346	ENSRNOG00000018914	Napg	96	-17.73
miR-346	ENSRNOG00000022500	Rwdd4	54	-20.92
miR-346	ENSRNOG00000013602	Usp48	59	-45.75
miR-346	ENSRNOG00000017137	Eef1akmt2	79	-59.15
miR-346	ENSRNOG00000014060	Mras	82	-15.29
miR-346	ENSRNOG00000052814	Ankrd27	52	-23.04
miR-346	ENSRNOG00000009484	Ptcd3	90	-21.79
miR-346	ENSRNOG00000057040	Spns2	72	-18.44
miR-346	ENSRNOG00000002912	Parn	67	-20.19
miR-346	ENSRNOG00000022354	Pusl1	93	-21.99
miR-346	ENSRNOG00000016885	Klf6	87	-21.17
miR-346	ENSRNOG00000029466	RGD1562608	95	-19.92
miR-346	ENSRNOG00000002506	Cdkl2	63	-30.53
miR-346	ENSRNOG00000052022	Pnma3	85	-19.76
miR-346	ENSRNOG00000020769	Crebrf	70	-17.91
miR-346	ENSRNOG00000061876	Aldh4a1	67	-19.39
miR-346	ENSRNOG00000053735	Hebp2	93	-21.75
miR-346	ENSRNOG00000059344	Tpcn1	54	-32.66
miR-346	ENSRNOG00000001658	Kcnj6	80	-61.99
miR-346	ENSRNOG00000032311	Ttll8	90	-26.45
miR-346	ENSRNOG00000055527	Arap3	95	-30.52
miR-346	ENSRNOG00000018416	Ttbk1	75	-34.35
miR-346	ENSRNOG00000052486	Kcna6	78	-48.81
miR-346	ENSRNOG00000000809	Atat1	66	-21.55
miR-346	ENSRNOG00000007014	Cnksr2	87	-22.57
miR-346	ENSRNOG00000055340	Ski	64	-44.27
miR-346	ENSRNOG00000046227	Tprg1l	51	-19.36
miR-346	ENSRNOG00000056889	Vps33a	78	-21.79
miR-346	ENSRNOG00000000467	Ring1	74	-29.6
miR-346	ENSRNOG00000051993	Gdf10	54	-17.22
miR-346	ENSRNOG00000000258	Prmt7	97	-19.35
miR-346	ENSRNOG00000018327	Fank1	59	-16.74
miR-346	ENSRNOG00000005955	Naalad2	98	-17.73
miR-346	ENSRNOG00000053753	AABR07070307.1	77	-23.04
miR-346	ENSRNOG00000054203	Sim2	76	-23.75
miR-346	ENSRNOG00000059897	Gjb5	96	-23.7
miR-346	ENSRNOG00000006338	Lrp6	78	-17.07
miR-346	ENSRNOG00000036842	Smug1	74	-25.73
miR-346	ENSRNOG00000000924	Slc7a1	59	-24.43
miR-346	ENSRNOG00000052424	RGD1306556	57	-51.35
miR-346	ENSRNOG00000015986	Rassf8	99	-27.32
miR-346	ENSRNOG00000056907	Nipbl	96	-25.34
miR-346	ENSRNOG00000058329	Prrx2	79	-17.23
miR-346	ENSRNOG00000057367	Glud1	78	-20.36
miR-346	ENSRNOG00000054495	Cldn2	95	-19.48

miR-346	ENSRNOG00000010682	Clcn3	54	-27.08
miR-346	ENSRNOG00000003310	Tmem63a	91	-42.74
miR-346	ENSRNOG00000013004	Akr1d1	56	-22.79
miR-346	ENSRNOG00000010803	Gabra5	83	-20.79
miR-346	ENSRNOG00000057542	Zfp488	76	-20.64
miR-346	ENSRNOG00000013178	Cmip	73	-24.21
miR-346	ENSRNOG00000028255	Usp36	61	-19.23
miR-346	ENSRNOG00000011202	Chrna4	52	-25.65
miR-346	ENSRNOG00000020216	Gmpr2	90	-15.1
miR-346	ENSRNOG00000052391	Mettl6	73	-21.54
miR-346	ENSRNOG00000017791	Arhgap12	89	-16.27
miR-346	ENSRNOG00000017212	Spsb1	80	-48.84
miR-346	ENSRNOG00000015417	Kansl3	65	-40.23
miR-346	ENSRNOG00000013089	Kif13b	52	-181.42
miR-346	ENSRNOG00000051619	Asb2	60	-13.36
miR-346	ENSRNOG00000060329	Emb	92	-25.97
miR-346	ENSRNOG00000055925	Zc3h7b	69	-29.44
miR-346	ENSRNOG00000052140	Ogg1	90	-17.74
miR-346	ENSRNOG00000058288	Tcf19	81	-16.73
miR-346	ENSRNOG00000015553	Gatad2b	63	-16.13
miR-346	ENSRNOG00000048297	Qk	91	-25.94
miR-346	ENSRNOG00000061348	Fam53b	61	-59.15
miR-346	ENSRNOG00000028075	Dnaaf8	89	-53.85
miR-346	ENSRNOG00000057470	Pla2g12a	80	-21.17
miR-346	ENSRNOG00000057756	Osbpl2	96	-17.82
miR-346	ENSRNOG00000000785	Trim39	85	-69.19
miR-346	ENSRNOG00000054964	Aoah	86	-23.1
miR-346	ENSRNOG00000054669	RGD1305110	66	-24.8
miR-346	ENSRNOG00000013048	Pde7a	97	-16.48
miR-346	ENSRNOG00000058739	Snn	66	-51.57
miR-346	ENSRNOG00000060530	Cdc34	86	-24.51
miR-346	ENSRNOG00000014076	Mbnl1	99	-21.95
miR-346	ENSRNOG00000016258	Zfp516	91	-87.52
miR-346	ENSRNOG00000010217	Prrc2b	68	-43.81
miR-346	ENSRNOG00000005633	Zbtb49	68	-22.43
miR-346	ENSRNOG00000054963	Rhbdd1	50	-50.44
miR-346	ENSRNOG00000055769	AABR07054286.1	79	-21.95
miR-346	ENSRNOG00000009661	Tgds	60	-22.72
miR-346	ENSRNOG00000059463	Slc39a1	80	-22.96
miR-346	ENSRNOG00000053680	Kansl1	96	-33.45
miR-346	ENSRNOG00000022288	Pafah2	70	-19.29
miR-346	ENSRNOG00000017220	Tcirg1	95	-27.25
miR-346	ENSRNOG00000031494	Armh1	90	-27.35
miR-346	ENSRNOG00000005052	Slc39a9	93	-69.58
miR-346	ENSRNOG00000059589	Lmbr1	85	-36.13
miR-346	ENSRNOG00000042860	Pappa2	82	-15.93
miR-346	ENSRNOG00000004670	Ddx56	78	-17.32
miR-346	ENSRNOG00000053337	Ly49s6	61	-23.42
miR-346	ENSRNOG00000052113	Ppp1r9b	89	-46.62
miR-346	ENSRNOG00000030466	Cyp2j16	99	-17.92
miR-346	ENSRNOG00000054033	Adap1	85	-15.97
miR-346	ENSRNOG00000056162	Man2b2	83	-13.36
miR-346	ENSRNOG00000006995	Ano6	59	-45.6
miR-346	ENSRNOG00000056362	Ttll13	88	-50.86
miR-346	ENSRNOG00000009580	Lca5	92	-19.9
miR-346	ENSRNOG00000059683	Mpp2	70	-22.19
miR-346	ENSRNOG00000049498	Nat1	71	-27.29
miR-346	ENSRNOG00000057814	Nsdhl	83	-25.66
miR-346	ENSRNOG00000059615	Exoc6b	96	-24.27
miR-346	ENSRNOG00000002182	Septin11	55	-19.61
miR-346	ENSRNOG00000056793	Hsf5	92	-22.17

miR-346	ENSRNOG00000043498	Sik2	83	-67.85
miR-346	ENSRNOG00000000826	Flot1	65	-44.66
miR-346	ENSRNOG00000057231	Ddx3	98	-18.61
miR-346	ENSRNOG00000013588	Glra1	65	-24.27
miR-346	ENSRNOG00000059328	Olr1657	98	-20.54
miR-346	ENSRNOG00000062002	Kcna3	89	-22.37
miR-346	ENSRNOG00000002980	Tsr1	70	-19.58
miR-346	ENSRNOG00000000463	Col11a2	89	-21.94
miR-346	ENSRNOG00000011841	Map2	57	-42.44
miR-346	ENSRNOG00000001031	Ocm	89	-16.81
miR-346	ENSRNOG00000002764	Mettl16	81	-16.13
miR-346	ENSRNOG00000011653	Washc2c	65	-23.23
miR-346	ENSRNOG00000025460	Tmem71	94	-24.73
miR-346	ENSRNOG00000007393	Ndrg1	78	-37.85
miR-346	ENSRNOG00000013844	Fam172a	78	-29.97
miR-346	ENSRNOG00000063495	Olr6	98	-16.78
miR-346	ENSRNOG00000065011	St6galnac2	73	-51.16
miR-346	ENSRNOG00000068826	Mgat1	65	-80.77
miR-346	ENSRNOG00000064880	Sgsh	54	-44.01
miR-346	ENSRNOG00000027360	Srsf7	75	-18.49
miR-346	ENSRNOG00000005310	Cul1	63	-50.86
miR-346	ENSRNOG00000066614	RGD1561143	85	-77.64
miR-346	ENSRNOG00000013962	Magi2	64	-28.67
miR-346	ENSRNOG00000002951	Ccnb3	94	-25.57
miR-346	ENSRNOG00000031852	Lsamp	87	-18.74
miR-346	ENSRNOG00000066008	Pecam1	90	-25.3
miR-346	ENSRNOG00000070875	Nat8f4	74	-21.14
miR-346	ENSRNOG00000070828	Fbxl14	93	-55.82
miR-346	ENSRNOG00000065606	Gid8	56	-64.23
miR-346	ENSRNOG00000013752	Elavl3	72	-20.02
miR-346	ENSRNOG00000012818	Ksr1	97	-98.81
miR-346	ENSRNOG00000018877	Zfp629	68	-16.79
miR-346	ENSRNOG00000060580	Slc10a3	50	-61.11
miR-346	ENSRNOG00000019807	Sufu	57	-53.41
miR-346	ENSRNOG00000004672	Sec14l2	89	-41.58
miR-346	ENSRNOG00000070837	Olr1344	53	-17.3
miR-346	ENSRNOG00000028701	C8g	71	-78.18
miR-346	ENSRNOG00000069187	Inip	87	-16.01
miR-346	ENSRNOG00000067239	Ppm1l	53	-90.79
miR-346	ENSRNOG00000011475	Srcin1	64	-25.52
miR-346	ENSRNOG00000015450	Ppp1r16a	90	-87.37
miR-346	ENSRNOG00000006264	Itfg2	70	-23.19
miR-346	ENSRNOG00000068415	Lrrc15	81	-38.71
miR-346	ENSRNOG00000071062	Ndufa5	98	-21.52
miR-346	ENSRNOG00000053370	Adnp2	74	-30.83
miR-346	ENSRNOG00000011491	Dnajc13	82	-20.03
miR-346	ENSRNOG00000004904	Pa2g4	73	-25.15
miR-346	ENSRNOG00000064497	Olr1585	88	-35.1
miR-346	ENSRNOG00000021478	Tpd52l1	84	-28.07
miR-346	ENSRNOG00000070251	Vom1r23	89	-21.71
miR-346	ENSRNOG00000064692	Kif4b	95	-16.95
miR-346	ENSRNOG00000012582	Eif4ebp1	85	-24.89
miR-346	ENSRNOG00000002001	Itsn1	53	-77.8
miR-346	ENSRNOG00000066815	dsr-2	93	-18.72
miR-346	ENSRNOG00000017707	Abi2	73	-29.74
miR-346	ENSRNOG00000067424	Olr1077	82	-13.86
miR-346	ENSRNOG00000070188	Olr1536	99	-48.5
miR-346	ENSRNOG00000030888	Rela	62	-46.6
miR-346	ENSRNOG00000002823	Mapk9	84	-20.88
miR-346	ENSRNOG00000005872	Tcf7	60	-44.11
miR-346	ENSRNOG00000049511	Safb2	78	-75.33

miR-346	ENSRNOG00000016459	Eif3j	98	-20.1
miR-346	ENSRNOG00000001190	Git2	91	-17.73
miR-346	ENSRNOG00000064516	Rtl6	73	-100.64
miR-346	ENSRNOG00000008872	Tmem68	99	-18.1
miR-346	ENSRNOG00000001515	Map3k20	75	-40.1
miR-346	ENSRNOG00000005825	Lyz2	74	-21.08
miR-346	ENSRNOG00000016267	Chst15	54	-135.23
miR-346	ENSRNOG00000064442	Tmem74b	79	-139.44
miR-346	ENSRNOG00000014076	Mbnl1	75	-40.31
miR-346	ENSRNOG00000056907	Nipbl	93	-46.59
miR-346	ENSRNOG00000062627	Olr1435	75	-26.07
miR-346	ENSRNOG00000064037	Hpse2	85	-47.88
miR-346	ENSRNOG00000002979	Tbx19	90	-22.55
miR-346	ENSRNOG00000027282	Cep68	86	-21.02
miR-346	ENSRNOG00000065325	Olr1214	93	-21.64
miR-346	ENSRNOG00000019295	Rab12	97	-23.61
miR-346	ENSRNOG00000052730	Psmb3	80	-67.97
miR-346	ENSRNOG00000049498	Nat1	70	-27.29
miR-346	ENSRNOG00000032472	Adgrg2	94	-15.55
miR-346	ENSRNOG00000067973	Olr857	95	-23.57
miR-346	ENSRNOG00000006958	AC126572.1	83	-24.8
miR-346	ENSRNOG00000031495	Tmem170b	98	-23.36
miR-346	ENSRNOG00000000164	Lamp2	86	-20.4
miR-346	ENSRNOG00000000985	Cpsf4	97	-37.96
miR-346	ENSRNOG00000048297	Qk	89	-25.94
miR-346	ENSRNOG00000002989	Nmt1	88	-19.95
miR-346	ENSRNOG00000069633	Itpripl2	74	-22.88
miR-346	ENSRNOG00000016578	Mtpap	86	-27.79
miR-346	ENSRNOG00000022870	Smad5	86	-33.07
miR-346	ENSRNOG00000066151	Fem1a	80	-90.65
miR-346	ENSRNOG00000046330	AABR07029581.1	88	-74.74
miR-346	ENSRNOG00000059480	Supt20h	50	-19.6
miR-346	ENSRNOG00000003248	Mpzl1	65	-41.52
miR-346	ENSRNOG00000027012	Usp54	53	-53.06
miR-346	ENSRNOG00000000488	Hmga1	58	-22.26
miR-346	ENSRNOG00000027873	RT1-M1-4	81	-23.65
miR-346	ENSRNOG00000049511	Safb2	65	-21.39
miR-346	ENSRNOG00000003925	Hhat	97	-18.81
miR-346	ENSRNOG00000002267	Chic2	86	-17.47
miR-346	ENSRNOG00000005818	Elapor2	98	-18.56
miR-346	ENSRNOG00000068717	Ccdc167	89	-18.66
miR-346	ENSRNOG00000070382	Hyal3	93	-22.35
miR-346	ENSRNOG00000019953	Ube2f	72	-21.95
miR-346	ENSRNOG00000005606	Med1	64	-20.42
miR-346	ENSRNOG00000020624	Acadsb	93	-24.97
miR-346	ENSRNOG00000006718	Rbm33	76	-20.28
miR-346	ENSRNOG00000024729	Pax5	92	-17.73
miR-346	ENSRNOG00000018159	Anxa4	81	-21.87
miR-346	ENSRNOG00000070811	Mtif3	91	-53.29
miR-346	ENSRNOG00000007242	Ehmt1	50	-49.57
miR-346	ENSRNOG00000067464	Cysltr2	73	-24.06
miR-346	ENSRNOG00000003590	Tom1l2	65	-29.06
miR-346	ENSRNOG00000017441	Tpm3	55	-29.55
miR-346	ENSRNOG00000013920	Arhgap26	59	-26.52
miR-346	ENSRNOG00000064480	Gmfb	67	-32.46
miR-346	ENSRNOG00000036666	Cybc1	82	-21.11
miR-346	ENSRNOG00000005888	Fsip1	89	-41.87
miR-346	ENSRNOG00000060979	Hspa13	84	-23.76
miR-346	ENSRNOG00000059589	Lmbr1	85	-55.76
miR-346	ENSRNOG00000065883	Mmgt1	82	-23.9
miR-346	ENSRNOG00000014294	Ptpn6	88	-30.91

miR-346	ENSRNOG00000042340	Thoc2l	54	-29.59
miR-346	ENSRNOG00000004132	Lasp1	60	-49.82
miR-346	ENSRNOG00000011460	Arfgef3	84	-47.14
miR-346	ENSRNOG00000008752	Nrf1	98	-51.55
miR-346	ENSRNOG00000011320	Igfbpl1	76	-18.79
miR-346	ENSRNOG00000062659	RGD1565987	51	-26.61
miR-346	ENSRNOG00000062809	RT1-M10-1	70	-122.01
miR-346	ENSRNOG00000004269	Myt1l	75	-20.8
miR-346	ENSRNOG00000009421	Ivd	66	-15.95
miR-346	ENSRNOG00000013636	Ppil3	56	-23.36
miR-346	ENSRNOG00000028630	Ksr2	68	-98.04
miR-346	ENSRNOG00000048617	Ensa	96	-19.28
miR-346	ENSRNOG00000032878	Cxxc5	68	-44.44
miR-346	ENSRNOG00000068024	B3gnt8	57	-68.22
miR-346	ENSRNOG00000070268	Pnrc2	92	-20.52
miR-346	ENSRNOG00000052486	Kcna6	78	-42.09
miR-346	ENSRNOG00000066275	Cyb5d1	82	-26.91
miR-346	ENSRNOG00000019960	Ino80e	97	-44.25
miR-346	ENSRNOG00000063640	Bri3bp	66	-49.22
miR-346	ENSRNOG00000018987	Crebzf	50	-17.26
miR-346	ENSRNOG00000021156	Vegfb	90	-25.41
miR-346	ENSRNOG00000010911	Tmem108	56	-48.49
miR-346	ENSRNOG00000004827	Papola	87	-23.01
miR-346	ENSRNOG00000004100	Trib1	86	-23.83
miR-346	ENSRNOG00000011639	Mrpl47	99	-58.34
miR-346	ENSRNOG00000046922	Phactr4	85	-22.21
miR-346	ENSRNOG00000007671	Smu1	97	-18.58
miR-346	ENSRNOG00000065544	Olr1341	98	-60.66
miR-346	ENSRNOG00000004921	Nusap1	84	-39.62
miR-346	ENSRNOG00000066252	Vps13b	64	-19.58
miR-346	ENSRNOG00000043035	Fbxl13	93	-17.06
miR-346	ENSRNOG00000063347	Nrip2	70	-19.06
miR-346	ENSRNOG00000010753	Aig1	97	-16.56
miR-346	ENSRNOG00000067468	RGD1562811	90	-19.72
miR-346	ENSRNOG00000025715	Dynlrb1	92	-67.26
miR-346	ENSRNOG00000070837	Olr1344	55	-17.3
miR-346	ENSRNOG00000063590	Fzd4	75	-22.75
miR-346	ENSRNOG00000000763	RT1-M3-1	96	-41.59
miR-346	ENSRNOG00000036676	Csnk1d	54	-40.98
miR-346	ENSRNOG00000066176	Prrg3	67	-48.7
miR-346	ENSRNOG00000058572	Nek9	85	-24.74
miR-346	ENSRNOG00000024818	Eva1b	57	-65.14
miR-346	ENSRNOG00000063927	Zfp113	85	-29.62
miR-346	ENSRNOG00000038258	Upb1	92	-108.98
miR-346	ENSRNOG00000068940	Vom1r7	87	-69.79
miR-346	ENSRNOG00000052704	Tctn2	52	-24.58
miR-346	ENSRNOG00000047235	Vps26b	55	-46.09
miR-346	ENSRNOG00000021174	Macrod1	86	-101.87
miR-346	ENSRNOG00000018027	Taf5l	73	-18
miR-346	ENSRNOG00000016978	AI467606	77	-23.49
miR-346	ENSRNOG00000018457	Ptpa	52	-165.37
miR-346	ENSRNOG00000063443	Pirt	93	-45.46
miR-346	ENSRNOG00000008922	Trim14	77	-18.14
miR-346	ENSRNOG00000053881	Baz2a	75	-36.64
miR-346	ENSRNOG00000043143	Eml1	96	-21.05
miR-346	ENSRNOG00000009421	Ivd	64	-43.19
miR-346	ENSRNOG00000021478	Tpd52l1	83	-28.07
miR-346	ENSRNOG00000003546	Tnfrsf12a	84	-54.35
miR-346	ENSRNOG00000022249	Mllt10	87	-14.59
miR-346	ENSRNOG00000002764	Mettl16	74	-18.67
miR-346	ENSRNOG00000004629	Fkbp3	92	-26.08

miR-346	ENSRNOG00000049498	Nat1	76	-27.29
miR-346	ENSRNOG00000008173	Sesn3	82	-35.41
miR-346	ENSRNOG00000011498	Psip1	81	-18.29
miR-346	ENSRNOG00000062002	Kcna3	89	-41.58
miR-346	ENSRNOG00000008775	Lrguk	91	-40.64
miR-346	ENSRNOG00000049511	Safb2	65	-126.84
miR-346	ENSRNOG00000016780	RGD1310951	61	-28.05
miR-346	ENSRNOG00000018877	Zfp629	61	-16.79
miR-346	ENSRNOG00000052704	Tctn2	63	-69.62
miR-346	ENSRNOG00000059615	Exoc6b	97	-24.27
miR-346	ENSRNOG00000012719	Tdrd12	83	-46.8
miR-346	ENSRNOG00000063099	Gcnt7	77	-90.07
miR-346	ENSRNOG00000020623	Aldh16a1	77	-49.19
miR-346	ENSRNOG00000015173	Mbtps1	68	-56.27
miR-346	ENSRNOG00000025071	Qrich1	60	-14.11
miR-346	ENSRNOG00000018413	Per3	86	-18.49
miR-346	ENSRNOG00000062406	Mosmo	71	-46.45
miR-346	ENSRNOG00000053060	Zfc3h1	99	-22.23
miR-346	ENSRNOG00000007738	Hm13	52	-27.95
miR-346	ENSRNOG00000042189	Rab31	68	-38.1
miR-346	ENSRNOG00000067363	Xkrx	93	-19.74
miR-346	ENSRNOG00000021242	Adam33	82	-27.74
miR-346	ENSRNOG00000065168	RGD1566007	72	-112.89
miR-346	ENSRNOG00000000258	Prmt7	96	-19.35
miR-346	ENSRNOG00000005393	Sertad2	51	-50.68
miR-346	ENSRNOG00000020179	Nt5c2	82	-47.23
miR-346	ENSRNOG00000015409	Usp5	66	-25.03
miR-346	ENSRNOG00000028404	Ppp1r1b	72	-17.05
miR-346	ENSRNOG00000020880	Klhl18	50	-24.74
miR-346	ENSRNOG00000022153	Tmem134	62	-22.51
miR-346	ENSRNOG00000017949	Mvb12a	97	-18.88
miR-346	ENSRNOG00000001602	Ltn1	86	-19.23
miR-346	ENSRNOG00000007100	Ccdc136	72	-14.96
miR-346	ENSRNOG00000021881	Metap2	76	-19.88
miR-346	ENSRNOG00000017545	Mrs2	94	-23.25
miR-346	ENSRNOG00000016470	Ndufa10l1	61	-29.84
miR-346	ENSRNOG00000066008	Pecam1	90	-42.19
miR-346	ENSRNOG00000012818	Ksr1	75	-24.15
miR-346	ENSRNOG00000018627	Plekhb1	93	-30.97
miR-346	ENSRNOG00000009995	Rapgef6	97	-46.97
miR-346	ENSRNOG00000011007	Ube2o	94	-19.5
miR-346	ENSRNOG00000018322	Picalm	58	-105.65
miR-346	ENSRNOG00000065563	Rufy2	64	-75.92
miR-346	ENSRNOG00000005504	Pkp4	96	-20.14
miR-346	ENSRNOG00000063051	Cacng6	94	-20.96
miR-346	ENSRNOG00000014076	Mbnl1	76	-40.31
miR-346	ENSRNOG00000070230	Nhlrc3	77	-24.06
miR-346	ENSRNOG00000022781	Ccser2	80	-19.09
miR-346	ENSRNOG00000055579	Tmbim6	87	-25.92
miR-346	ENSRNOG00000007992	Srsf10	96	-20.32
miR-346	ENSRNOG00000015813	Ubr2	82	-36.36
miR-346	ENSRNOG00000010898	Prok2	83	-34.45
miR-346	ENSRNOG00000019438	Rnf31	94	-58.98
miR-346	ENSRNOG00000059005	Kcnj1	68	-24.28
miR-346	ENSRNOG00000013912	Slc30a7	53	-44.49
miR-346	ENSRNOG00000020624	Acadsb	72	-24.97
miR-346	ENSRNOG00000005963	Rab2a	92	-21.73
miR-346	ENSRNOG00000010109	Nol9	66	-20.26
miR-346	ENSRNOG00000021013	Stx3	72	-20.11
miR-346	ENSRNOG00000008764	Otud5	64	-26.91
miR-346	ENSRNOG00000010959	Klhl25	76	-17.2

miR-346	ENSRNOG00000018655	Adsl	52	-66.44
miR-346	ENSRNOG00000018166	Prkab2	66	-19.24
miR-346	ENSRNOG00000067250	Olfr917	99	-28.66
miR-346	ENSRNOG00000065648	Olr1687	58	-29.98
miR-346	ENSRNOG00000008297	Oser1	67	-21.5
miR-346	ENSRNOG00000065646	Olr7	64	-39.53
miR-346	ENSRNOG00000067718	Olr289	97	-78.33
miR-346	ENSRNOG00000066108	Vom1r11	97	-23.29
miR-346	ENSRNOG00000008524	Glcci1	95	-21.48
miR-346	ENSRNOG00000054446	Sltm	89	-33.06
miR-346	ENSRNOG00000007922	Cldn19	61	-26.18
miR-346	ENSRNOG00000037897	Rps19	96	-24.34
miR-346	ENSRNOG00000000488	Hmga1	54	-22.26
miR-346	ENSRNOG00000063676	Lhfpl4	55	-25.02
miR-346	ENSRNOG00000019532	Emc10	57	-56.15
miR-346	ENSRNOG00000032183	Scmh1	97	-48.9
miR-346	ENSRNOG00000018916	Bcl7c	65	-21.41
miR-346	ENSRNOG00000001429	Lrwd1	92	-19.54
miR-346	ENSRNOG00000016309	Rgp1	59	-76.56
miR-346	ENSRNOG00000019873	B3gat3	64	-96.19
miR-346	ENSRNOG00000054515	Fgd6	56	-21.46
miR-346	ENSRNOG00000064037	Hpse2	82	-47.88
miR-346	ENSRNOG00000002196	Ociad2	77	-18.92
miR-346	ENSRNOG00000002014	Stap1	89	-19.21
miR-346	ENSRNOG00000014475	Slc31a1	69	-21.21
miR-346	ENSRNOG00000042679	Lcor	62	-20.97
miR-346	ENSRNOG00000066159	Cpm	62	-77.82
miR-346	ENSRNOG00000064987	Vom1r16	86	-39.85
miR-346	ENSRNOG00000068138	Aldh3b2	61	-34.17
miR-346	ENSRNOG00000008053	Atp8a2	74	-25.51
miR-346	ENSRNOG00000014027	RGD1304728	50	-21.93
miR-346	ENSRNOG00000027954	Cdh15	77	-25.55
miR-346	ENSRNOG00000020975	Mrpl49	97	-48.66
miR-346	ENSRNOG00000001117	Fbxl18	51	-26.61
miR-346	ENSRNOG00000065168	RGD1566007	62	-129.93
miR-346	ENSRNOG00000012061	Prkcb	91	-21.63
miR-346	ENSRNOG00000049497	Eif6	54	-18.62
miR-346	ENSRNOG00000002793	Sstr2	97	-43.16
miR-346	ENSRNOG00000067971	Sprn	56	-67.17
miR-346	ENSRNOG00000071001	Trim56	61	-95.19
miR-346	ENSRNOG00000058681	Tsga10	96	-22.63
miR-346	ENSRNOG00000065524	Vom1r44	91	-39.17
miR-346	ENSRNOG00000014071	Ddb2	87	-25.66
miR-346	ENSRNOG00000051965	Smad4	67	-18.82
miR-346	ENSRNOG00000018381	Cdkal1	88	-43.92
miR-346	ENSRNOG00000069710	Nat8f2	74	-21.14
miR-346	ENSRNOG00000011595	Senp8	69	-26.38
miR-346	ENSRNOG00000005279	Ndufaf7	94	-36.66
miR-346	ENSRNOG00000020457	Tacc2	82	-44.54
miR-346	ENSRNOG00000020713	Osbpl5	52	-19.58
miR-346	ENSRNOG00000019501	Rmnd1	76	-19.82
miR-346	ENSRNOG00000009019	Slc6a6	76	-61.74
miR-346	ENSRNOG00000067096	Capza3	93	-24.67
miR-346	ENSRNOG00000015474	Nelfa	59	-28.51
miR-346	ENSRNOG00000006932	AABR07029272.1	72	-18.14
miR-346	ENSRNOG00000018884	Ttc13	79	-39.23
miR-346	ENSRNOG00000038166	Ptgr2	57	-49.27
miR-346	ENSRNOG00000019533	Klhl3	59	-17.36
miR-346	ENSRNOG00000005872	Tcf7	67	-22.12
miR-346	ENSRNOG00000026647	Cxcl16	91	-24.97
miR-346	ENSRNOG00000010588	Tns2	66	-51.75

miR-346	ENSRNOG00000005362	Rab3ip	92	-21.07
miR-346	ENSRNOG00000001712	Alg3	88	-100.34
miR-346	ENSRNOG00000005428	Ctbp1	82	-49.52
miR-346	ENSRNOG00000031801	Ephb3	92	-25.61
miR-346	ENSRNOG00000008034	Tmeff1	89	-25.04
miR-346	ENSRNOG00000028701	C8g	92	-30.81
miR-346	ENSRNOG00000021660	Nprl2	54	-49.99
miR-346	ENSRNOG00000004298	Dpys	84	-17.9

Table S6 GO enrichment analysis of the target genes of miR-193b-3p

ID	Term	ListHits	PopHits	*p* value
GO:0042982	amyloid precursor protein metabolic process	5	10	0.00003
GO:0030335	positive regulation of cell migration	26	258	0.00003
GO:0030425	dendrite	44	571	0.00006
GO:0031434	mitogen-activated protein kinase kinase binding	6	19	0.00009
GO:0005737	cytoplasm	256	5042	0.00009
GO:0035307	positive regulation of protein dephosphorylation	7	28	0.00011
GO:0006930	substrate-dependent cell migration, cell extension	4	8	0.00018
GO:0031435	mitogen-activated protein kinase kinase kinase binding	6	25	0.00045
GO:0005905	clathrin-coated pit	9	56	0.00046
GO:0009925	basal plasma membrane	9	58	0.0006
GO:1904925	positive regulation of autophagy of mitochondrion in response to mitochondrial depolarization	3	5	0.00067
GO:0030199	collagen fibril organization	8	51	0.00111
GO:0030948	negative regulation of vascular endothelial growth factor receptor signaling pathway	4	12	0.00112
GO:0043149	stress fiber assembly	5	20	0.00112
GO:0030424	axon	34	470	0.00126
GO:0002573	myeloid leukocyte differentiation	3	6	0.0013
GO:0015174	basic amino acid transmembrane transporter activity	3	6	0.0013
GO:0019901	protein kinase binding	37	528	0.00139
GO:0000209	protein polyubiquitination	15	153	0.00179
GO:0005794	Golgi apparatus	62	1030	0.0021
GO:0032400	melanosome localization	3	7	0.0022
GO:0048729	tissue morphogenesis	3	7	0.0022
GO:0016055	Wnt signaling pathway	13	126	0.00224
GO:0006468	protein phosphorylation	34	489	0.00241
GO:0032266	phosphatidylinositol-3-phosphate binding	7	46	0.0027
GO:0045732	positive regulation of protein catabolic process	9	72	0.00287
GO:0047485	protein N-terminus binding	13	130	0.00295
GO:0035556	intracellular signal transduction	28	385	0.00302
GO:0035097	histone methyltransferase complex	5	25	0.00324
GO:0015824	proline transport	3	8	0.00341
GO:0032651	regulation of interleukin-1 beta production	3	8	0.00341
GO:0035524	proline transmembrane transport	3	8	0.00341
GO:0038036	sphingosine-1-phosphate receptor activity	3	8	0.00341
GO:0050920	regulation of chemotaxis	3	8	0.00341
GO:1903053	regulation of extracellular matrix organization	3	8	0.00341
GO:0035615	clathrin adaptor activity	4	16	0.0036
GO:0051571	positive regulation of histone H3-K4 methylation	4	16	0.0036
GO:0071805	potassium ion transmembrane transport	13	134	0.00384
GO:0005654	nucleoplasm	149	2926	0.00387
GO:0019888	protein phosphatase regulator activity	6	37	0.00388
GO:0000159	protein phosphatase type 2A complex	4	17	0.00455
GO:0036010	protein localization to endosome	3	9	0.00496
GO:0036289	peptidyl-serine autophosphorylation	3	9	0.00496
GO:0042754	negative regulation of circadian rhythm	3	9	0.00496
GO:0051400	BH domain binding	3	9	0.00496
GO:2000617	positive regulation of histone H3-K9 acetylation	3	9	0.00496
GO:0005634	nucleus	230	4782	0.00516
GO:0061564	axon development	4	18	0.00566
GO:0006631	fatty acid metabolic process	9	80	0.00583
GO:0005829	cytosol	173	3497	0.00587
GO:0042733	embryonic digit morphogenesis	8	67	0.00638
GO:0000122	negative regulation of transcription by RNA polymerase	52	878	0.00639
GO:0043507	positive regulation of JUN kinase activity	6	41	0.00653
GO:0005769	early endosome	20	262	0.00667
GO:0030838	positive regulation of actin filament polymerization	7	54	0.00671
GO:0015816	glycine transport	3	10	0.00687
GO:0044154	histone H3-K14 acetylation	3	10	0.00687
GO:0070776	MOZ/MORF histone acetyltransferase complex	3	10	0.00687
GO:0072542	protein phosphatase activator activity	3	10	0.00687
GO:1902414	protein localization to cell junction	3	10	0.00687
GO:2001243	negative regulation of intrinsic apoptotic signaling	5	30	0.00732
GO:0005547	phosphatidylinositol-3,4,5-trisphosphate binding	6	43	0.00826
GO:0007032	endosome organization	6	43	0.00826
GO:0032147	activation of protein kinase activity	8	70	0.00829
GO:0042060	wound healing	13	147	0.0083
GO:0090141	positive regulation of mitochondrial fission	4	20	0.00839

GO:0005790	smooth endoplasmic reticulum	5	31	0.00844
GO:0030131	clathrin adaptor complex	3	11	0.00916
GO:0035308	negative regulation of protein dephosphorylation	3	11	0.00916
GO:0072546	EMC complex	3	11	0.00916
GO:0097237	cellular response to toxic substance	3	11	0.00916
GO:0043161	proteasome-mediated ubiquitin-dependent protein catabolic process	15	184	0.00993

Table S7 KEGG enrichment analysis of the target genes of miR-193b-3p

ID	Term	ListHits	PopHits	*p* value
rno04010	MAPK signaling pathway	23	293	0.00058
rno04340	Hedgehog signaling pathway	8	52	0.00061
rno04012	ErbB signaling pathway	10	85	0.00117
rno04072	Phospholipase D signaling pathway	14	150	0.00138
rno05202	Transcriptional misregulation in cancer	16	193	0.00225
rno04390	Hippo signaling pathway	14	158	0.00226
rno05206	MicroRNAs in cancer	14	163	0.00301
rno04392	Hippo signaling pathway - multiple species	5	28	0.00337
rno04710	Circadian rhythm	5	30	0.00460
rno04912	GnRH signaling pathway	9	90	0.00615
rno04014	Ras signaling pathway	16	231	0.01245
rno04144	Endocytosis	18	275	0.01431
rno00563	Glycosylphosphatidylinositol (GPI)-anchor biosynthesis	4	26	0.01478
rno05211	Renal cell carcinoma	7	70	0.01499
rno04062	Chemokine signaling pathway	13	182	0.01833
rno04666	Fc gamma R-mediated phagocytosis	8	95	0.02496
rno04713	Circadian entrainment	8	98	0.02939
rno04114	Oocyte meiosis	9	118	0.03174
rno04020	Calcium signaling pathway	15	238	0.03237
rno04151	PI3K-Akt signaling pathway	20	345	0.03289
rno04380	Osteoclast differentiation	9	121	0.03645
rno05221	Acute myeloid leukemia	6	68	0.04040
rno05166	Human T-cell leukemia virus 1 infection	15	246	0.04144
rno04310	Wnt signaling pathway	11	164	0.04246
rno04540	Gap junction	7	87	0.04302
rno05200	Pathways in cancer	28	537	0.04384
rno03430	Mismatch repair	3	22	0.04670
rno04530	Tight junction	11	167	0.04735
rno05140	Leishmaniasis	6	71	0.04826
rno00240	Pyrimidine metabolism	5	54	0.04970

Table S8 GO enrichment analysis of the target genes of miR-346

ID	Term	ListHits	PopHits	*p* value
GO:0045668	negative regulation of osteoblast differentiation	10	53	0.00015
GO:0048701	embryonic cranial skeleton morphogenesis	8	37	0.00025
GO:0045814	negative regulation of gene expression, epigenetic	5	15	0.00044
GO:0008157	protein phosphatase 1 binding	7	32	0.00057
GO:0016607	nuclear speck	32	370	0.00061
GO:0072542	protein phosphatase activator activity	4	10	0.00079
GO:0035308	negative regulation of protein dephosphorylation	4	11	0.00119
GO:0019888	protein phosphatase regulator activity	7	37	0.00142
GO:0001709	cell fate determination	5	19	0.00147
GO:0003682	chromatin binding	40	522	0.00149
GO:0009880	embryonic pattern specification	6	28	0.00158
GO:0005247	voltage-gated chloride channel activity	4	12	0.00172
GO:1903146	regulation of autophagy of mitochondrion	4	12	0.00172
GO:0070728	leucine binding	3	6	0.00182
GO:0005770	late endosome	16	151	0.00187
GO:0044305	calyx of Held	6	29	0.00192
GO:0043005	neuron projection	39	513	0.00195
GO:0005654	nucleoplasm	168	2926	0.00196
GO:0033619	membrane protein proteolysis	4	13	0.00240
GO:0001223	transcription coactivator binding	6	31	0.00274
GO:0005765	lysosomal membrane	18	187	0.00294
GO:0070292	N-acylphosphatidylethanolamine metabolic process	3	7	0.00308
GO:0043023	ribosomal large subunit binding	4	14	0.00324
GO:0032006	regulation of TOR signaling	5	23	0.00365
GO:0030165	PDZ domain binding	13	120	0.00397
GO:0005737	cytoplasm	270	5042	0.00414
GO:0048024	regulation of mRNA splicing, via spliceosome	4	15	0.00425
GO:0007369	gastrulation	6	34	0.00445
GO:0060463	lung lobe morphogenesis	3	8	0.00475
GO:0071144	heteromeric SMAD protein complex	3	8	0.00475
GO:1905665	positive regulation of calcium ion import across plasma membrane	3	8	0.00475
GO:0006829	zinc ion transport	4	16	0.00546
GO:0034399	nuclear periphery	4	16	0.00546
GO:0060395	SMAD protein signal transduction	9	71	0.00560
GO:0031146	SCF-dependent proteasomal ubiquitin-dependent protein catabolic process	7	47	0.00579
GO:0140297	DNA-binding transcription factor binding	11	98	0.00593
GO:0060349	bone morphogenesis	6	36	0.00595
GO:0034198	cellular response to amino acid starvation	7	48	0.00651
GO:0019104	DNA N-glycosylase activity	3	9	0.00688
GO:0032872	regulation of stress-activated MAPK cascade	3	9	0.00688
GO:0098919	structural constituent of postsynaptic density	3	9	0.00688
GO:0033630	positive regulation of cell adhesion mediated by integrin	4	17	0.00688
GO:0035094	response to nicotine	8	61	0.00717
GO:0005085	guanyl-nucleotide exchange factor activity	18	204	0.00727
GO:0007411	axon guidance	16	175	0.00794
GO:0071257	cellular response to electrical stimulus	4	18	0.00853
GO:0003149	membranous septum morphogenesis	3	10	0.00949
GO:0045050	protein insertion into ER membrane by stop-transfer membrane-anchor sequence	3	10	0.00949
GO:0090309	positive regulation of DNA methylation-dependent heterochromatin assembly	3	10	0.00949
GO:0034451	centriolar satellite	11	105	0.00985
GO:0043025	neuronal cell body	42	619	0.00986

Table S9 KEGG enrichment analysis of the target genes of miR-346

ID	Term	ListHits	PopHits	*p* value
rno04710	Circadian rhythm	6	30	0.00094
rno04520	Adherens junction	9	72	0.00193
rno04218	Cellular senescence	16	185	0.00252
rno04144	Endocytosis	21	275	0.00272
rno04514	Cell adhesion molecules	15	172	0.00314
rno04350	TGF-beta signaling pathway	10	94	0.00372
rno04310	Wnt signaling pathway	14	164	0.00514
rno04728	Dopaminergic synapse	12	132	0.00568
rno00410	beta-Alanine metabolism	5	30	0.00578
rno04933	AGE-RAGE signaling pathway in diabetic complications	10	101	0.00621
rno04659	Th17 cell differentiation	10	103	0.00712
rno04150	mTOR signaling pathway	13	157	0.00883
rno04024	cAMP signaling pathway	16	212	0.00934
rno04931	Insulin resistance	10	110	0.01112
rno05226	Gastric cancer	12	150	0.01500
rno04340	Hedgehog signaling pathway	6	52	0.01568
rno05216	Thyroid cancer	5	38	0.01577
rno05031	Amphetamine addiction	7	68	0.01693
rno04014	Ras signaling pathway	16	231	0.01981
rno05161	Hepatitis B	12	157	0.02078
rno04550	Signaling pathways regulating pluripotency of stem cells	11	142	0.02403
rno04211	Longevity regulating pathway	8	90	0.02485
rno05212	Pancreatic cancer	7	75	0.02752
rno05321	Inflammatory bowel disease	6	61	0.03195
rno05220	Chronic myeloid leukemia	7	78	0.03319
rno05217	Basal cell carcinoma	6	62	0.03425
rno05030	Cocaine addiction	5	47	0.03626
rno04916	Melanogenesis	8	99	0.04067
rno04921	Oxytocin signaling pathway	11	155	0.04176
rno00531	Glycosaminoglycan degradation	3	20	0.04183
rno00532	Glycosaminoglycan biosynthesis - chondroitin sulfate / dermatan sulfate	3	20	0.04183
rno05170	Human immunodeficiency virus 1 infection	15	234	0.04264
rno04390	Hippo signaling pathway	11	158	0.04688
rno05225	Hepatocellular carcinoma	12	178	0.04790
rno04020	Calcium signaling pathway	15	238	0.04822

Figure S1. Injection site in the RVLM. RVLM: rostral ventrolateral medulla.

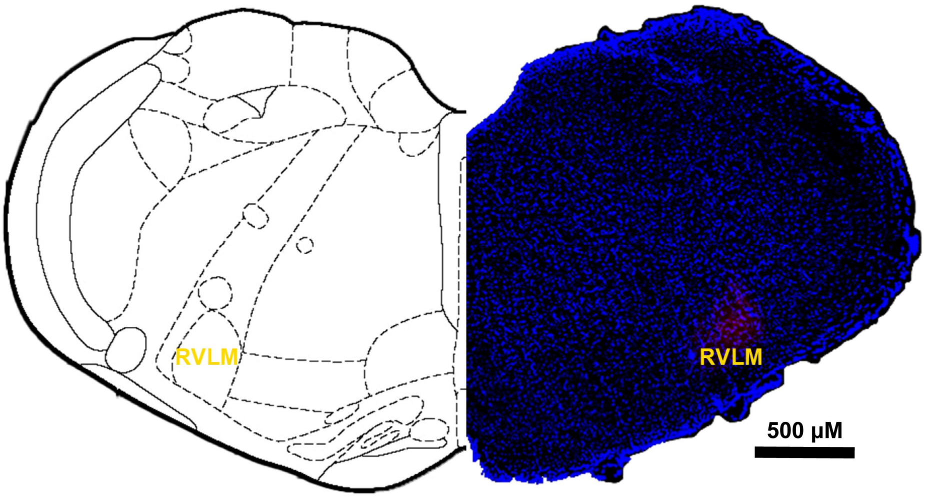

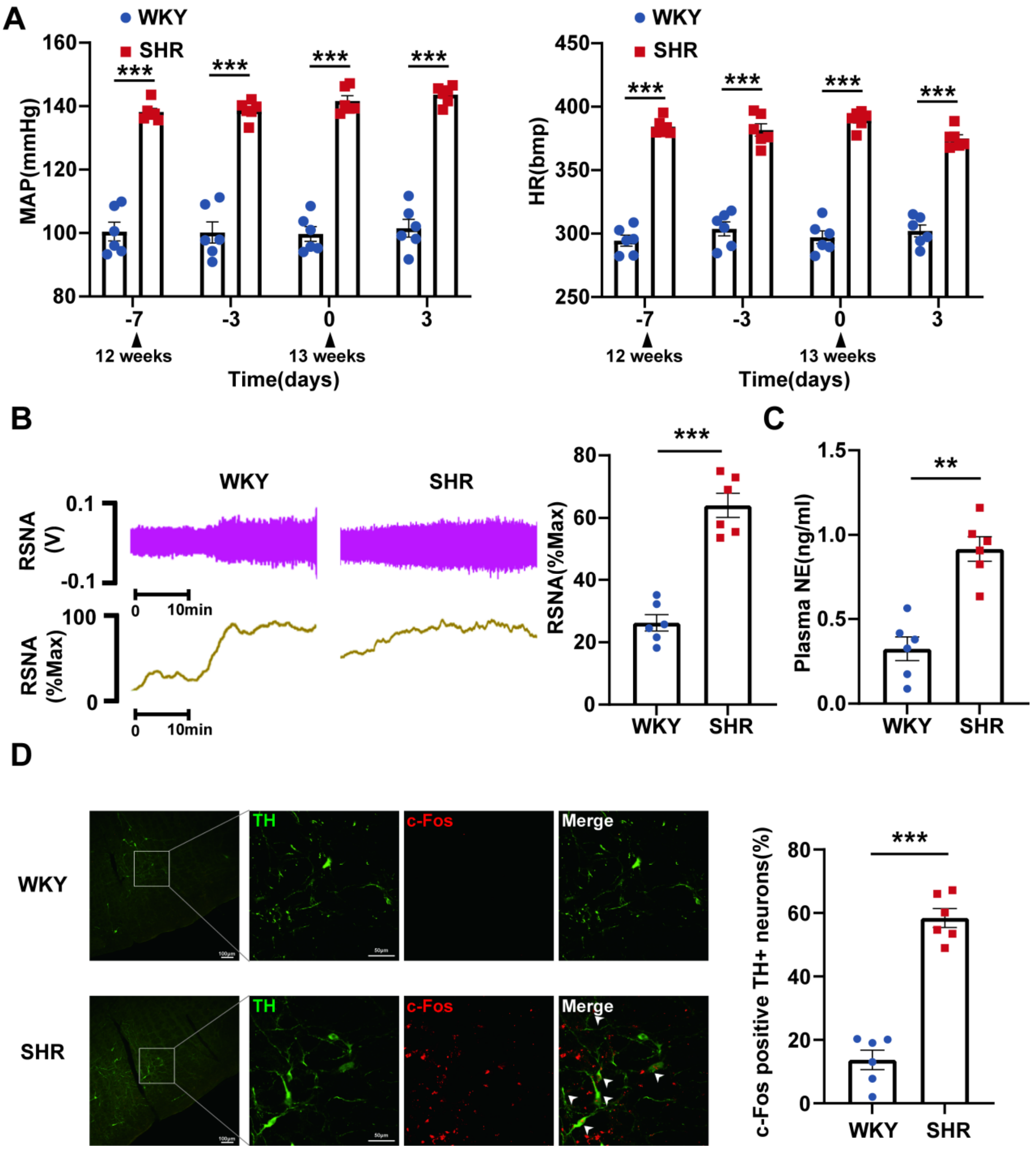

Figure S2. A–D, SHRs exhibited significant increases in MAP, HR, RSNA, plasma NE, and the population of c-Fos-positive TH+ RVLM neurons. n = 6 rats per group (**A–D**). $**p < 0.01$, $***p < 0.001$. HR: heart rate; MAP: mean arterial pressure; NE: norepinephrine; RSNA: renal sympathetic nerve activity; RVLM: rostral ventrolateral medulla; SHR: spontaneously hypertensive rat; TH: tyrosine hydroxylase; WKY: Wistar Kyoto.

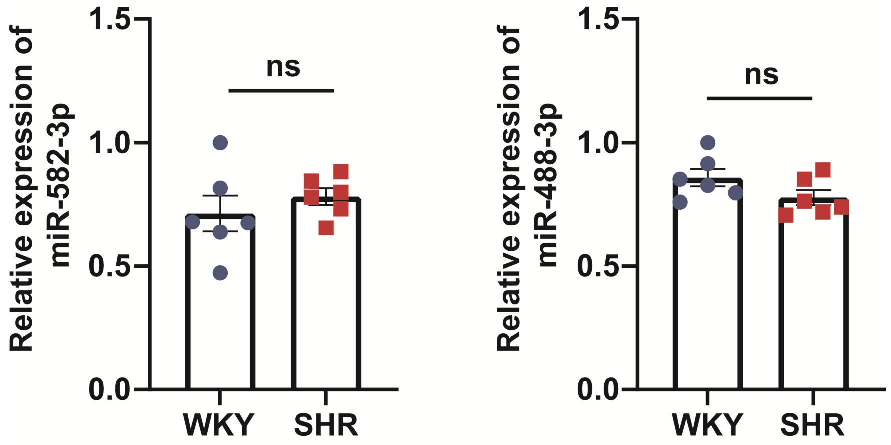

Figure S3. RT-qPCR was performed to determine the relative expression of two specific miRNAs indicated in Figure 1B. n = 6 rats per group. ns represents nonsignificant. RT-qPCR: reverse transcription quantitative polymerase chain reaction; SHR: spontaneously hypertensive rat; WKY: Wistar Kyoto.

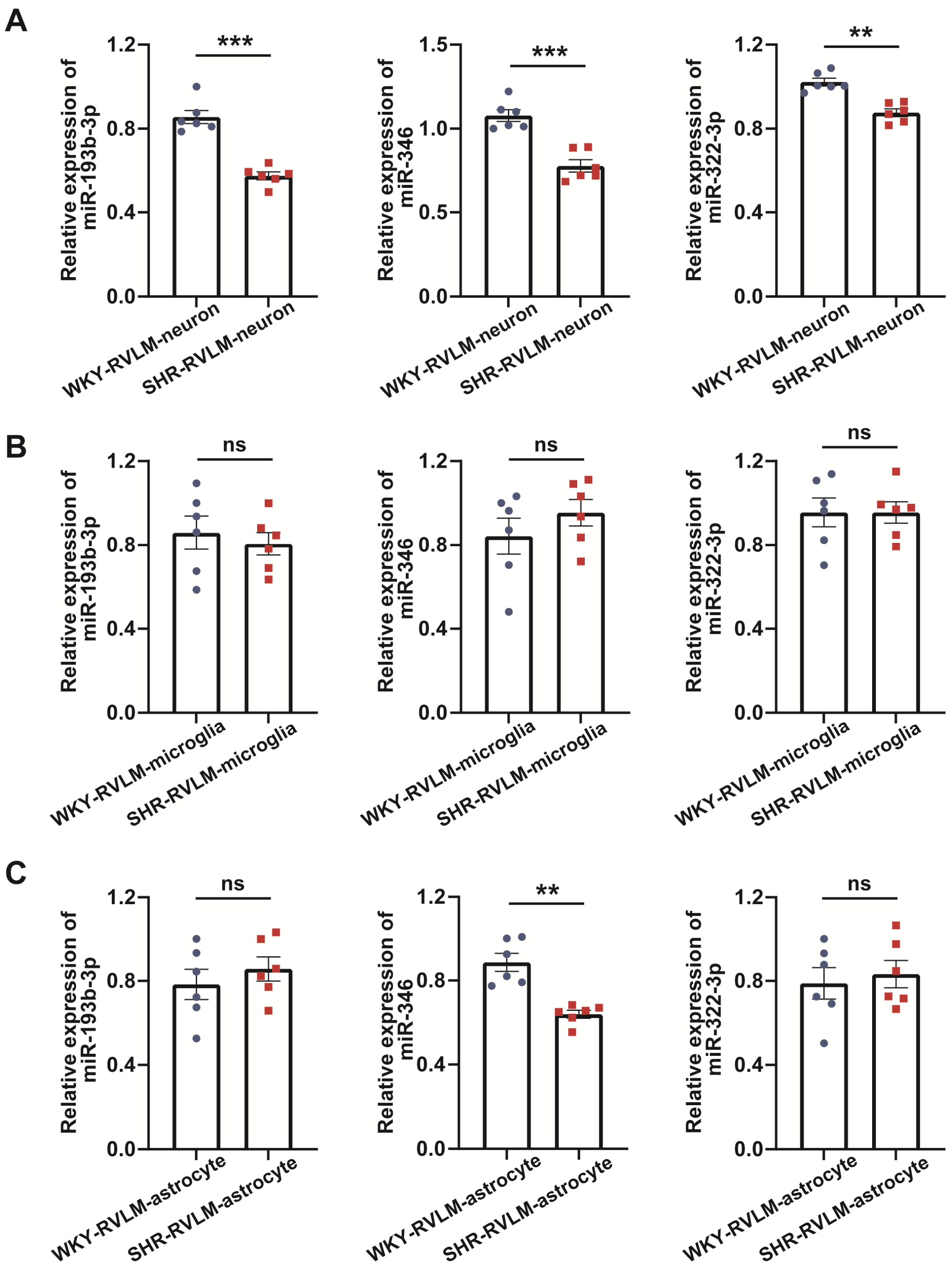

Figure S4. RT-qPCR was utilized to detect the differential expression of miR-193b-3p, miR-346, and miR-322-3p in neurons (**A**), microglia (**B**) and astrocytes (**C**) isolated from the RVLM tissues of 13 weeks old SHRs and WKY rats. n = 6 rats per group (**A**–**C**). $^{**}p < 0.01$, $^{***}p < 0.001$, ns represents nonsignificant. RT-qPCR: reverse transcription quantitative polymerase chain reaction; RVLM: rostral ventrolateral medulla; SHR: spontaneously hypertensive rat; WKY: Wistar Kyoto.

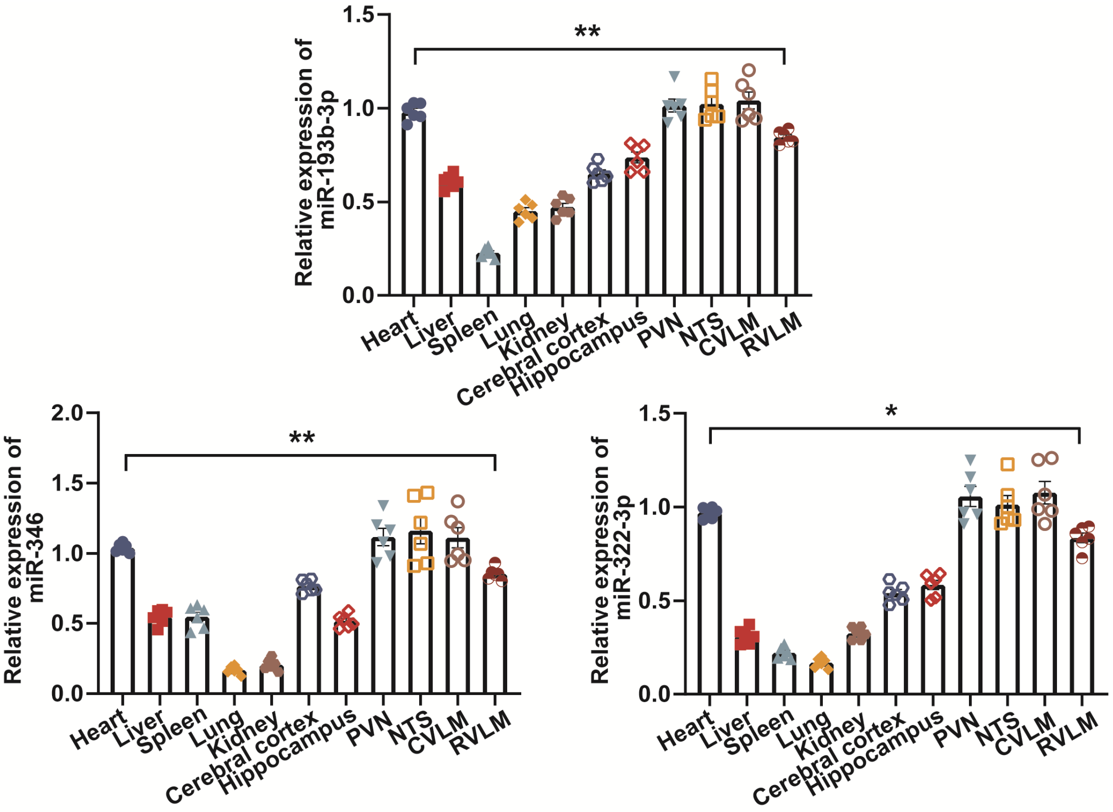

Figure S5. Expression levels of miR-193b-3p, miR-346, and miR-322-3p in 11 tissues of SHRs were detected using RT-qPCR. n = 6 rats per group. *$p < 0.05$, **$p < 0.01$. CVLM: caudal ventrolateral medulla; NTS: nucleus tractus solitarius; PVN: paraventricular nucleus; RT-qPCR: reverse transcription quantitative polymerase chain reaction; RVLM: rostral ventrolateral medulla; SHR: spontaneously hypertensive rat.

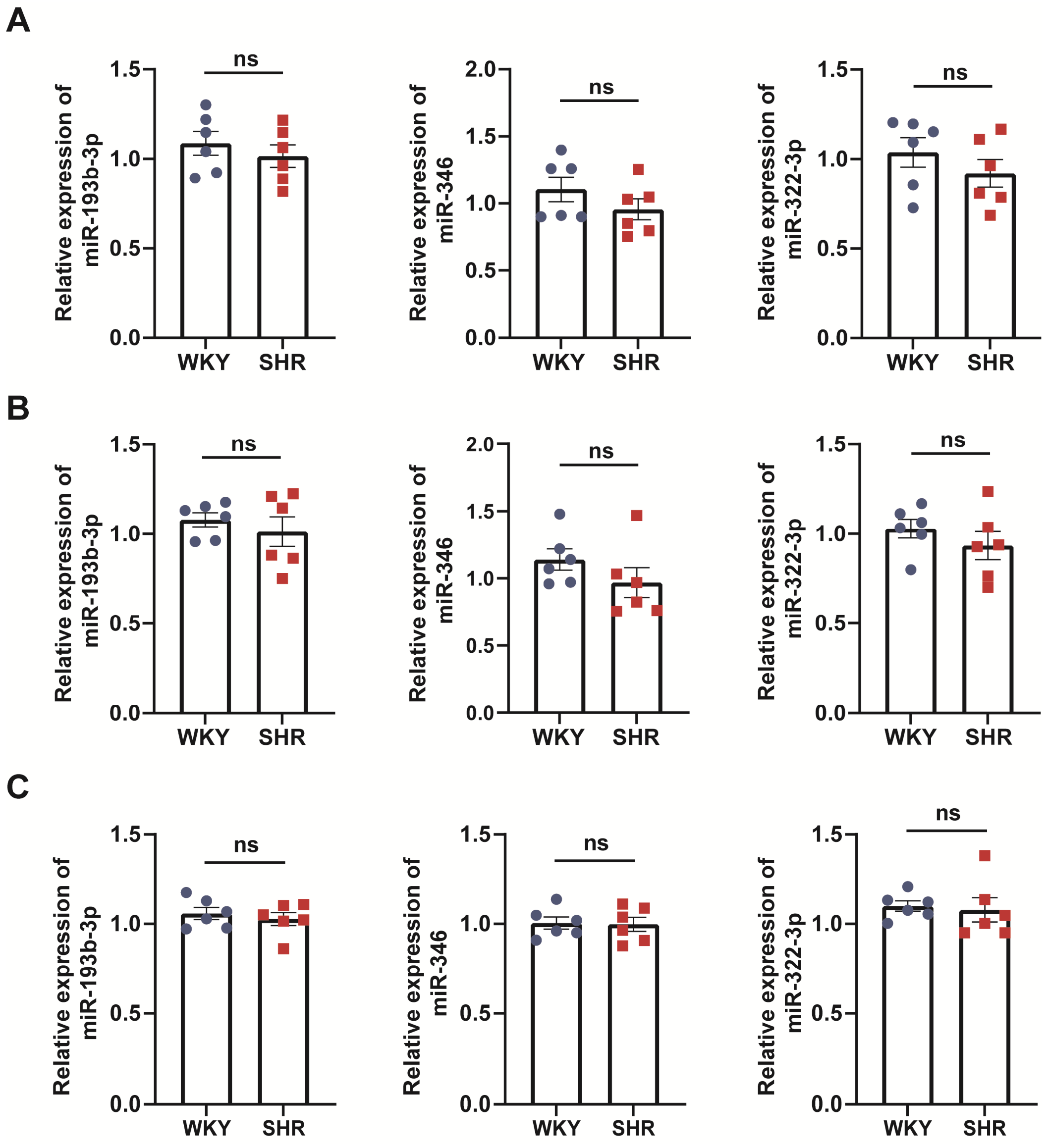

Figure S6. RT-qPCR was utilized to detect the differential expression of miR-193b-3p, miR-346, and miR-322-3p in NTS (**A**), CVLM (**B**) and PVN (**C**) between the two groups. n = 6 rats per group (**A**–**C**). ns represents nonsignificant. CVLM: caudal ventrolateral medulla; NTS: nucleus tractus solitarius; PVN: paraventricular nucleus; RT-qPCR: reverse transcription quantitative polymerase chain reaction; SHR: spontaneously hypertensive rat; WKY: Wistar Kyoto.

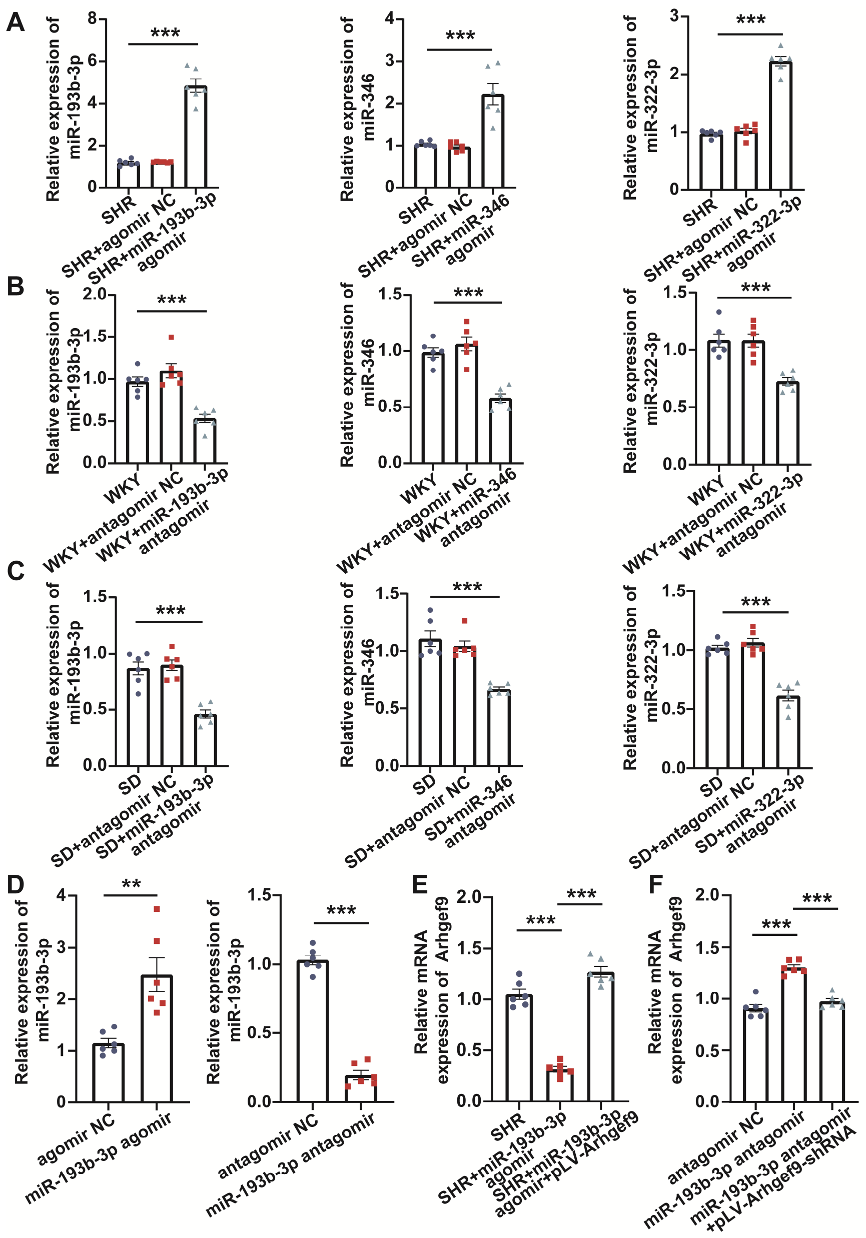

Figure S7. A, Expression levels of miR-193b-3p, miR-346, and miR-322-3p were determined by RT-qPCR in the RVLM of SHRs following agomir microinjection. **B,** RT-qPCR was performed to measure the expression of miR-193b-3p, miR-346, and miR-322-3p in the RVLM of WKY rats after administration of antagomirs. **C,** After antagomirs were administered, the expression levels of miR-193b-3p, miR-346, and miR-322-3p in the RVLM of SD rats were assessed using RT-qPCR. **D,** Relative expression of miR-193b-3p in RVLM primary neurons transfected with miR-193b-3p agomir or antagomir was determined. **E,** RT-qPCR was employed to analyze the expression of Arhgef9 in RVLM of SHRs after administering pLV-Arhgef9 plasmid and miR-193b-3p agomir. **F,** Expression of Arhgef9 was evaluated by RT-qPCR in the RVLM primary neurons after transfection with miR-193b-3p antagomir and pLV-Arhgef9-shRNA. n = 6 rats per group (**A**–**C** and **E**). n = 6 of independent cell culture preparations (**D** and **F**). **$p < 0.01$, ***$p < 0.001$. NC: negative control; RT-qPCR: reverse transcription quantitative polymerase chain reaction; RVLM: rostral ventrolateral medulla; SD: Sprague–Dawley; SHR: spontaneously hypertensive rat; shRNA: short hairpin RNA; WKY: Wistar Kyoto.

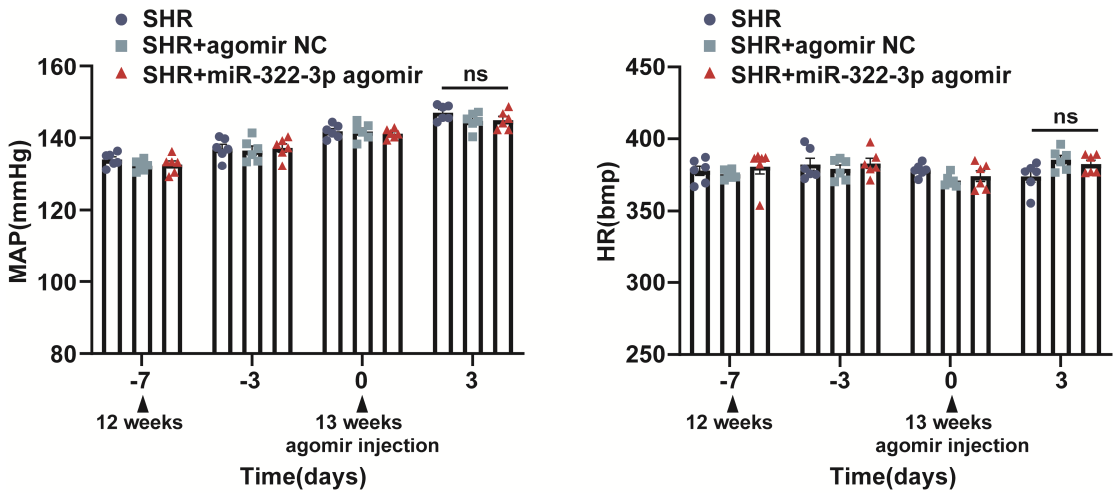

Figure S8. The involvement of miR-322-3p overexpression in the regulation of MAP and HR in SHRs was not observed. n = 6 rats per group. ns represents nonsignificant. HR: heart rate; MAP: mean arterial pressure; NC: negative control; SHR: spontaneously hypertensive rat.

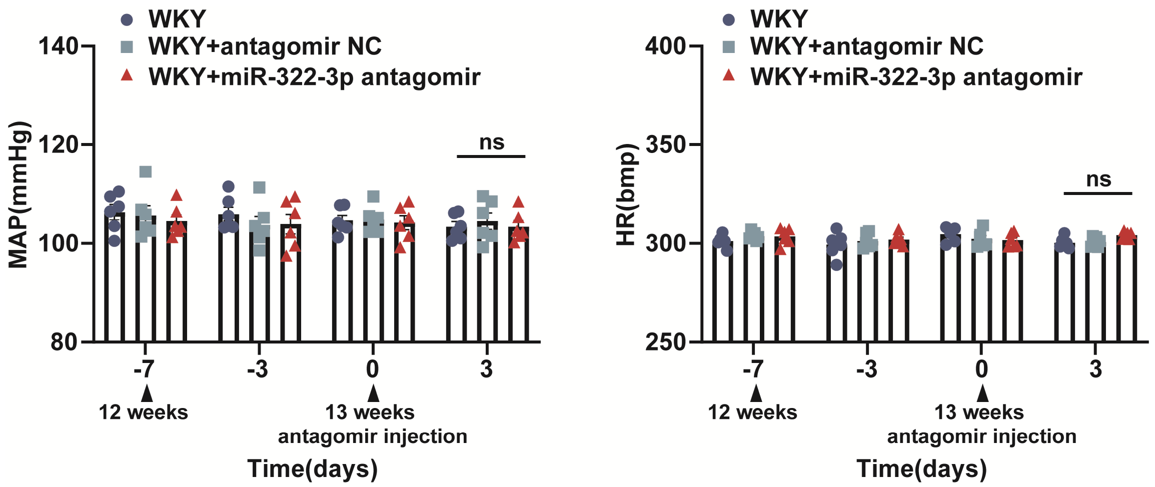

Figure S9. The MAP and HR of WKY rats remained unchanged following downregulation of miR-322-3p. n = 6 rats per group. ns represents nonsignificant. HR: heart rate; MAP: mean arterial pressure; NC: negative control; WKY: Wistar Kyoto.

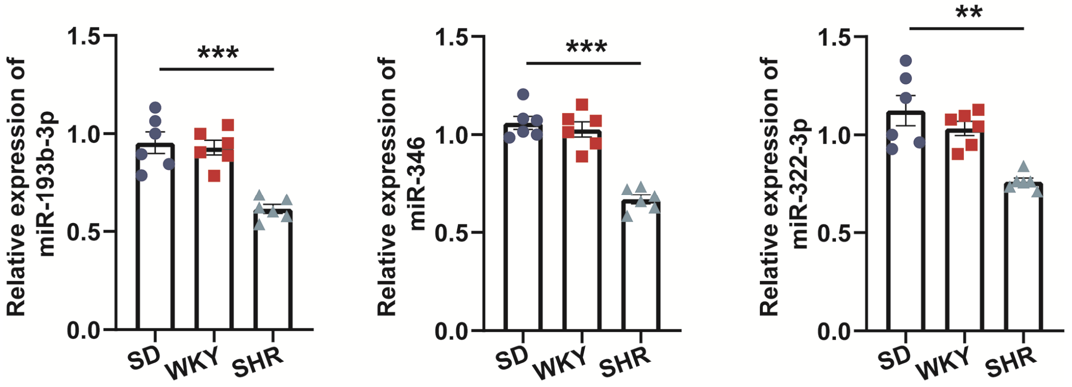

Figure S10. RT-qPCR was employed to identify the differential expression levels of miR-193b-3p, miR-346, and miR-322-3p in RVLM of SHR, WKY, and SD rats. n = 6 rats per group. **$p < 0.01$, ***$p < 0.001$. RT-qPCR: reverse transcription quantitative polymerase chain reaction; RVLM: rostral ventrolateral medulla; SD: Sprague–Dawley; SHR: spontaneously hypertensive rat; WKY: Wistar Kyoto.

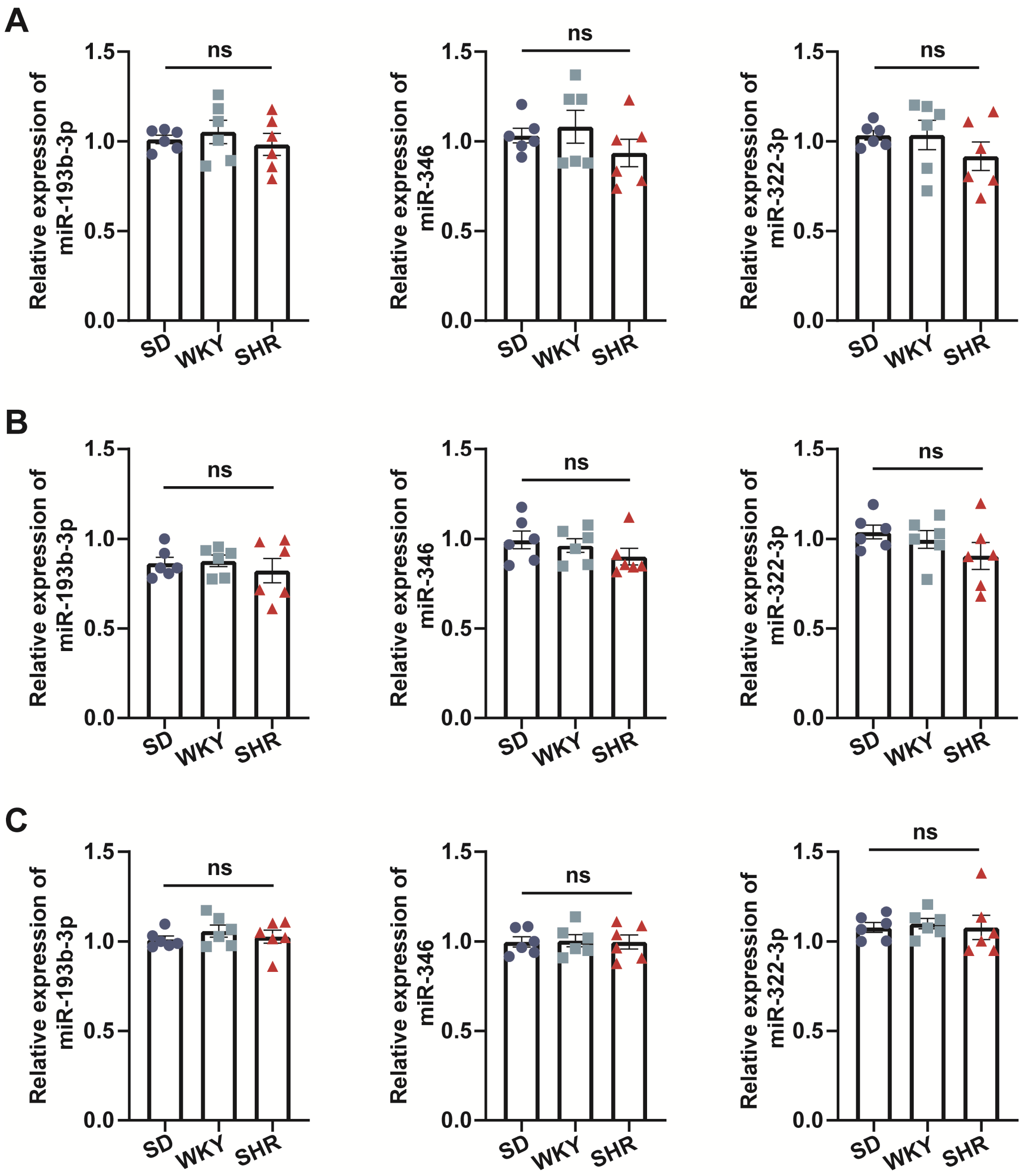

Figure S11. Differential expression of miR-193b-3p, miR-346, and miR-322-3p in NTS (**A**), CVLM (**B**) and PVN (**C**) among three groups was assessed by RT-qPCR. n = 6 rats per group (**A**–**C**). ns represents nonsignificant. CVLM: caudal ventrolateral medulla; NTS: nucleus tractus solitarius; PVN: paraventricular nucleus; RT-qPCR: reverse transcription quantitative polymerase chain reaction; SD: Sprague–Dawley; SHR: spontaneously hypertensive rat; WKY: Wistar Kyoto.

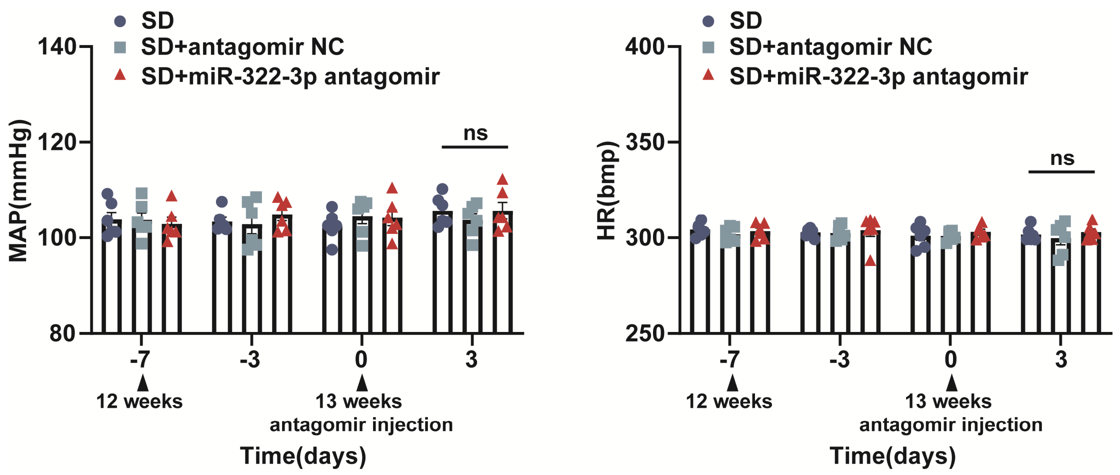

Figure S12. miR-322-3p knockdown had no significant effect on the MAP and HR of SD rats. n = 6 rats per group. ns represents nonsignificant. HR: heart rate; MAP: mean arterial pressure; NC: negative control; SD: Sprague–Dawley.

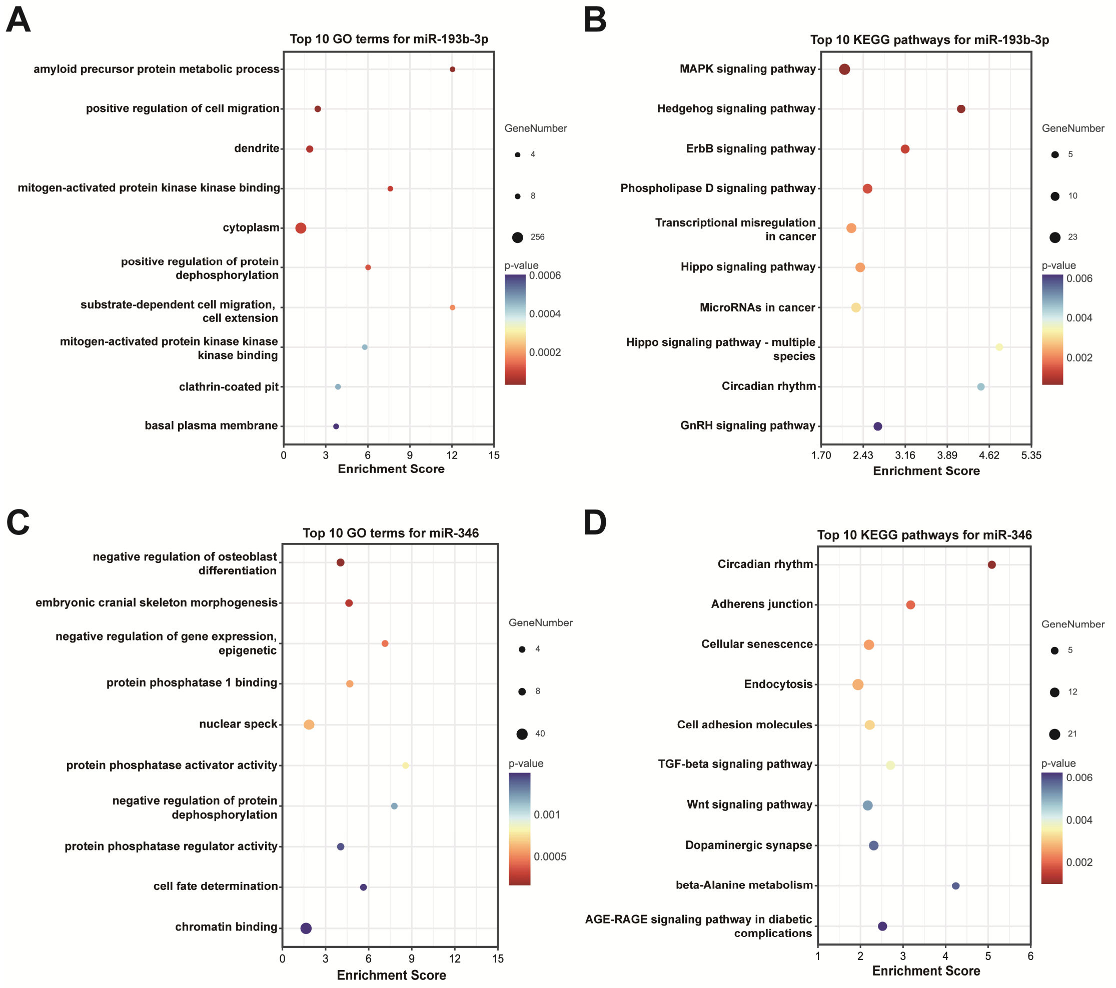

Figure S13. Bubble chart of GO terms and KEGG pathways for miR-193b-3p (**A** and **B**) and miR-346 (**C** and **D**) is shown. The size of the bubbles corresponds to the number of target genes involved, and the color of the bubbles represents statistical significance. GO: Gene Ontology; KEGG: Kyoto Encyclopedia of Genes and Genomes.

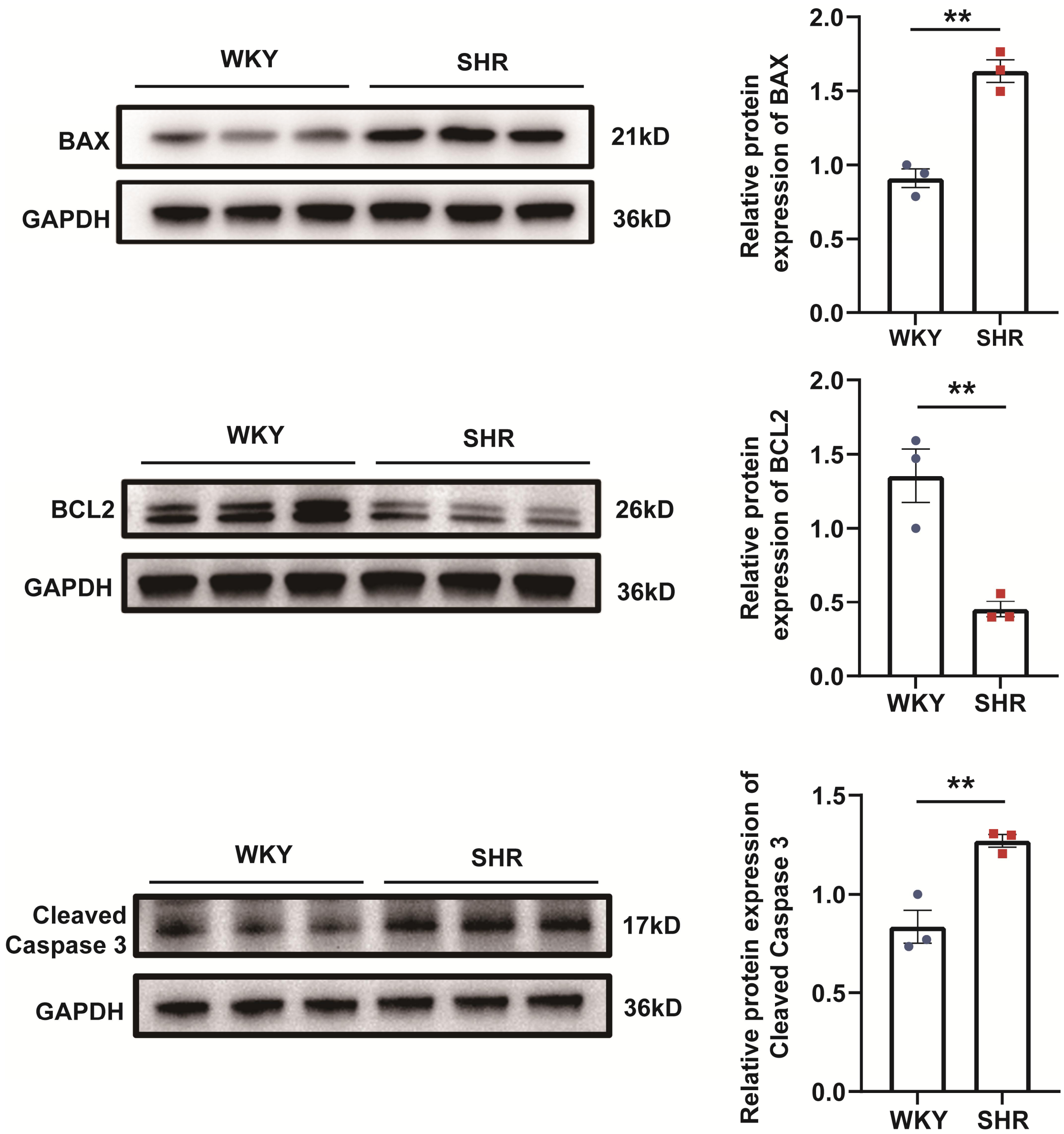

Figure S14. Expression levels of BAX, BCL2, and cleaved Caspase 3 were evaluated in RVLM of SHR and WKY rats through Western blot. n = 3 rats per group. $^{**}p < 0.01$. RVLM: rostral ventrolateral medulla; SHR: spontaneously hypertensive rat; WKY: Wistar Kyoto.

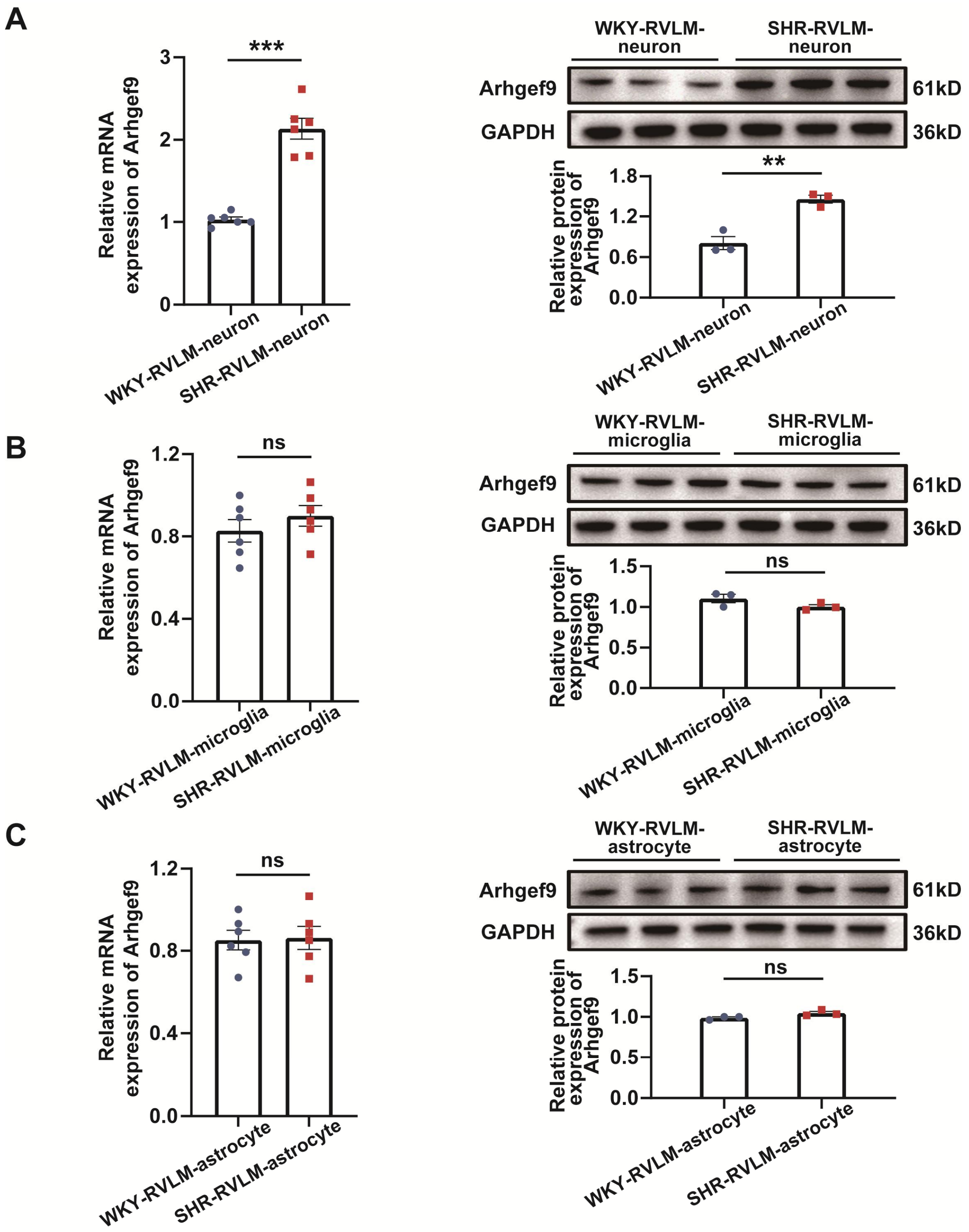

Figure S15. The mRNA and protein expression levels of Arhgef9 in neurons (**A**), microglia (**B**) and astrocytes (**C**) isolated from the RVLM tissues of 13 weeks old SHRs and WKY rats were evaluated through RT-qPCR and Western blot. n = 3–6 rats per group (**A**–**C**). $^{**}p < 0.01$, $^{***}p < 0.001$, ns represents nonsignificant. RT-qPCR: reverse transcription quantitative polymerase chain reaction; RVLM: rostral ventrolateral medulla; SHR: spontaneously hypertensive rat; WKY: Wistar Kyoto.
